# Critical Evaluation of Two Qualitative Analytical Approaches for Multiclass Determination of Veterinary Drugs in Bovine Muscle Using UHPLC-Q-Orbitrap: The Wind of Change in Brazilian Monitoring

**DOI:** 10.3390/molecules28104150

**Published:** 2023-05-17

**Authors:** Ramon Alves de Oliveira Paula, Carina de Souza Gondim, Eduardo Morgado Schmidt, Maria Helena Glicério Marcelina Diniz, Mary Ane Gonçalves Lana, Leandro Soares de Oliveira

**Affiliations:** 1 Postgraduate Program in Food Science, Department of Food Science (ALM), Faculty of Pharmacy (FAFAR), Federal University of Minas Gerais (UFMG), Campus da UFMG, Antônio Carlos Avenue 6627, Belo Horizonte 31270-010, Brazil; carinasgondim@gmail.com; 2 Nova Analítica Importações e Exportações LTDA, Assungui Street, 432, Vila Gumercindo, São Paulo 04131-000, Brazil; eduardomschmidt@yahoo.com; 3 Food of the Agricultural Defense Federal Laboratory of Minas Gerais, Ministry of Agriculture and Livestock, Rômulo Joviano Avenue, s/nº, Centro, Pedro Leopoldo 33600-000, Brazil; maria.diniz@agricultura.gov.br (M.H.G.M.D.); mary.lana@agricultura.gov.br (M.A.G.L.); 4 Department of Mechanical Engineering, Engineering School, Federal University of Minas Gerais (UFMG), Campus da UFMG, Antônio Carlos Avenue 6627, Belo Horizonte 31270-010, Brazil

**Keywords:** drug metabolites, food analysis, food analytical chemistry, food of animal origin, food safety, high-resolution mass spectrometry, meat matrices, multi-class multi-residue analysis, sample treatment, targeted-screening method

## Abstract

Food safety is recognized as a main requirement for consumers, food industries, and official laboratories. Here, we present the optimization and screening qualitative validation of two multianalyte methods in bovine muscle tissues by ultra-high-performance liquid chromatography coupled to high-resolution mass spectrometry with an Orbitrap-type analyzer, operated with a heated ionization source in positive and negative mode. This aims for not only the simultaneous detection of veterinary drugs regulated in Brazil but also the prospection of antimicrobials not yet monitored. Two different sample preparation procedures were applied: method A—generic solid-liquid extraction with 0.1% formic acid (*v*/*v*) in an aqueous solution of EDTA 0.1% (*w*/*v*)—acetonitrile-methanol (1:1:1, *v*/*v*/*v*), followed by an additional ultrasound-assisted extraction and method B—QuEChERS. In both procedures, selectivity showed satisfactory conformity. From a detection capability (CCβ) equivalent to ½ the maximum residue limit, >34% of the analyte resulted in a false positive rate of <5%, preponderant by the QuEChERS method, which exhibited a higher yield of the sample. The results showed the potential application of both procedures in the routine analysis of foods by official laboratories, enabling the expansion of this methodological portfolio as well as its analytical scopes, thus optimizing the control of residues of veterinary drugs in the country.

## 1. Introduction

Agricultural activity plays an extremely important role in the Brazilian economy. In 2021, for example, it reached the level of 27.4% of the national Gross Domestic Product (GDP), which was the highest since 2004 (27.5%) [1]. In the same year, 16.5% of Brazilian agribusiness exports corresponded to the meat sector [2], which consolidated Brazil as one of the largest producers of animal protein on the world stage. In this regard, considering beef exports in 2021 (12.04 million tons), the country occupies the first place in the global ranking, with 2.7 million tons (i.e., 22.3% of total exports) [3].

The growth scenario of this sector is expected to continue in the near future. This happens due to how even in the face of the effects of the pandemic and the restrictions resulting from COVID-19 between 2023 and 2031, world consumption of beef is expected to grow by 9.8%, resulting in a 47% increase in Brazilian exports of this commodity [4]. Likewise, the consumption of veterinary drugs, which in general is linked to animal production, will also increase, especially concerning antimicrobials, which would make Brazil the second consumer market for this in the world by 2030 [5].

In addition to presenting high commercial value, beef has nutritional diversity, such as high-quality proteins, minerals (e.g., iron, phosphorus, selenium, and zinc), and B complex vitamins, and is considered a complex food matrix, all of which have great importance in achieving a balanced diet and meeting the daily energy needs of individuals [6]. On the other hand, the presence of residues of veterinary drugs causes concern, whether for governments, industries, the scientific community, or consumers. They may include original compounds and/or their metabolites [7,8] above the maximum residue limits (MRLs), as well as have prohibited or unauthorized substances for the species. This is because exposure to such residues represents a potential health risk, causing antimicrobial resistance [9,10] and allergic reactions [11]. In addition, they can trigger trade restrictions [12,13,14] and impacts on the environment [15]. As such, it is imperative to strictly control these residues in foods of animal origin, aiming at their safety throughout the production chain. Thus, laboratory support is of great importance in achieving this goal.

In Brazil, agricultural defense activities have been carefully managed by the Ministry of Agriculture and Livestock (MAPA) for over 40 years [16] through a network of six official laboratories, currently called the Federal Agricultural Defense Laboratories (LFDAs), that are responsible for complying with the National Plan for the Control of Residues and Contaminants (PNCRC), by the analysis of representative test samples from federal inspections of meat and other matrices of animal origin [17,18]. The most official chemical methods applied in the routine analysis are quantitative and confirmatory, based on ultra-high performance liquid chromatography coupled to sequential mass spectrometry (UHPLC-MS/MS), combining high sensitivity and selectivity, which allows regulatory requirements to be met. However, most of these methods are directed to a relatively restricted number of analytes, classes, and pharmacological groups, focusing in most cases on those regulated [19,20,21,22]. Therefore, in order to reach a broader analytical scope, different methods are needed. These characteristics are associated with the limitations of LFDAs in infrastructure, human resources, and financial availability, highlighting the need to develop and implement more efficient methods targeting high analytical demand, both in number and types of matrices, as well as in analytes.

Considering the history of the PNCRC for the slaughtered bovine matrix, as seen in the year 2021, of the 3793 samples analyzed, only 0.26% showed results that did not comply with stipulated regulatory limits [17]. This result is in line with Martins et al. [23], who considered screening methods as a relevant strategy for routine analysis laboratories, given a prior expectation of non-compliance of some samples.

With these characteristics in mind, we report the optimization and validation of two qualitative analytical methods with different approaches of sample preparation based on high-resolution mass spectrometry (HRMS) for targeted analysis of multigroups of veterinary drugs in bovine muscle. Overall, we covered substances monitored in the PNCRC, including those for broiler chicken and swine, their metabolites, and growth promoter, in addition to the prospection of registered and available antimicrobials in the veterinary pharmaceutical market in Brazil and not yet monitored in the official inspection program in any species.

In the title of this paper, we provocatively declared: “the wind of change in Brazilian monitoring”, because as far as we know, there is no experimental research considering such a broad and ambitious scope that could make room for new perspectives in the field of modern food science. Finally, the application of those methods using HRMS through Orbitrap mass analyzers in a routine analysis is an important step towards increasing analytical capacity and maximizing the available resources. The methods presented here have an attractive set of analytical features, such as high acquisition speed, high sensitivity, high accuracy, and mass resolution [24], besides enabling many other potential functions, such as a retrospective surveillance analysis without the need to reanalyze the samples [25] and a non-targeted approach [26].

## 2. Results

### 2.1. UHPLC-Q-Orbitrap Analysis Optimization

Unsurprisingly, antimicrobials belonging to the class of aminoglycosides evaluated here (amikacin, apramycin, dihydrostreptomycin, spectinomycin, streptomycin, gentamicin, hygromycin B, kanamycin A, neomycin B, and tobramycin) showed unsatisfactory results. For analytes in other classes, the Acquity^TM^ UHPLC BEH C18 chromatographic column provided a reasonable runtime as well as an acceptable baseline separation performance. In addition, they presented regular results for resolution and peak shape in most compounds in a wide range of physicochemical properties (log *p* values and intrinsic solubility ranging from −4.569 to 7.877, and from −7.704 to 5.448, respectively, as listed in Table S4 of the Supplementary Materials), thus demonstrating better sensitivity. The respective retention times are shown in Table S5 which is included in Supplementary Materials.

**Figure 1:**
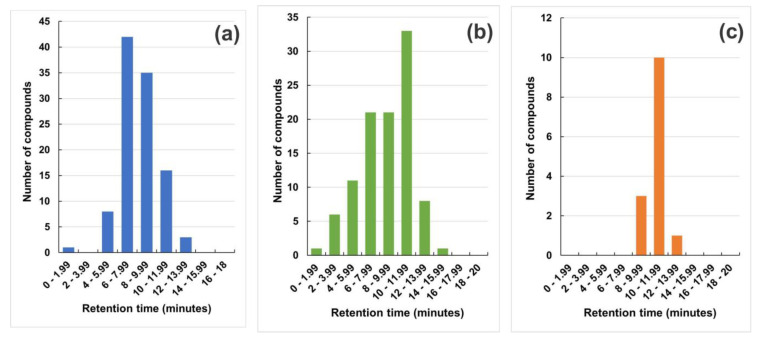
Distribution of veterinary drugs according to their retention times for methods A (**a**) and B, aqueous extract (**b**) and organic extract (**c**).

**Figure 2:**
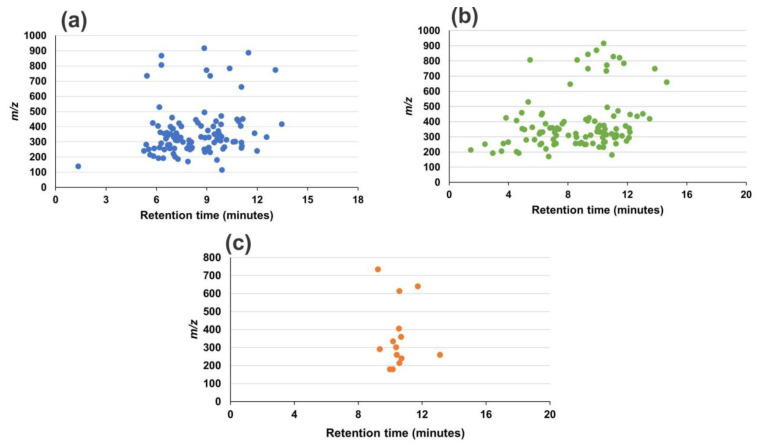
2D graph of *m*/*z* versus retention time for numerical distribution of veterinary drugs detected by method A (**a**) and by method B, aqueous extract (**b**) and organic extract (**c**).

Figure 1 shows the histograms with the distribution of veterinary drugs for method A (n = 106) and method B (n = 116) besides the aqueous and organic extract methods (102 and 14, respectively) from the respective chromatographic runs, with a retention time window of two minutes. Furthermore, to provide a glimpse of the task, Figure 2 displays 2D graphs of the distribution of these analytes by retention time, as well as the *m*/*z* values.

The chromatographic program employed projected a gradient in an execution time of 17 min for appropriate separation of the analytes of method A and 20 min in the two separation conditions of method B.

In general, in method B we highlight the existence of *m/z* values distributed in a dispersed way in close retention times (Figure 2), which can be related to the small interference from analyte to analyte. In this method (Figure 2b), we also observed the identification of some analytes of greater polarity at the beginning of the chromatographic run.

The following 14 analytes provided the best responses using a reconstitution solution containing 95% acetonitrile (mobile phase B): tildipirosin (log *p* 4.46), novobiocin (log *p* 3.41), 7-chloro-8-quinolinol (log *p* 2.43), 5,7-dichloro-8-quinolinol/chloroxine (log *p* 3.04), 5-chloro-8-hydroxyquinoline (log *p* 2.43), diclazuril (log *p* 4.23), lasalocid A (log *p* 7.67), 4,4′-dinitrocarbanilide (log *p* 3.00), robenidine (log *p* 3.99), trimethoprim (log *p* 1.28), mefenamic acid (log *p* 4.40), tolfenamic acid (log *p* 5.49), and moxidectin (log *p* 5.67) e triclabendazole (log *p* 5.88).

In turn, the ions formed by the protonated molecules [M+H]^+^, in both methods, were those predominant in the MS full scan spectra, present in (Table S5 Supplementary Materials). The use of ammonium formate did not favor the formation of adducts with the ammonium ion [M+NH_4_]^+^ in either method, using the source temperature at 350 °C. For ethopabate, salinomycin, and virginiamycin M_1_, among others in method A, the cationized ions [M+Na]^+^ were the most abundant.

For crystal violet and malachite green dyes, we emphasize that they are already ionized, as they are salts (hychlorides) containing nitrogen with a positive charge. Therefore, the mass [M]^+^ of these compounds was detected. For erythromycin (method B), the [M-H_2_O+H]^+^. For dicloxacillin (method A), cloxacillin (method B), nafcillin (method A), as well as oxacillin and phenoxymethylpenicillin (method B), the ions [(M+H_2_-CO)+H]^+^ were identified.

Other molecular ions with different charges produced better responses: [M+ 2H]^2+^ for spiramycin I (method A); [(M-H-CH_3_OH)]^+^ for dicloxacillin (method A) as well as cloxacillin (method B). In addition, deprotonated precursor ions [M-H]^−^ were selected for some analytes, for example, 4,4′-dinitrocarbanilide, florfenicol, thiamphenicol, closantel, and fipronil. Unlike the others sulfonamidas, phthalylsulfathiazole, sulfamerazine, sulfadoxine, and sulfacetamide were more sensitive in negative mode.

### 2.2. Extraction and Cleanup Procedures

Table 1 presents an evaluation of the two analytical procedures on bovine muscle samples proposed in this study, in terms of technique complexity/time expenditure, sample weight, consumption of solvent and solutions, and amount of adsorbent.

In this research, a great extraction capacity for the analytes was evidenced by the B method, in which 116 veterinary drugs were detected, in contrast to the 106 of method A. It should be emphasized that through visual inspection, the extracts of both methodologies were clear; however, the results of the QuEChERS method suggest that it provided a more significant cleanup, generating reduced matrix effects and better validation results at the lowest enriched level for most analytes.

### 2.3. Method Validation

#### 2.3.1. Selectivity/Specificity

The selectivity/specificity of the methods was ensured, as the negative samples were successfully differentiated through an inspection of the chromatograms of the 20 bovine muscle blank samples, which did not show any matrix interference in the retention time of our analytes that could generate a false positive signal, compromising the identification of each target compound.

#### 2.3.2. Detection Capability (CCβ)

Considering the CCβ criterion for screening of 0.5 × MRL, 68 analytes (43.3%) were properly identified and considered fully validated in method B and 54 (34.4%) in method A.

Table 2 presents the validation results for the multiclass screening of veterinary drugs, based on the selectivity and CCβ parameters evaluated at different proportions of the MRL of each analyte in 20 samples, for method A and method B.

**Figure 3:**
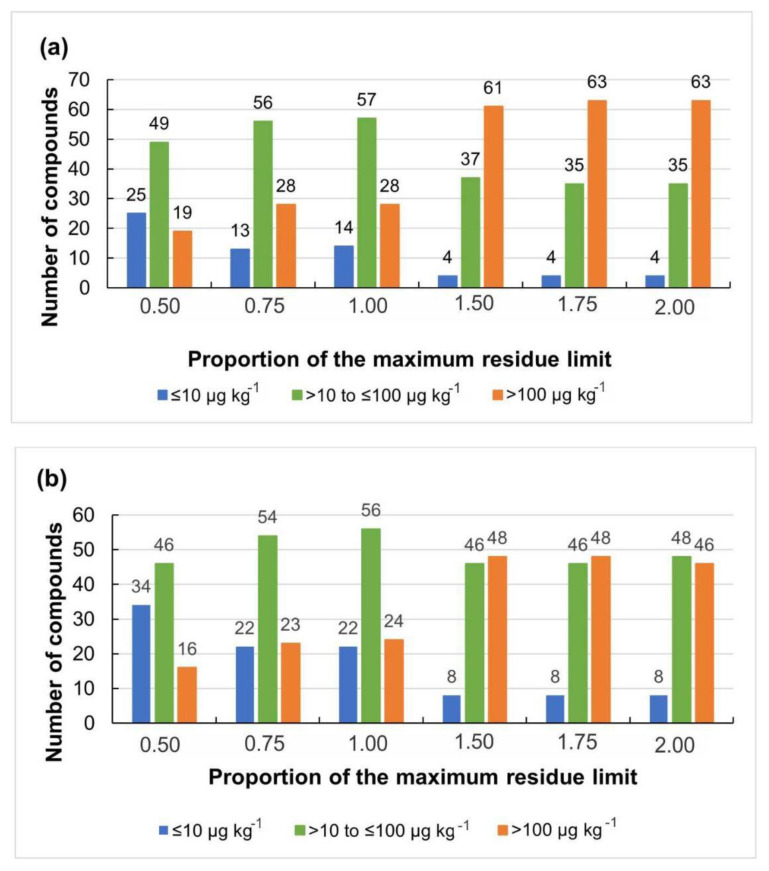
Distribution of analytes by proportion of the maximum residue limit versus the number of analytes detected and identified by concentration ranges for the evaluated methods (**a**) method A and (**b**) method B.

Figure 3 shows the number of analytes detected and identified by concentration ranges and MRL ratio (0.5 to 2.0 MRL) for method A and method B.

## 3. Discussion

### 3.1. UHPLC-Q-Orbitrap Analysis Optimization

Based on analytical performance, Jongedijk et al. [27] described the significant growth of the application of the technique for the analysis of residues of veterinary drugs and hormones in recent years, especially in the screening of multiple residues. This is a field that already has a high degree of confidence in its applicability in several European laboratories. These authors point out that, from a total of 35 European Union and National Reference Laboratories in the field of waste in 30 countries, 58% use HRMS for various purposes and motivations, and 50% use Orbitrap. Given the change in the national and international panoramas for food safety, that is, risk-based and not just routine monitoring, the HRMS would be a crucial link to the progress of these programs. Therefore, its under-exploitation in the control of residues of veterinary drugs may be linked not only to the absence of daily routine in laboratories but also to the lack of awareness of its added value for important controls.

In the review of Spisso, Pereira & Ferreira [28], the inclusion of antimicrobials belonging to the class of aminoglycosides in multiclass analytical methods was reinforced to still be a challenge, due to the high polarity of these molecules, which generally requires specific chromatographic conditions. Considering log *p* values as limits to distinguish analytes into polarity/lipophilicity groups, such as those established by Hepperle et al. [29], where high polarity compounds (low lipophilicity) are those with (log *p* < 2.5); intermediate (2.5 ≤ log *p* < 4) and low polarity (log *p* ≥ 4) aminoglycosides are highly hydrophilic, presenting Log *p* below −2 (−2.08 a −8.58) and pKa values between 8.38 to 10.91. In addition, they have a large amount of amino and hydroxyl groups in their structures, which makes their separation by conventional reversed-phase liquid chromatography complex (chemical properties of the C18 column), without ion pairing. This is because, in this mechanism, highly polar compounds present early elution (close to dead volume) in traditional reversed-phase stationary phases, causing low detection sensitivity by mass spectrometry [30], used in water/methanol mobile phases (method A), ammonium formate 5 mmol L^−1^/water, water/acetonitrile (method B). An additional issue to be considered is that according to the conditions of ionization and evaporation of the ion, a suppression or ionic increase in the formation of ions of the analyte may occur, the suppression being higher in polar compounds [31]. This fact indicates the need for specific analytical methods, either for sample treatment or chromatographic separation [32].

It is important to point out that in method B the same aqueous and organic mobile phase compositions found in the Ref. [22]. These authors reported that in their optimization experiments, the composition of mobile phase A (ammonium formate 5 mmol L^−1^ in water with 0.1% formic acid) provided a broader separation, with a more satisfactory peak formation and signal intensity, compared to those carried out in water:acetonitrile (95:5, *v*/*v*) with 0.1% of formic acid, thus allowing the ionization of analytes from the avermectin class.

Both in methods A and B, we avoided short chromatographic runs in order to curb possible problems, such as interference from the overlapping of analytes. Furthermore, as the gradient initiates the chromatographic run with high aqueous content, it becomes indispensable in complex matrices. At first, there is an elution of hydrophilic substances that may interfere with the matrix, making coelution difficult with any analyte. Moreover, a high number of organic reagents at the end of the gradient, in addition to relatively long wash intervals, enables a satisfactory cleaning of strongly retained substances and poorly eluted matrix constituents, making drag phenomena difficult in the column [23,33].

Considering the intention of using a comprehensive screening method, with reduced analytical times and a high number of samples analyzed per laboratory day, we applied positive and negative electrospray ionization in the same run, without exacerbating the number of scans (loss of sensitivity) and of the satisfactory acquisition data points per peak (≥8). This strategy was also adopted by other authors, such as Sardela et al. [34].

On C18 reversed phase columns, those substances with retention times below 1 (one) minute are extremely susceptible to the reconstitution solvent. Thus, such a solvent needs similarity to the initial mobile phase of the chromatographic run [34]. It is noteworthy that, in the method proposed by Silva and co-authors [22], a critical point noted in the extraction procedure was the final resuspension of the extract after evaporation. These authors, when analyzing the responses of the analytes in the extract reconstituted with mobile phase A, observed low linearity for antiparasitics belonging to the class of avermectins, triclabendazole, and closantel. This may be linked to the solubility of the more apolar analytes, affected by the increase in the proportion of aqueous phase in the medium. Therefore, the organic phase proportion (acetonitrile) was increased in the reconstitution solution to aid in the solubilization of the more lipophilic analytes, bringing better results for these substances. Recently, authors from elsewhere (Ref. [35]) reported the same strategy. This situation justified the division of the extract into two aliquots before evaporation: the first aliquot resuspended after total evaporation in mobile phase A; and the second aliquot resuspended in mobile phase B, which consisted of 95% acetonitrile.

Aiming at being comprehensive and maintaining the detectability of the most apolar analytes, we also followed this preparation. Although it provided satisfactory responses for analytes of the avermectin class only in the detectability of emamectin B1a (log *p* 6.72) and moxidectin (log *p* 5.67), the best responses were obtained for the provided 14 analytes cited in Section 2.3.

Pugajeva et al. [36] observed that, for analytes that predominantly form sodium adducts during ionization, such as cocciodiostats and avermectins, the most delicate step is the reconstitution of evaporated samples. For these authors, the reconstitution only in an acid solution of water/acetonitrile led to reduced signals, due to the unsatisfactory ionization of the molecules under the adopted conditions. The studies of Uczay et al. [37] showed that among avermectins, abamectin, and eprinomectin had lower sensitivity through the UHPLC-MS/MS analysis.

As already expected, due to the addition of formic acid in the mobile phase, the protonated ions [M+H]^+^ in both methods, were predominant. We believed that the existence of ammonium ions could prevent the formation of sodium adducts, which is more common in acidic conditions (formic acid) for some molecules avid for sodium; however, the ammonium formate of the mobile phase did not prevent the formation of [M+Na]^+^. In method A, the highest abundant [M+Na]^+^ ions for some analytes may be attributed to the presence of carboxylic groups and to the macrocycle effect of these molecules. It should be noted that this sodium adduct is avoided for two main reasons: its high stability and poor response to fragmentation [38].

In the case of crystal violet and malachite green dyes, which are ionized because they are salts (in this case hydrochlorides) and endowed with a nitrogen with a positive charge, the molecular ion [M]^+^ was detected without the addition of a proton. The [M-H_2_O+H]^+^ ion, observed for erythromycin in method B, refers to the rapid loss of H_2_O since the molecule is very unstable in acidic conditions, which favors its dehydration, as described by Sismotto et al. [39]. The [(M+H_2_-CO)+H]^+^ ions, identified for the penicillins dicloxacillin (method A), cloxacillin (method B), nafcillin (method A), as well as oxacillin and phenoxymethylpenicillin (method B), refer to their products of degradation formed when submitted to an opening of the β-lactam ring, as reported by Ref. [40].

For the sulfonamides phthalylsulfathiazole, sulfamerazine, sulfadoxine, and sulfacetamide that were identified by the negative ionization mode (Table S5, Supplementary Material), we believe that such behavior can be explained by substances’ pKa and by the resonance effects. As described by Qi et al. [41], the negative ionization mode [M-H]^−^ is more suitable when the substances have chlorine, bromine, sulfonic, carboxylic, or hydroxyl groups in the chain, in view of their deprotonated nature.

### 3.2. Extraction and Cleanup Procedures

The development of practices that, in addition to aiming at innovation, contemplate sustainability, i.e., the preservation of the environment, has become one of the main guidelines of the scientific community [42]. From this bias, our goal consisted in exploring qualitative screening methods with high yield and analytical frequency, as well as with low complexity and capability of contemplating the principles of green analytical chemistry. This may mean reducing the volume reagents, materials, and consequent generation of waste, as well as the simultaneous and multielementary determinations [43], the latter related to saving time, work, and cost per sample [44,45]. We highlight that in such methods we used what we had at our disposal (equipment, reagents, and supplies) so that they could be employed in the intense routine of an official Brazilian laboratory. This fact makes it possible, in addition to a reduction in operating costs, to maintain the health and safety of the analysts who may use it, an important stimulus for the application of the PNCRC monitoring program, considering the reduction in the number of samples required for the confirmatory analysis.

Kaufmann et al. [46] reported that modern methods of veterinary drugs usually examine more than 100 compounds, thus requiring the adoption of generic procedures, since multiclass compounds have very different physical and chemical properties. In this regard, the initial challenge in food analysis is the extraction of target compounds from the matrices [47]. Here, we selected the methodology proposed by Dasenaki et al. [48], who identified, in fish tissue, 143 veterinary pharmaceutical products using high-performance liquid chromatography quadrupole time-of-flight mass spectrometry (UHPLC-QTOF MS). Besides, we selected the method performed by this laboratory, proposed by Silva et al. [22] which, despite having identified and quantified 27 antiparasitic drugs of the benzimidazoles and nitroimidazoles classes, shows promise if adapted to a multigroup application. It may happen since the analyzed substances present a wide range of partition coefficient (log *p*): avermectins, varying from 5.56 to 6.724, for eprinomectin and emamectin; benzimidazoles varying from 1.566 to 7.713 for albendazole sulfoxide and closantel, and nitroimidazoles varying from −1.277 to 1.474 for hydroxymetronidazole and ipronidazole. It should be noted that the class of avermectins compromises the choice of a sample extractor and a considerable cleanup procedure [49].

We observed that in the analyses of drug residues in foods of animal origin, acetonitrile is the most recurrent solvent [50,51,52], enabling protein precipitation, enzymatic denaturation, minimization of co-extraction of matrix substances such as lipids, in addition to providing high extraction recoveries [53,54]. The scientific literature reports that the efficiency of extraction through acetonitrile can be greater when used in acidic conditions [55]. However, it has been suggested that this solvent does not sufficiently extract the polar analytes [56].

In turn, methanol can also be applied in this process and, in general, extract excess compounds from the matrix and it may overload the cleaning step [54]. On the other hand, in some cases, methanol may be suitable for achieving higher responses of β-lactams and tetracyclines [57]. It is worth noting that the use of formic acid in water is well established because it causes rapid degradation of monobasic penicillins [58]. In fact, Turnipseed et al. [49] used formic acid in acetonitrile as an extractor solvent at a concentration of 0.2 or 1% multiresidue analysis of veterinary drugs in salmon.

In many cases, the addition of EDTA to the extraction solution is considered positive as a strategy for its improvement and recovery of some analytes, especially tetracyclines, since such compounds can form complexes with bi and trivalent cations capable of compromising its extraction. This chelating agent disrupts these interactions by competing with such antimicrobials [59,60]. Beyond the tetracyclines, positive results have also been reported for benzimidazoles, sulfonamides, macrolides [60], and fluoroquinolones [61]. In the experiments of Xu et al. [62], the addition of Na_2_EDTA was used in the extraction procedure, as well as the exchange of MgSO_4_ for Na_2_SO_4_ to solve chelation problems. Besides the EDTA presence, we emphasize that the solubility of tetracyclines is higher in alcohols (more polar solvents) such as MeOH and ethanol, varying in other organic solvents, such as ethyl acetate (EtOAc), acetone, and ACN [63]. In method A, we highlight that both the addition of 2 mL of a solution formed by EDTA at 0.1% (*w*/*v*) in water with formic acid 0.1% (*v*/*v*) and the mixture of MeOH and ACN can have favored the extraction of this class of antimicrobials. On the other hand, this EDTA solution may have been responsible for a longer time in the extract evaporation stage to dryness, impacting sample preparation time when compared to method B, which needs to weigh salts, for example.

This combination of solvents and the final volume of 6 mL of extraction was used in the previous study of Dasenaki and Thomaidis [57]. These authors found the largest amount of aqueous solvent privileged the extraction of those more polar substances (log *p* < 2); for example, β-lactams antimicrobials, quinolones, and tetracyclines. On the other hand, they observed that the more hydrophobic analytes, i.e., non-hormonal anti-inflammatory drugs, coccidiostats, benzimidazoles, morantel, nitroxynil, and oxyclozanide, did not obtain an adequate recovery. Regarding non-hormonal anti-inflammatory drugs, some authors point out that their low recovery may be due to the absence of a hydrolysis step in the sample preparation since such substances bind to proteins due to the deconjugation of the analytes [64,65].

As a way to increase extraction efficiency, the use of ultrasonic energy enables chemical and physical transformations [66] and, through for example an ultrasonic bath, this can contribute to an efficient release of analytes from the matrix [57] by improving cell breakdown [67]. Kemmerich et al. [68] reported that an extraction accompanied by ultrasound for 15 min (30 °C/40 Hz) was crucial to cover a greater number of compounds, in addition to enhancing the recovery results, with good precision and without adding time to ultrasound.

Regarding the cleaning step, hexane is used to reduce the lipid content in the extract, by degreasing the sample [57] and, consequently, reducing such interferences in the analysis [59]. In a study developed by Rizetti et al. [69], the addition of hexane during extraction provided a reduction in the UHPLC-MS/MS response of macrocyclic lactones (avermectins and milbemycins), except for eprinomectin. This fact can be justified by the similarity of the polarity of the referred compounds with hexane since they are also non-polar. Furthermore, it may justify the non-detection of these compounds by method A in the present study.

For the partition at low temperatures, another cleaning strategy allows the separation of fatty co-extractives present in extracts from foods of animal origin [70], besides the precipitation of remaining proteins [57]. From the freezing of the aqueous phase, such solids (fat and proteins) are retained in the frozen phase [71].

Deep-freezing at −60 °C combined with subsequent refrigerated centrifugation was reported by Molognoni et al. [72]. Martins et al. [23] proposed refrigeration of the extract (−18 °C for 30 min). In turn, Oliveira et al. [73] adopted the temperature of −20 °C, for 1 h. We emphasize that the original method A, as described by Dasenaki et al. [48], stipulated a freezing temperature of −12 °C, for 12 h, for the fish matrix. Here, we successfully applied the reduction of the waiting time to 1h at the temperature of −80 °C, a great strategy for reducing procedure time.

The operation of QuEChERS is based on extraction and cleaning procedures, normally in two phase separation cycles by centrifugation [41]. The research results of Zhang et al. [74] demonstrated that the addition of sodium acetate or MgSO_4_ reduced the recovery of quinolones, sulfonamides, tetracyclines, and macrolides, even more markedly for tetracyclines, due to the formation of complex precipitation between Mg^2+^ and such substances. For identical macrolide and tetracycline chelation barriers in the extraction procedure, Jia et al. [75] opted for Na_2_SO_4_ instead of the usual MgSO_4_.

Zhao et al. [76] evaluated that the extraction efficiency of β-lactams, lincomycin, quinolones, and tetracyclines, as well as the stability of quinolones and tetracyclines, were notably affected by the addition of salts, i.e., poor recovery and accuracy. Furthermore, MgSO_4_ did not prove to be a good option for tetracyclines and quinolones, since the divalent metal ion (Mg^+2^) affects the extraction of tetracyclines, and anhydrous magnesium sulfate presents easy coagulation.

On the other hand, sodium sulfate is associated with an improvement in the percentages of recovery of polar analytes [77,78]. Sodium chloride, however, has the function of controlling the polarity of the extraction solvent with a consequent increase in its selectivity [79]. When associated with MgSO_4_, it provides better phase separation, as well as a decrease in co-extracted matrix components [80].

In an analysis of different classes of antimicrobials, anti-inflammatory, antiparasitic, and tranquilizers, totaling 105 substances in meat, fish, milk, eggs, and fat, Desmarchelier et al. [81] used a mixture of salts composed of sulfate and sodium chloride, since there are reports that magnesium sulfate induces quinolone chelation. In addition, magnesium sulfate, like sodium sulfate, has low vapor pressure and can be deposited on the mass spectrometry source and even on the analyzer, impairing the performance of the equipment. Pugajeva et al. [36] also evaluated a salting-out with the addition of NaCl and MgSO_4_ during the extraction process, when they described that this step caused reduced recoveries for antimicrobials of the quinolone and macrolide classes, non-hormonal anti-inflammatories, antiparasitics, and anticoccidials, providing recoveries of less than 50%.

Regarding the cleaning of the supernatant extract (cleanup) using d-SPE, it should be noted that the choice of the ideal sorbent should be tested by removing interfering co-extracted from the matrix in the acetonitrile extract [80], in which sorbents are used depending on the chemical nature of the analytes [82]. In this process, the addition of silica modified with C18 is associated with the removal of high levels of lipids (≥2%), promoting an even more efficient cleaning [83,84].

#### 3.2.1. Selectivity/Specificity

Based on selectivity, it becomes feasible to improve the sample preparation method, in addition to the chromatographic and MS conditions, even before the start of method validation [73].

Although the selectivity of a given method does not represent a property to be considered of an instrumental technique, but that of an analytical procedure [85], the acquisition of the exact mass of a given compound can be adopted as a selective criterion for its identification [46], an essential requirement for complex matrices or low concentration substances [86]. As far as you are concerned, HRMS selectivity is defined by the resolving power and mass accuracy of the instrument in conjunction with the user-delimited mass extraction window [87]. Instruments such as Orbitrap allow a high-resolution power (m/Δm50% > 100,000 resolving power) and mass precision (<5 ppm), which ensures correct identification of the chemical formula of pseudomolecular ions [88].

According to Kaufmann et al. [89], the use of a precursor ion and a single product ion of HRMS accurate mass (*m/z*) can also be enough for confirmation. As already described in the section on materials and methods, in this research, we chose to outline a more rigorous additional criterion, which contributes additional information in the identification and increase of selectivity/specificity by reducing the number of false–positive results. Although some analytes demonstrate similar retention times, the application of UHPLC combined with other information aimed at identifying these substances, such as MS/MS fragmentation data (at least two) [46] and an isotopological pattern [90], obtained by the Q-Exactive MS system in Full-MS mode, with an established mass resolution of 35,000 combined with a mass resolution of 17,500 FWHM in the directed AIF mode, demonstrated enough selectivity to measure an *m/z* with four decimal places, as well as meeting the identification points recommended by the European Union.

Despite how Molognoni et al. [72] report that low molecular weight substances are subject to interfering transitions in the LC-MS analysis coming from complex matrices such as meat, in our study, the HRMS provided selective method results, evidencing the suitability of this technique for the purpose qualitative screening.

#### 3.2.2. Detection Capability for Screening (CCβ)

The quality of a screening method can be evidenced by the rates of false–positive and false–negative results, depending on the agreement with the acceptability criteria established for that purpose [91]. Still, the routine use of a screening technique becomes conceivable only if the number of false–positive results is controllable to a certain extent [46]. It is noteworthy that, although the maximum rate of false positive results is not foreseen in guidelines or widely recognized in regulatory frameworks, its reduction is extremely valuable, since a high rate (>10%) can impair the applicability of the assay itself [92].

Varenina et al. [93] reported that CCβ for screening methods established by the European Union (2021/808), is the most efficient strategy to identify suspicious and negative samples in routine laboratories with a high number of samples.

When analyzing the dataset in more depth, we found that of the total number of pharmacologically active substances evaluated (n = 157), for method A, 56 (35.7%), and for method B, 53 (33.8%) substances were not identified at any level. Looking at the lowest level evaluated, corresponding to 0.50 LMR (CCβ), although using method A, only 54 analytes (34.4%) and in method B, 68 (43.3%), were fully validated according to the criteria adopted in this study for CCβ (19 or 20 positive responses), 91 (58.0%) substances were identified according to Section 4.6 in method A and 96 (61.1%) for method B, regardless of the number of responses. Of this total, 61 substances were identified in common in both methods (38.9%). When we evaluated the highest level (2.00 × LMR), for method A, 100 substances were identified (63.7%), and for method B, 102 (65.0%). Of this total, 19 or 20 positive responses were obtained, 87 substances for method A (55.4%) and 98 for method B (62.4%).

## 4. Materials and Methods

### 4.1. Scope of Veterinary Drugs

The active principles of interest were defined based on the analytes contained in the PNCRC monitoring subprogram for food of animal origin [94], in the guidelines established by the *Codex Alimentarius* [95] and European Union [96]. Based on our previous work [97], we also included the prospection of the group of antimicrobials approved for use in Brazil in bovine, porcine, and poultry animal species, but were not yet regulated.

### 4.2. Standard and Standard Solutions

Pharmacologically active substances/marker residues from eight different groups were included in the analytical scope namely: antimicrobials from different classes [aminoglycosides (10), amphenicols (3), beta-lactams (14), lincosamides (2), macrolides (9) quinolones/fluoroquinolones (10), sulfonamides (13), tetracyclines (4) and others (9)], growth promoter (3), anticoccidials (15), anti-inflammatory (16) (non-hormonal and steroidal), antiparasitic (23) and their metabolites (8), beta agonists (5), sedatives (6), and dyes (2) and their metabolites (2). Commercial analytical standards for the total of 154 substances searched were acquired from different manufacturers: Dr Ehrenstorfer (Augsburg, Germany), LGC Standards GmbH (Wesel, Germany), Toronto Research Chemicals (TRC Inc.) (North York, ON, Canada), Sigma-Aldrich (St. Louis, MO, USA) e Witega (Berlin, Germany).

Individual stock solutions were prepared by dissolving the appropriate amount of the substance in a proper solvent according to its solubility, mostly in methanol, at concentrations from 10 to 1000 mg mL^−1^ (available in Table S1, Supplementary Materials for details). The multi-compound working solutions (two) were prepared by diluting the individual stock solutions in acetonitrile in a relevant volume. All standard solutions mentioned were stored at −20 °C until use.

### 4.3. Reagents

The reagents, all analytical grade, were purchased commercially and used without further purification. Acetonitrile (ACN) and methanol (MeOH), both LC-MS grade, were obtained from JT Baker (Center Valley, PA, USA); the formic acid (98% *v*/*v*), from Panreac (Barcelona, Spain); and the sodium chloride (NaCl) (99.8% *w*/*w*), from Synth (Diadema, Brazil). HPLC grade hexane, glacial acetic acid (99.8% *v*/*v*), ethylenediaminetetraacetic acid (EDTA disodium salt) (≥99% *w*/*w*), anhydrous magnesium sulfate (MgSO_4_) (98% *w*/*w*), and anhydrous sodium sulfate (Na_2_SO_4_) (≥99% *w*/*w*) were supplied by Sigma-Aldrich (Saint Louis, MO, USA). In turn, ammonium formate (CH_5_NO_2_) (99% *w*/*w*) was received from Acros Organics (Geel, Belgium); and the dispersive phase Bondesyl octadecylsilane-C18 (40 µm), from Agilent Technologies (Santa Clara, CA, USA). Finally, the ultrapure water was generated using the Milli-Q Gradient purification system, manufactured by Millipore (conductivity <0.055 µS cm^−1^, resistivity = 18.2 MΩ cm) (Bedford, MA, USA).

### 4.4. Sample Preparation

For the experiments, a pool of ‘non-enriched’ control (or blank) samples from different origins (4 kg) was used, which were selected in a previous analysis by UHPLC-MS/MS due to the absence of compounds of interest from different cuts of bovine muscle tissues from slaughterhouses inspected by MAPA and obtained in the LFDA routine. Excess fat and visible fibrous capsules were removed, processed in a meat grinder (CAF Maquinas, Rio Claro, Brazil), homogenized, and then stored at −20 °C before analysis.

For comparative purposes, we thoroughly investigated two distinct multi-residue approaches of generic extraction and sample cleaning (here named “method A” and “method B”). Such procedures were developed by Dasenaki et al. [48] and Silva et al. [22], in which modifications were made as follows:

#### 4.4.1. Method A

A mass of 1.00 ± 0.05 g of properly chopped bovine muscle was weighed directly in a 50 mL polypropylene centrifuge tube. For the generic extraction of the analytes, 2 mL of a solution formed by 0.1% (*w*/*v*) EDTA in water with 0.1% (*v*/*v*) formic acid were added, followed by the addition of 2 mL of ACN and of 2 mL of MeOH. After each addition of the indicated solvents, the tube was vortexed (Genius 3 IKA, Staufen, Germany) for 1 min. Subsequently, the tubes were taken to the ultrasonic bath (Elma Elmasonic P, Stuttgart, Germany) for 20 min at 45 °C, with a frequency of 37 kHz. In the following, the extract was centrifuged (Thermo Scientific Heraeus Megafuge 40, Waltham, MA, USA) for 15 min at 2700× *g* and 0 °C, and the supernatant was transferred to a new 50 mL polypropylene centrifuge tube, stored at −80 °C (Ultrafreezer Indrel Scientific, Londrina, Brazil). The cleaning was completed after centrifugation for 15 min at 2700× *g* and 0 °C, with transfer to a 15 mL test tube, at which time the sample was degreased with 1 mL of hexane and vigorously stirred for 1 min in a vortex.

After discarding the top layer of hexane, the extracts were further dried at 45 ± 3 °C under air flow in a Techne DB-3 sample concentrator (Stone, UK). Finally, the residue was reconstituted with 0.5 mL of 0.01% aqueous formic acid/MeOH solution (75:25 *v*/*v*), vortexed for 30 s, and then filtered with a 0.22 μm nylon membrane filter in a 2 mL HPLC vial insert, followed by UHPLC-Q-Exactive™ Orbitrap (Thermo Scientific, Bremen, Germany) analysis (see Figure S1 in Supplementary Materials).

#### 4.4.2. Method B

A mass of 5.00 ± 0.05 g of a bovine muscle sample was weighed directly in a 50 mL polypropylene tube and then, for extraction, 10 mL of ACN acidified with 2% *v*/*v* of acetic acid was added. The mixture was vigorously shaken in a tube shaker, Vortex Genius 3 IKA (Staufen, Germany), for 1 min. After this step, 6.0 g of an extraction salt mixture composed of 4 g of Na_2_SO_4_ and 2 g of NaCl was added, followed by vortexing for 1 min and sonication for 5 min at 25 °C in an Elma Elmasonic P ultrasonic bath (Stuttgart, Germany) with a frequency of 37 kHz. This mixture was centrifuged for 10 min at 2700× *g* at 5 °C in a Thermo Scientific Heraeus Megafuge 40 centrifuge (Waltham, MA, USA). After this period, the entire supernatant was transferred to a second 50 mL polypropylene tube containing 500 mg of the dispersive mixture that consisted of 250 mg of anhydrous MgSO_4_ and 250 mg C18, and after being vortexed for 1 min, and rested for 5 min. Next, this sample was homogenized by vortexing for around 30 s and subsequently centrifuged for 10 min at 2700× *g* in a centrifuge cooled to 5 °C. In the following, the supernatant was divided into two fractions of 4 mL, transferred with a pipette to two test tubes of 15 mL, and evaporated at 45 ± 3 °C under a stream of compressed air to dryness.

Finally, to obtain the aqueous extract, the residue from tube 1 was reconstituted with 1 mL of mobile phase A, vortexed for 30 s, and filtered through a 0.22 µm nylon membrane filter (Analitica, Sao Paulo, Brazil) in a HPLC vial. In order to get the organic extract, the residue from tube 2 was solubilized with 1 mL of mobile phase B, and after stirring, it was filtered through a 0.22 µm hydrophobic polytetrafluoroethylene (PTFE) membrane filter (Analitica, Sao Paulo, Brazil). Both extracts were injected into the UHPLC-Q-Exactive^TM^ Orbitrap system (shown in Figure S2 Supplementary Materials).

### 4.5. Instrumentation

#### 4.5.1. Chromatographic Conditions

The operating configuration used here was based on those described by Dasenaki et al. [48] (method A) and Silva et al. [22] (method B), with minor adaptations as described below.

The separation of the analytes present in the extracts was performed in an Accela 1250 Pump UHPLC liquid chromatography system from Thermo Scientific (Bremen, Germany) equipped with a binary pump, vacuum degasser, oven, and autosampler. In both methods, an Acquity^TM^ UHPLC BEH C18 reserved-phase column (50 × 2.1 mm i.d., 1.7 μm particle size) was used coupled to a VanGuardAcquity^TM^ UPLC BEH C18 pre-column (5 × 2.1 mm i.d., 1.7 mm), both from Waters (Wexford, Ireland).

For method A, the column and precolumn were operated at 30 °C and the autosampler was maintained at 15 °C. The mobile phases consisted of 0.01% formic acid in water (A) and methanol (B). The gradient program applied was a linear ramp from 95% A and 5% B to 0% A and 100% B in 7.0 min, and from 7.0 to 10.0 min it was held constant at 100% B; decreasing linearly to 5% B and 95% A in 0.1 min, maintaining the initial condition up to 17.0 min. The flow was 0.1 mL min^−1^ and the injection volume was 5 µL.

For method B, two conditions for the separation of analytes were applied to each extract of the sample, using a flow rate of 0.4 mL min^−1^ and an injection volume of 10 µL. For the aqueous extract, the column and autosampler temperatures were 35 and 15 °C, respectively. The mobile phases consisted of 5 mmol L^−1^ ammonium formate + 0.1% formic acid in water (A) and 0.1% formic acid in water/ACN (5:95, *v*/*v*) (B). The elution gradient was as follows: 100% A to 80% A in 8.0 min, 80% A to 5% A from 8.0 to 14.0 min, with hold up to 18.0 min; from 5% A to 100% from 18.0 to 20.0 min. For the organic extract, the column was operated at a temperature of 30 °C, while the automatic sample was at 15 °C. The mobile phases were composed of ammonium formiate 0.2 mol L^−1^ + formic acid 0.1% in water (A) and formic acid 0.1% in water/ACN (5:95, *v*/*v*) (B). Gradient elution was 100% A up to 7.5 min; 100% A up to 2% A in 2.5 min, with maintenance up to 17.5 min, and re-establishment of the initial gradient with 100% A up to 20 min (Table S2 in Supplementary Materials).

#### 4.5.2. Q-Orbitrap High-Resolution Mass Spectrometry Parameters

The high-resolution mass spectrometer (HRMS) used to identify the substances was the Q-Exactive^TM^ hybrid quadrupole-Orbitrap^TM^ (Thermo Scientific, Bremen, Germany), equipped with a heated electrospray ionization source (HESI-II). The analyses were performed by two acquisition methods: the MS full scan acquisition mode which operates by switching between positive and negative ionization modes, for data acquisition at a resolution of 35,000 full width at half maximum (FWHM) (MS^1^) in the range from 65 to 975 *m/z*, and full scan mode with all ion fragmentation (AIF) or MS^2^ experiments, operated at a resolution power of 17,500 FWHM in order to increase the number of detected signals for each product ion, using a three-step scaled normalized collision energy (NCE) for target analytes at values on 10, 35, and 80 eV, in high energy collisional dissociation (HCD) to ensure complete fragmentation of precursor ions. In both cases, the automatic gain control (AGC) representing the C-trap was 3 × 10^6^ ions for the maximum injection time (IT) of 100 ms and 1.0 microscans for the final mass spectra acquisition. The following source conditions were employed to assist the ionization process: electrospray voltage of the HESI interface was adjusted to 3.9 and 2.9 kV in positive and negative ionization modes, respectively; there was a capillary temperature of 350 °C; lens voltage S was set to 50 (arbitrary unit—arb); and a sheath and auxiliary gas flow rate (N_2_ 0.545 mbar) of 40 arb and 15 arb, respectively (Table S3 in Supplementary Materials).

A study of the adequacy of procedures was performed, as well as the optimization of method conditions for detection by HESI-Orbitrap^TM^ mass spectrometry (MS). A database of substances, which contained information on the molecular formula, the exact mass of precursor ions, and the corresponding potential adducts (including a selection of protonated ions [M]^+^, [M+H]^+^, [M+Na]^+^, [M+H_2_O+H]^+^, [M-H_2_O+H]^+^, [M+H+CH_3_OH]^+^, [(M+H_2_CO)+H]^+^, [(M+H_2_-CO)+H]^+^, [M+NH_4_]^+^, [M+Ca]^+^, [M+H_3_O]^+^, deprotonated ions [M-H]^−^, double charged ions [M+2H]^2+^, [M+H_2_O+2H]^2+^, or tri-charged ions [M+3H]^3+^ in the positive operation mode, with up to five MS/MS of the most abundant fragment ions being defined considering the theoretical *m/z* of the substance, in addition to the respective chromatographic retention times. Furthermore, the structures and details of the physicochemical properties of each analyte were presented, such as partition coefficient (log *p*), acid/base dissociation constants (pKa), and intrinsic solubility (data in Supplementary Materials, Table S4) obtained after direct infusion of individual standard solutions into the MS system with ionization by electrospray in the positive and negative modes (HESI^+^ e HESI^−^) to select the most intense ions by means of fragmentation energy scans, in a solvent at a concentration of 500 μg L^−1^ in MeOH:H_2_O 0.1% (*v*/*v*) of formic acid. Retention times were evaluated by injecting a fortified extract into the matrix in the UHPLC-Q-Orbitrap^TM^ at the same concentration as the other parameters.

The acquisition and processing of raw data were performed using the software Xcalibur Analysis 3.0 (Thermo Fisher Scientific Inc., Waltham, MA, USA) for the creation of methods and for the execution of the samples, with the exact mass of the substances calculated using Qualbrowser.

### 4.6. Qualitative Screening Validation Study

The suitability of the proposed method to its scope of application followed the validation protocol in accordance with Regulation 2021/808 [98] and the technical instructions of the guidelines for the validation of screening methods for residues of veterinary medicines [99], which covers the evaluation of the following figures of merit: selectivity/specificity against interference and detection capacity (CCβ).

The experimental design was carried out in part as previously described by Gondim et al. [100]. At the beginning of each extraction procedure, on the same day, the pool of all analytes was added to the blank sample at six concentration levels, corresponding to 0.50, 0.75, 1.0, 1.50, 1.75, and 2.0 times the maximum reference limit plus blank, in 10 true replicas (n = 10) for each level. Spiked samples were left for at least 30 min to rest and this same series was repeated in another analytical batch on a different day, totalizing 20 enriched samples per method.

Such limits were based on values of MRL and minimum required performance level (MRPL), as established by Brazilian regulation National Health Surveillance Agency (ANVISA) [101] for bovine muscle; when not available, limits recommended by the *Codex Alimentarius* [95], by European legislation [96], Food and Drug Administration (FDA) from the United States [102], in Japan [103], in Australia [104] and in the PNCRC [94] in this order of reference. With regards to drugs that did not have MRLs, a validation level (VL) was established based on the characteristics of the referred drug as well as other drugs of the same class. In some specific cases, the existing MRL for porcine or poultry muscle was used. Finally, if it was not possible to apply the previous criteria, the limit adopted was the concentration of 10 μg kg^−1^ (see Table S1 in Supplementary Materials).

Thus, in this study, the analyte identification criteria were carefully defined as follows: expected retention time (± 0.5 min deviation tolerance), the exact mass of the precursor ion (<±0.5 of the mass range), the minimum of two product ions with mass accuracy *(m/z)* ≤ 5 ppm, analyte peaks of the precursor ion and product(s) in the chromatograms of extracted ions, the latter two that must completely overlap (Fully overlap), and the isotopological standard (minimum 2) (Figures S3 and S4 in Supplementary Materials). In this sense, as the proposed method presented a score corresponding to 7,5 identification points (IPs), the analytes were correctly identified [98].

#### 4.6.1. Selectivity/Specificity

Selectivity, which is nothing more than the property of a method to distinguish a certain analyte and other closely associated substances, was estimated considering the broad scope that included metabolites of the residue of interest, intrinsic constituents of the matrix, or other potential interfering substances of the sample, as those that have an inhibitory or potentiating effect on the detection of analytes by the methodology. The study of interfering potentials was carried out by evaluating the chromatograms concerning the existence of peaks or significant interfering signals in the retention times of the selected analytes, that is, each substance was monitored by the method in 20 blank samples of the matrix [98].

#### 4.6.2. Detection Capacity (CCβ)

For screening, the detectability or CCβ is defined as the lowest concentration of a given analyte that can be detected in a sample with a probability of error β [99]. The β error is understood as the probability that a sample is truly non-compliant, even if a compatible measurement is achieved [98]. It should be noted that the promotion of a reliable basis for this determination is obtained from a rate of false conforming results of up to 5 % (β error) [98,99]. As the authorized pharmacologically active substances, the CCβ needs to be less than or equal to the regulatory limit. In this research, the CCβ was defined at the validation level of ½ MRL [99]. This means that, by evaluating 20 samples, at least 19 must have the identification of the analyte, and only one (5%) that is not detectable is allowed.

## 5. Conclusions

Even with the existence of a culture of the relegation of screening methods [105], we demonstrated that the two methods for the analysis of veterinary drugs in bovine muscle based on HESI with an Orbitrap analyzer presented high yield and met qualitative validation performance criteria, which is still unprecedented. In view of the multi-analyte approach, with special attention to antimicrobials (e.g., marketed but not monitored in Brazil), such methods have shown characteristics such as savings in time, labor, and cost per sample, in addition to contributions related to green chemistry, which are very useful for the solution of complex questions about the chemical safety of food. Therefore, we highlight the strengths and weaknesses of each methodology and suggest method B as the best screening option in terms of the number of analytes (n = 68), which may represent an impulse for even more efficient routine analyses in official laboratories, approaching the “gold” standard.

In fact, our results shed light on a fruitful pathway toward future studies related to expanding the scope to other widely consumed meat matrices (e.g., porcine and poultry species). We also encourage the evaluation of the applicability of these methods in real samples from different slaughterhouses and Brazilian states. Another potential to be explored is to carry out a study of the occurrence and retrospective analysis of ionizable known and unknown substances at the source [106], which will make it possible to understand the food as a whole and should encourage new discoveries, whether for additional compounds or for reprocessing.

Finally, this mapping of the substances identified provides valuable evidence for the strategic direction of more assertive public policies, such as the exclusion of unnecessary active pharmaceutical ingredients and the inclusion of those registered, mainly antimicrobials, in addition to comprehensively meeting the demands that are on the horizon.

## Figures and Tables

**Table 1:** General summary of the two analytical procedures and their results.

Parameter	Result		Criterion	Assessment	
	Method A	Method B		Method A	Method B
Description	Solid-liquid extraction using a mixture of solvents, assisted by ultrasound; cleanup in low temperature and hexane; UHPLC-Q-Orbitrap analysis	QuEChERS method -Acetonitrile extraction using a mixture of salts (d-SPE) and ultrasound-assisted; cleanup and centrifugal separation; UHPLC-Q-Orbitrap analysis	Less complexity possible	Less Steps	More steps
Technique complexity/time consumption	More time	Less time	Shortest possible time per sample		
Sample weight	1 g	5 g	Smallest possible	Lower consumption	Higher consumption
Solvent consumption	2 mL ACN; 2 mL MeOH; 1 mL hexane	10 mL of ACN acidified with 2% *v*/*v* of acetic acid	Less possible	Lower consumption	Higher consumption
Consumption of solutions	2 mL 0.1% (*w*/*v*) EDTA in water with 0.1% (*v*/*v*) formic acid; 0.5 mL of 0.01% aqueous formic acid/MeOH solution (75:25 *v*/*v*)	Aqueous extract: 1 mL mobile phase A; Organic extract: 1 mL mobile phase B	Smallest possible	Higher consumption	Lower consumption
Amount of solvents	None	Na_2_SO_4_ (4 g); NaCl (2 g); MgSO_4_ (250 mg); C18 (250 mg)	Smallest possible	No consumption	Higher consumption

**Table 2:** Validation results for the multiclass screening of veterinary drugs involving selectivity and CCβ values, evaluated at different proportions of the maximum residue limit per analyte, in 20 samples for each method examined.

Group/Class— Pharmacologically Active Substance	MRL in Bovine Muscle (µg kg^−1^)	CCβ (0.50 MRL) (µg kg^−1^)	Method A							Method B						
			Validation Parameter							Validation Parameter						
			Selectivity			CCβ				Selectivity		CCβ				
			Ratio of the Maximum Limit (MRL)							Ratio of the Maximum Limit (MRL)						
			0.00 (Blank Samples)	0.50	0.75	1.00	1.50	1.75	2.00	0.00 (Blank Samples)	0.50	0.75	1.00	1.50	1.75	2.00
Antimicrobials/ Aminoglycosides																
Amikacin	500	250														
Apramycin	1000	500														
Dihydrostreptomycin	600	300														
Spectinomycin	500	250														
Streptomycin	500	250														
Gentamicin (C1, C1a, C2-C2a)	100	50														
Hygromycin B	500	250														
Kanamycin A	100	50														
Neomycin B	500	250														
Tobramycin	500	250														
Antimicrobials/ Amphenicols																
Chloramphenicol	0.3	0.15								20	8	9	11	13	18	20
Florfenicol	200	100	20	20	20	20	20	20	20	20	20	20	20	20	20	20
Thiamphenicol	50	25	20	12	15	16	17	20	20	20	20	20	20	20	20	20
Antimicrobials/ Betalactams																
Amoxicillin	50	25														
Ampicillin	50	25	20	2	14	16	20	20	20	20	0	5	11	13	17	20
Cephalexin	200	100	20	9	15	18	20	20	20	20	0	0	7	15	17	20
Cefalonium	10	5														
Cephapirin	50	25	20	0	0	0	2	18	16	20	4	13	18	19	20	20
Cefazolin	50	25								20	0	0	6	14	18	18
Cefoperazone	50	25								20	10	18	20	20	20	20
Cefquinome	50	25	20	0	0	2	4	8	4	20	7	12	16	19	20	20
Cloxacillin	300	150	20	2	7	13	19	18	18	20	20	20	20	20	20	20
Dicloxacillin	300	150	20	18	18	16	20	20	20	20	20	18	20	20	20	20
Nafcillin	300	150	20	19	20	20	20	20	20							
Oxacillin	300	150	20	18	18	20	20	20	20	20	20	20	20	20	20	20
Benzylpenicillin	50	25	20	20	20	20	20	20	20	20	19	20	20	20	20	20
Phenoxymethylpenicillin/Phenoxymethyl V penicillin	25	12.5	20	4	10	13	17	20	20	20	5	9	11	17	20	20
Antimicrobials/ Lincosamides																
Clindamycin	50	25	20	13	20	19	20	19	20	20	20	20	20	20	20	20
Lincomycin	100	50	20	0	2	8	20	20	20							
Antimicrobials/ Macrolides																
Azithromycin	50	25								20	20	20	20	20	20	20
Erythromycin A	200	100	20	10	16	20	20	20	20							
Spiramycin I	200	100								20	20	20	20	20	20	20
Tilmicosin	100	50	20	6	14	15	16	18	18	20	18	18	19	19	20	20
Tylosin A	100	50	20	0	3	4	7	13	14	20	19	19	20	20	20	20
Antimicrobials/ Quinolones-Fluoroquinolones																
Nalidixic acid	20	10	20	20	20	18	20	20	20	20	20	20	20	20	20	20
Oxolinic acid	100	50	20	20	20	18	20	20	20							
Ciprofloxacin	100	50	20	18	20	20	20	20	20	20	20	20	18	20	20	20
Danofloxacin	200	100	20	17	18	20	20	20	20	20	20	20	20	20	20	20
Difloxacin	400	200	20	20	16	16	20	18	20	20	20	20	20	20	20	20
Enrofloxacin	100	50	20	20	20	20	20	20	20	20	20	20	20	20	20	20
Flumequine	500	250	20	20	20	20	20	20	20	20	20	20	20	20	20	20
Norfloxacin	20	10	20	20	20	20	20	20	18	20	20	20	20	20	20	20
Sarafloxacin	10	5	20	9	13	15	19	20	20	20	20	20	20	20	20	20
Antimicrobials/ Sulphonamides																
Sulfachlorpyridazine	100	50	20	20	20	20	20	20	20	20	20	20	20	20	20	20
Sulfadiazine	100	50	20	17	18	20	20	20	19							
Sulfadimethoxine	100	50	20	19	20	20	20	20	20	20	20	20	20	20	20	20
Sulfadoxine	100	50	20	20	20	20	20	20	20	20	20	20	20	20	20	20
Sulfisoxazole/ Sulfafurazole	100	50	20	20	20	20	20	20	20	20	20	20	20	20	20	20
Sulfamerazine	100	50	20	19	20	20	20	20	20	20	20	20	20	20	20	20
Sulfamethazine/Sulfadimidine	100	50	20	20	20	20	20	20	20	20	20	20	20	20	20	20
Sulfathiazole	100	50	20	20	20	20	20	20	20	20	20	20	20	20	20	20
Sulfamethoxazole	100	50	20	20	20	20	20	20	20	20	0	2	9	12	18	20
Sulfaquinoxaline	100	50	20	20	20	20	20	20	20	20	20	20	20	20	20	20
Antimicrobials/ Tetracyclines																
Chlortetracycline	200	100	20	18	20	20	20	19	20	20	20	20	20	20	20	20
Doxycycline	100	50	20	20	20	20	20	20	20							
Oxytetracycline	200	100	20	18	19	20	20	19	20	20	7	8	17	20	20	20
Tetracycline	200	100	20	20	20	20	20	20	20	20	6	13	20	20	20	20
Antimicrobials/ Others																
Bromhexine	10	5	20	7	9	16	17	30	20	20	3	9	18	19	20	20
Dapsone	10	5	20	0	0	2	5	9	6							
Rifampicin	10	5	20	19	20	20	20	20	20	20	20	20	20	20	20	20
Tiamulin	100	50	20	20	20	20	20	20	20	20	20	20	20	20	20	20
Antimicrobials Prospect/ Quinolones-Fluoroquinolones																
Marbofloxacin	150	75	20	16	16	20	20	20	20	20	18	19	20	20	20	20
Antimicrobials Prospect/ Macrolides																
Josamycin/Leucomycin A_3_	100	50								20	20	20	20	20	20	20
Leucomycin/Leucomycin A_5_	100	50	20	2	2	2	8	12	14							
Tildipirosin	400	200	20	19	19	19	20	20	20	20	18	18	19	20	20	20
Tulathromycin A	300	150	20	15	18	18	19	19	20	20	20	20	20	20	20	20
Antimicrobials Prospect/Sulfonamides																
Phthalylsulfathiazole	100	50	20	20	20	20	20	20	20	20	20	20	20	20	20	20
Sulfacetamide/*N*-Sulfanilylacetamide	100	50	20	9	16	17	19	20	20							
Sulfamethoxypyridazine	100	50	20	20	20	20	20	20	20	20	20	20	20	20	20	20
Antimicrobials Prospect/Others																
Diminazene	500	250	20	20	16	16	20	18	20	20	20	20	20	20	20	20
Fosfomycin	500	250														
Isoniazid	10	5	20	20	20	20	20	20	20							
Novobiocin	1000	500														
Rifaximin	10	5	20	18	20	20	20	20	20							
Antibiotic Growth Promoters																
Dichloroisoeverninic acid	800	400	20	17	20	20	20	20	20	20	17	20	20	20	20	20
Halquinol/Chlorhydroxyquinoline(7-Chloro-8-quinolinol)	40	20								20	15	16	15	18	19	20
Halquinol/Chlorhydroxyquinoline (5,7-Dichloro-8-quinolinol/Chloroxine)	40	20								20	18	19	19	20	20	20
Halquinol/Chlorhydroxyquinoline (5-Chloro-8-hydroxyquinoline)	40	20								20	14	16	16	18	20	20
Virginiamycin M_1_	100	50	20	20	20	20	20	20	20	20	18	18	18	19	20	20
Anticoccidials																
Amprolium	500	250								20	20	20	20	20	20	20
Clopidol	200	100	20	20	9	17	18	20	20							
Diaveridine	50	25	20	0	5	11	14	20	20							
Decoquinate	1000	500	20	20	20	20	20	20	20	20	20	20	20	20	20	20
Diclazuril	50	25	20	19	20	20	20	20	20	20	20	20	20	20	20	20
Ethopabate	500	250	20	18	18	20	20	20	20	20	7	11	12	13	17	18
Lasalocid A	10	5								20	18	18	18	19	20	20
Maduramicin	30	15	20							20	20	20	20	20	20	20
Monensin A	10	5	20							20	9	14	15	16	19	20
Narasin A	15	7.5	20													
4,4′-Dinitrocarbanilide – DNC	4000	2000	20	12	20	16	20	20	20	20						
Robenidine	200	100														
Salinomycin	20	10	20	0	0	0	4	5	9	20	19	20	20	20	20	20
Toltrazuril	100	50	20	13	15	16	16	17	17							
Trimethoprim	50	25								20	20	20	20	20	20	20
Anti-inflammatories/ Steroidal																
Prednisolone	4	2								20	20	20	20	20	20	20
Prednisone	4	2														
Anti-inflammatories Prospect/Steroidal																
Isoflupredone acetate/9-Fluoroprednisolone acetate	10	5														
Flumetasone	10	5								20	10	17	18	20	20	20
Anti-Inflammatory/ Non-Steroidal																
Mefenamic acid	20	10	20	18	20	20	20	20	20	20	16	18	18	19	20	20
Tolfenamic acid	50	25	20	19	20	20	20	20	20	20	9	10	17	18	20	20
Carprofen	500	250	20	20	20	20	20	20	20							
Ketoprofen	50	25	20	0	2	2	4	6	12	20	20	20	20	20	20	20
Diclofenac	5	2,5								20	18	18	19	20	20	20
Flunixin	20	10	20	20	20	20	20	20	20	20	10	12	18	17	20	20
Indomethacin	20	10								20	20	20	20	20	20	20
Meloxicam	20	10	20	20	20	20	20	20	20	20	0	0	2	4	8	13
Naproxen	20	10								20	20	20	20	20	20	20
Nimesulide	20	10	20	20	20	20	20	20	20							
Piroxycam	20	10	20	17	19	20	20	20	20	20	20	20	20	20	20	20
Propyphenazone	20	10	20	18	19	20	20	20	20							
Antiparasitic/ Avermectins																
Avermectin B_1a_/Abamectin B_1a_	20	10								20	20	20	20	20	20	20
Doramectin	10	5														
Emamectin B_1a_	2	1														
Eprinomectin B_1a_	100	50														
Ivermectin B_1a_/22,23-Dihydroavermectin B_1_	30	15														
Moxidectin	20	10								20	13	15	16	19	20	20
Antiparasitic/ Benzimidazoles																
Albendazole	100	50	20	20	20	20	20	20	20							
Albendazole sulfone	100	50	20	20	20	20	20	20	20							
Albendazole sulfoxide	100	50	20	20	20	20	20	20	20							
Closantel	1000	500	20	19	19	19	19	19	20	20	20	20	20	20	20	20
Febantel	100	50	20	20	20	20	20	20	20	20	20	20	20	20	20	20
Fenbendazole	100	50	20	20	20	20	20	20	20	20	19	20	20	20	20	20
Fenbendazole sulfone	100	50	20	20	20	20	20	20	20	20	20	20	20	20	20	20
Flubendazole	20	10	20	20	20	20	20	20	20	20	20	20	20	20	20	20
2-Aminoflubendazole	20	10	20	20	20	20	20	20	20	20	20	20	20	20	20	20
Levamisole	10	5	20	6	6	16	19	19	19	20	19	19	20	20	20	20
Mebendazole	20	10	20	20	19	20	20	20	20	20	20	20	20	20	20	20
Oxibendazole	100	50	20	20	20	20	20	20	20	20	20	20	20	20	20	20
Oxfendazole	100	50	20	20	20	20	20	20	20	20	20	20	20	20	20	20
Thiabendazole	100	50	20	19	20	20	20	20	20	20	20	20	20	20	20	20
Triclabendazole	250	125								20	20	20	20	20	20	20
Antiparasitic/ Phenylpyrazoles																
Fipronil	500	250	20	20	20	20	20	20	20	20	20	20	20	20	20	20
Fipronil sulfone	500	250								20	20	20	20	20	20	20
Antiparasitic/ Nitroimidazoles																
Dimetridazole	10	5								20	20	20	20	20	20	20
Hydroxydimetridazole/HMMNI	10	5								20	19	19	20	20	20	20
Ipronidazole	3	1.5	20	0	0	0	0	2	6	20	13	15	16	19	20	20
Hydroxy Ipronidazole	3	1.5	20	2	2	10	18	20	18							
Metronidazole	10	5														
Hydroxymetronidazole	10	5								20	0	4	9	11	12	16
Ronidazole/1-Methyl-2-carbamoyloxymethyl-5-nitroimidazole	10	5								20	3	7	15	19	20	20
Antiparasitic/ Isoquinoline-pyrazines																
Praziquantel	300	150	20	18	18	19	20	20	20							
Beta-Agonists																
Cimaterol	10	5								20	19	20	20	20	20	20
Clenbuterol	0.2	0.1								20	20	20	20	20	20	20
Ractopamine	10	5								20	20	20	20	20	20	20
Salbutamol	10	5	20	9	13	19	20	20	20							
Zilpaterol	0.5	0.25								20	4	8	10	15	17	19
Sedatives																
Acepromazine	10	5	20	11	13	20	20	20	19	20						
Azaperol	60	30	20	20	20	20	20	20	20							
Azaperone	60	30	20	20	20	20	20	20	20							
Carazolol	5	2.5	20	11	15	20	20	20	20	20	20	20	20	20	20	20
Chlorpromazine	10	5	20	20	20	20	20	20	20	20	20	20	20	20	20	20
Prospecting sedatives																
Xylazine	20	10	20	11	14	15	20	20	20							
Corants																
Gentian violet/Crystal violet	10	5	20	20	20	20	20	20	20							
Leucocrystal Violet	10	5														
Malachite green	10	5	20	18	14	14	20	20	20	20	20	20	20	20	20	20
Leucomalachite green	10	5														

## Data Availability

All data are contained within the article.

## Supplementary Materials

The following supporting information can be downloaded at: https://www.mdpi.com/article/10.3390/molecules28104150/s1, Figure S1: General flowchart of the sample preparation procedure according to method A; Figure S2: General flowchart of the sample preparation procedure according to method B; Figure S3: Analyte identification criteria; Figure S4: Examples extracted mass traces of a spiked bovine muscle tissue at 2 MRL according to the established criteria for analyte identification; Table S1: Concentration of the standard solution, maximum residue limit (MRL), minimum required performance level (MRPL) in bovine muscle; Table S2: Chromatographic conditions of the different methods evaluated for the Accela 1,250 Pump UHPLC system; Table S3: Mass spectrometer conditions of different methods evaluated for the Q-Exactive Orbitrap HRMS system; Table S4: Physicochemical properties of veterinary drugs and the main conditions determined by Q-Exactive Orbitrap HRMS; Table S5: Retention time of veterinary drugs and their metabolites evaluated by Q-Exactive Orbitrap HRMS [107,108,109,110,111,112].

## Sample Availability

Samples of the compounds are available from the authors.

## References

[1] Confederação da Agricultura e Pecuária do Brasil (2022). PIB do Agronegócio Cresceu Abaixo das pr#ojeções.

[2] Brasil. Ministério da Agricultura, Pecuária e Abastecimento (2022). Indicadores Gerais AGROSTAT.

[3] United States of America. Department of Agriculture (2022). Graphical Query: Stats by Country.

[4] Dohlman E., Hansen J., Boussios D. (2022). USDA Agricultural Projections to 2031.

[5] Mulchandani R., Wang Y., Gilbert M., Van Boeckel T.P. (2023). Global trends in antimicrobial use in food animals from 2017 to 2030. PLOS Glob. Public Health.

[6] U.S. Department of Agriculture (2019). Beef, Short Loin, Porterhouse Steak, Separable Lean Only, Trimmed to 1/8" Fat, Select, Raw.

[7] European Comission Regulation (2009). (EC) No 470/2009 of the European Parliament and of the Council of 6 May 2009 laying down Community procedures for the establishment of residue limits of pharmacologically active substances in foodstuffs of animal origin, repealing Council Regulation (EEC) No 2377/90 and amending Directive 2001/82/EC of the European Parliament and of the Council and Regulation (EC) No 726/2004 of the European Parliament and of the Council. Off. J. Eur. Union.

[8] Food and Agriculture Organization (2019). Codex Alimentarius Commission: Procedural Manual.

[9] Kleinubing N.R., Ramires T., Würfel S.F.R., Haubert L., Scheik L.K., Kremer F.S., Lopes G.V., Silva W.P. (2021). Antimicrobial resistance genes and plasmids in *Campylobacter jejuni* from broiler production chain in Southern Brazil. LWT.

[10] Martins B.T.F., Meirelles J.L., Omori W.P., Oliveira R.R., Yamatogi R.S., Call D.R., Nero L.A. (2022). Comparative genomics and antibiotic resistance of *Yersinia enterocolitica* obtained from a pork production chain and human clinical cases in Brazil. Food Res. Int..

[11] Dewdney J.M., Maes L., Raynaud J.P., Blanc F., Scheid J.P., Jackson T., Lens S., Verschueren C. (1991). Risk assessment of antibiotic residues of β-lactams and macrolides in food products with regard to their immuno-allergic potential. Food Chem. Toxicol..

[12] Molina J.D.A., Vargas R.H.L., Gutierrez J.A.B., Gallo-Ortiz A., Duarte-Correa Y. (2021). Residues of veterinary drugs and heavy metals in bovine meat from Urabá (Antioquia, Colombia), a promising step forward towards international commercialization. Vet. Anim. Sci..

[13] Saraithong W. (2018). Trade restriction rationale for food safety implementation: Evidence from Southeast Asian Countries. Cogent Econ. Finan..

[14] Wei G.-X., Yuang J.-K., Yang J. (2012). Honey safety standards and its impacts on China’s honey export. J. Integr. Agric..

[15] Barrios R.E., Khuntia H.K., Bartelt-Hunt S.L., Gilley J.E., Schmidt A.M., Snow D.D., Li X. (2020). Fate and transport of antibiotics and antibiotic resistance genes in runoff and soil as affected by the timing of swine manure slurry application. Sci. Total Environ..

[16] Brasil, Ministério da Agricultura (1979). Portaria nº 86, de 26 de Janeiro de 1979. Aprova o Programa Nacional de Controle de Resíduos Biológicos em Carnes, a Ser Executado no Âmbito da Secretaria Nacional de Defesa Agropecuária. Diário Oficial da União.

[17] Brasil, Ministério da Agricultura, Pecuária e Abastecimento (2021). Resultados do Plano Nacional de Controle de Resíduos e Contaminantes—PNCRC 2021.

[18] Mauricio A.Q., Lins E.S., Alvarenga M.B. (2009). A national residue control plan from the analytical perspective: The Brazilian case. Anal. Chim. Acta.

[19] Barreto F., Ribeiro C., Hoff R.B., Costa T.D. (2016). Determination of chloramphenicol, thiamphenicol, florfenicol and florfenicol amine in poultry, swine, bovine and fish by liquidchromatography-tandem mass spectrometry. J. Chromatogr. A.

[20] Jank L., Martins M.T., Arsand J.B., Motta T.M.C., Hoff R.B., Barreto F., Pizzolato T.M. (2015). High-throughput method for macrolides and lincosamides antibiotics residues analysis in milk and muscle using a simple liquid–liquid extraction technique and liquid chromatography–electrospray–tandem mass spectrometry analysis (LC–MS/MS). Talanta.

[21] Pastore V.A.A., Santos F.A., Lana M.A.G., Silva G.R., Figueiredo T.C., Assis D.C.S., Cançado S.V. (2022). Development and validation of a multiresidue method for the determination of macrocyclic lactones, monensin, and fipronil in bovine liver by UHPLC-MS/MS using a QuEChERS extraction. Food Anal. Methods.

[22] Silva G.R., Lima J.A., Souza L.F., Santos F.A., Lana M.A.G., Assis D.C.S., Cançado S.V. (2017). Multiresidue method for identification and quantification of avermectins, benzimidazoles and nitroimidazoles residues in bovine muscle tissue by ultra-high performance liquid chromatography tandem mass spectrometry (UHPLC-MS/MS) using a QuEChERS approach. Talanta.

[23] Martins M.T., Melo J., Barreto F., Hoff R.B., Jank L., Bittencourt M.S., Arsand J.B., Schapoval E.E.S. (2014). A simple, fast and cheap non-SPE screening method for antibacterial residue analysis in milk and liver using liquid chromatography–tandem mass spectrometry. Talanta.

[24] Kaufmann A. (2012). The current role of high-resolution mass spectrometry in food analysis. Anal. Bioanal. Chem..

[25] Kaufmann A., Arrizabalaga-Larrañaga A., Blokland M.H., Sterk S.S. (2023). Potential and limitation of retrospective HRMS based data analysis: “Have meat-producing animals been exposed to illegal growth promotors such as SARMs?. Food Control.

[26] You Y., Proctor R.M., Guo K., Li X., Xue E., Guan F., Robinson M.A. (2021). Use of high resolution/accurate mass full scan/data-dependent acquisition for targeted/non-targeted screening in equine doping control. Anal. Methods.

[27] Jongedijk E., Fifeik M., Arrizabalaga-Larrañaga A., Polzer J., Blokland M., Sterk S. (2023). Use of high-resolution mass spectrometry for veterinary drug multi-residue analysis. Food Control.

[28] Spisso B.F., Pereira M.U., Ferreira R.G., Meyers R.A. (2022). Methods of analysis for the determination of veterinary drugs in food: Focus on antimicrobials. Encyclopedia of Analytical Chemistry: Applications, Theory and Instrumentation.

[29] Hepperle J., Dörk D., Barth A., Taşdelen B., Anastassiades M. (2015). Studies to improve the extraction yields of incurred pesticide residues from crops using the QuEChERS method. J. AOAC Int..

[30] Dasenaki M.E., Michali C.S., Thomaidis N.S. (2016). Analysis of 76 veterinary pharmaceuticals from 13 classes including aminoglycosides in bovine muscle by hydrophilic interaction liquid chromatography–tandem mass spectrometry. J. Chromatogr. A.

[31] Gosetti F., Mazzucco E., Zampieri D., Gennaro M.C. (2010). Signal suppression/enhancement in high-performance liquid chromatography tandem mass spectrometry. J. Chromatogr. A.

[32] Saluti G., Diamanti I., Giusepponi D., Pucciarini L., Rossi R., Moretti S., Sardella R., Galarinia R. (2018). Simultaneous determination of aminoglycosides and colistins in food. Food Chem..

[33] Wang H., Tian H., Lian-feng Ai L.-F., Liang S.-X. (2023). Screening and quantification of 146 veterinary drug residues in beef and chicken using QuEChERS combined with high performance liquid chromatography-quadrupole orbitrap mass spectrometry. Food Chem..

[34] Sardela V.F., Martucci M.E.P., Araújo A.L.D., Leal E.C., Oliveira D.S., Carneiro G.R.A., Deventer K., Van Eenoo P., Pereira H.M.G., Aquino Neto F.R. (2018). Comprehensive analysis by liquid chromatography Q-Orbitrap mass spectrometry: Fast screening of peptides and organic molecules. J. Mass Spectrom..

[35] Moretti S., Cavanna D., Lambertini F., Catellani D., Sammarco G., Barola C., Paoletti F., Saluti G., Galarini R., Suman M. (2020). Practical approach to develop a multi-group screening method for detection of mycotoxins, pesticides and veterinary drugs in food. J. Mass. Spectrom..

[36] Pugajeva I., Ikkere L.E., Judjallo E., Bartkevics V. (2019). Determination of residues and metabolites of more than 140 pharmacologically active substances in meat by liquid chromatography coupled to high resolution Orbitrap mass spectrometry. J. Pharm. Biomed. Anal..

[37] Uczay F., Bandeira N.M.G., Floriano L., Prestes O.D., Adaime M.B., Zanella R. (2021). Determination of avermectins residues in soybean, bean, and maize using a QuEChERS-based method and ultra-high-performance liquid chromatography coupled to tandem mass spectrometry. Separations.

[38] Moscou I.C., Dasenaki M.E., Thomaidis N.S. (2019). Ionization study and simultaneous determination of avermectins and milbemycines in fish tissue by LC-ESI-MS/MS. J. Chromatogr. B Analyt. Technol. Biomed. Life Sci..

[39] Sismotto M., Paschoal J.A.R., Teles J.A., Rezende R.A.E., Reyes F.G.R. (2014). A simple liquid chromatography coupled to quadrupole time of flight mass spectrometry method for macrolide determination in tilapia fillets. J. Food Compos. Anal..

[40] Grunwald L., Petz M. (2003). Food processing effects on residues: Penicillins in milk and yoghurt. Anal. Chim. Acta.

[41] Qi P., Zhou Q.-Q., Lin Z.-H., Liu J., Cai W.-Y., Mao X.-W., Jiang J.J. (2021). Qualitative screening and quantitative determination of multiclass water-soluble synthetic dyes in foodstuffs by liquid chromatography coupled to quadrupole Orbitrap mass spectrometry. Food Chem..

[42] Campanholi K.S., Ana Beatriz Zanqui A.B., Morais F.A.P., Jaski J.M., Gonçalves R.S., Silva Junior R.C., Cardozo-Filho L., Caetano W. (2022). Obtaining phytotherapeutic chlorophyll extracts using pressurized liquid technology. J. Supercrit. Fluids.

[43] Gałuszka A., Migaszewski Z., Namieśnik J. (2013). The 12 principles of green analytical chemistry and the SIGNIFICANCE mnemonic of green analytical practices. TrAC.

[44] Cifuentes A. (2012). Food analysis: Present, future, and foodomics. Int. Sch. Res. Netw..

[45] García-Cañas V., Simó C., Herrero M., Ibáñez E., Cifuentes A. (2012). Present and future challenges in food analysis: Foodomics. Anal. Chem..

[46] Kaufmann A., Butcher P., Maden K., Walker S., Widmer M. (2017). Using in silico fragmentation to improve routine residue screening in complex matrices. J. Am. Soc. Mass Spectrom..

[47] Berendsen B.J.A., Stolker L.A.M., Nielen M.W.F. (2013). Selectivity in the sample preparation for the analysis of drug residues in products of animal origin using LC-MS. TrAC.

[48] Dasenaki M.E., Bletsou A.A., Koulis G.A., Thomaidis N.S. (2015). Qualitative multiresidue screening method for 143 veterinary drugs and pharmaceuticals in milk and fish tissue using liquid chromatography quadrupole-time-of-flight mass spectrometry. J. Agric. Food Chem..

[49] Turnipseed S.B., Storey J.M., Lohne J.J., Andersen W.C., Burger R., Johnson A.S., Madson M.R. (2017). Wide-scope screening method for multiclass veterinary drug residues in fish, shrimp, and eel using liquid chromatography–quadrupole high-resolution mass spectrometry. J. Agric. Food Chem..

[50] Boix C., Ibáñez M., Sancho J.V., León N., Yusá V., Hernández F. (2014). Qualitative screening of 116 veterinary drugs in feed by liquid chromatography–high resolution mass spectrometry: Potential application to quantitative analysis. Food Chem..

[51] Stolker A.A.M., Rutgers P., Oosterink E., Lasaroms J.J.P., Peters R.J.B., van Rhijn J.A., Nielen M.W.F. (2008). Comprehensive screening and quantification of veterinary drugs in milk using UPLC-ToF-MS. Anal. Bioanal. Chem..

[52] Turnipseed S.B., Lohne J.J., Storey J.M., Andersen W.C., Young S.L., Carr J.R., Madson M.R. (2014). Challenges in implementing a screening method for veterinary drugs in milk using liquid chromatography quadrupole time-of-flight mass spectrometry. J. Agric. Food Chem..

[53] Kaklamanos G., Vincent U., Holst C. (2013). Analysis of antimicrobial agents in pig feed by liquid chromatography coupled to orbitrap mass spectrometry. J. Chromatogr. A.

[54] Kaufmann A., Butcher P., Maden K., Widmer M. (2008). Quantitative multiresidue method for about 100 veterinary drugs in different meat matrices by sub 2-μm particulate high-performance liquid chromatography coupled to time of flight mass spectrometry. J. Chromatogr A.

[55] Kang J., Fan C.-L., Cao Y.-F., Wang H.-J., Peng X., Wang Z.-B., Chang Q.-Y., Hub X.-Y., Pang G.-F. (2014). Multi-residue screening of 100 multi-class veterinary drugs in milk powder by liquid chromatography coupled to quadrupole time-of-flight mass spectrometry. Anal. Methods.

[56] Kaufmann A., Butcher P., Maden K., Walker S., Widmer M. (2011). Quantification of anthelmintic drug residues in milk and muscle tissues by liquid chromatography coupled to Orbitrap and liquid chromatography coupled to tandem mass spectrometry. Talanta.

[57] Dasenaki M.E., Thomaidis N.S. (2015). Multi-residue determination of 115 veterinary drugs and pharmaceutical residues in milk powder, butter, fish tissue and eggs using liquid chromatography–tandem mass spectrometry. Anal. Chim. Acta.

[58] Mastovska K., Lightfield A.R. (2008). Streamlining methodology for the multiresidue analysis of beta-lactam antibiotics in bovine kidney using liquid chromatography-tandem mass spectrometry. J. Chromatogr. A.

[59] Freitas A., Barbosa J., Ramos F. (2014). Multi-residue and multi-class method for the determination of antibiotics in bovine muscle by ultra-high-performance liquid chromatography tandem mass spectrometry. Meat Sci..

[60] Lopes R.P., Reyes R.C., Romero-González R., Frenich A.G., Vidal J.L.M. (2012). Development and validation of a multiclass method for the determination of veterinary drug residues in chicken by ultra high performance liquid chromatography–tandem mass spectrometry. Talanta.

[61] Gros M., Rodríguez-Mozaz S., Barceló D. (2013). Rapid analysis of multiclass antibiotic residues and some of their metabolites in hospital, urban wastewater and river water by ultra-high-performance liquid chromatography coupled to quadrupole-linear ion trap tandem mass spectrometry. J. Chromatogr. A.

[62] Xu X., Xu X., Han M., Shiting Qiu S., Hou X. (2019). Development of a modified QuEChERS method based on magnetic multiwalled carbon nanotubes for the simultaneous determination of veterinary drugs, pesticides and mycotoxins in eggs by UPLC-MS/MS. Food Chem..

[63] Mitscher L.A. (1978). The Chemistry of the Tetracycline Antibiotics.

[64] Castro M.D.L., Silva M.P. (1997). Strategies for solid sample treatment. Trends Anal. Chem..

[65] Clark S.B., Turnipseed S.B., Nandre G.J., Madson M.R., Hurlbut J.A., Sofos J.N. (2002). Confirmation of phenylbutazone residues in bovine kidney by liquid chromatography/mass spectrometry. J. AOAC Int..

[66] Martines M.A.U., Davolos M.R., Jafelicci Junior M. (2000). O efeito do ultra-som em reações químicas. Quím. Nova.

[67] Bittencourt M.S., Martins M.T., Albuquerque F.G.S., Barreto F., Hoff R. (2012). High-throughput multiclass screening method for antibiotic residue analysis in meat using liquid chromatography-tandem mass spectrometry: A novel minimum sample preparation procedure. Food Addit. Contam. Part A.

[68] Kemmerich M., Demarco M., Bernardi G., Prestes O.D., Adaime M.B., Zanella R. (2020). Balls-in-tube matrix solid phase dispersion (BiT-MSPD): An innovative and simplified technique for multiresidue determination of pesticides in fruit samples. J. Chromatogr. A.

[69] Rizetti T.M., Souza M.P., Prestes O.D., Adaime M.B., Zanella R. (2018). Optimization of sample preparation by central composite design for multi-class determination of veterinary drugs in bovine muscle, kidney and liver by ultra-high-performance liquid chromatographic-tandem mass spectrometry. Food Chem..

[70] Goulart S.M., Queiroz M.E.L.R., Neves A.A., Queiroz J.H. (2008). Low-temperature clean-up method for the determination of pyrethroids in milk using gas chromatography with electron capture detection. Talanta.

[71] Vieira H.P., Neves A.A., Queiroz M.L.R. (2007). Otimização e validação da técnica de extração líquido-líquido com partição em baixa temperatura (ELL-PBT) para piretróides em água e análise por CG. Quím. Nova.

[72] Molognoni L., Daguer H., Ploêncio L.A.S., Lindner J.D. (2018). A multi-purpose tool for food inspection: Simultaneous determination of various classes of preservatives and biogenic amines in meat and fish products by LC-MS. Talanta.

[73] Oliveira L.G., Ramkumar A., Moloney M., Kurz M.H.S., Gonçalves F.F., Prestes O.D., Danaher M. (2019). Vibrational extraction QuEChERS for analysis of antiparasitic agents in fish by liquid chromatography coupled with tandem mass spectrometry. Anal. Bioanal. Chem..

[74] Zhang Y., Li X., Liu X., Cao Y., Shi Z., Sun H. (2015). Multi-class, multi-residue analysis of trace veterinary drugs in milk by rapid screening and quantification using ultra-performance liquid chromatography–quadrupole time-of-flight mass spectrometry. J. Dairy Sci..

[75] Jia W., Chu X., Yun Ling Y., Huang J., Chang J. (2014). High-throughput screening of pesticide and veterinary drug residues in baby food by liquid chromatography coupled to quadrupole Orbitrap mass spectrometry. J. Chromatogr. A.

[76] Zhao F., Gao X., Tang Z., Luo X., Wu M., Xu J., Fu X. (2017). Development of a simple multi-residue determination method of 80 veterinary drugs in Oplegnathus punctatus by liquid chromatography coupled to quadrupole Orbitrap mass spectrometry. J. Chromatogr. B.

[77] Andersson A., Pålsheden H. (1991). Comparison of the efficiency of different GLC multi-residue methods on crops containing pesticide residues. Fresenius J. Anal. Chem..

[78] Hiemstra M., Kok A. (2007). Comprehensive multi-residue method for the target analysis of pesticides in crops using liquid chromatography–tandem mass spectrometry. J. Chromatogr. A.

[79] Rejczak T., Tuzimski T. (2015). A review of recent developments and trends in the QuEChERS sample preparation approach. Open Chem..

[80] González-Curbelo M.A., Socas-Rodríguez B., Herrera-Herrera A.V., González-Sálamo J., Hernández-Borges J., Rodríguez-Delgado M.A. (2015). Evolution and applications of the QuEChERS method. TrAC.

[81] Desmarchelier A., Fan K., Tien M.M., Savoy M.-C., Tarres A., Fuger D., Goyon A., Bessaire T., Mottier P. (2018). Determination of 105 antibiotic, anti-inflammatory, antiparasitic agents and tranquilizers by LC-MS/MS based on an acidic QuEChERS-like extraction. Food Addit. Contam. Part A.

[82] Perestrelo R., Silva P., Porto-Figueira P., Pereira J.A.M., Silva C., Medina S., Câmara J.S. (2019). QuEChERS: Fundamentals, relevant improvements, applications and future trends. Anal. Chim. Acta.

[83] Lehotay S.J., Matovská K., Yun S.J. (2005). Evaluation of two fast and easy methods for pesticide residue analysis in fatty food matrixes. J. AOAC Int..

[84] Lehotay S., Son K.A., Kwon H., Koesukwiwat U., Fu W., Mastovska K., Hoh E., Leepipatpiboon N. (2010). Comparison of QuEChERS sample preparation methods for the analysis of pesticide residues in fruits and vegetables. J. Chromatogr. A.

[85] Moretti S., Dusi G., Giusepponi D., Pellicciotti S., Rossi R., Saluti G., Cruciani G., Galarini R. (2016). Screening and confirmatory method for multiclass determination of 62 antibiotics in meat. J. Chromatograp. A.

[86] Borràs S., Kaufmann A., Companyó R. (2013). Correlation of precursor and product ions in single-stage high resolution mass spectrometry. A tool for detecting diagnostic ions and improving the precursor elemental composition elucidation. Anal. Chim. Acta.

[87] Kaufmann A. (2020). High-resolution mass spectrometry for bioanalytical applications: Is this the new gold standard?. J. Mass Spectrom..

[88] Vanini G., Barra T.A., Souza L.M., Madeira N.C.L., Gomes A.O., Romão W., Azevedo D.A. (2020). Characterization of nonvolatile polar compounds from Brazilian oils by electrospray ionization with FT-ICR MS and Orbitrap-MS. Fuel.

[89] Kaufmann A., Butcher P., Maden K., Walker S., Widmer M. (2015). Reliability of veterinary drug residue confirmation: High resolution mass spectrometry versus tandem mass spectrometry. Anal. Chim. Acta.

[90] Kaufmann A., Mol W.G. (2016). Product ion isotopologue pattern: A tool to improve the reliability of elemental composition elucidations of unknown compounds in complex matrices. Rapid Commun. Mass Spectrom..

[91] Lehotay S.J., Sapozhnikova Y., Mol H.G. (2015). Current issues involving screening and identification of chemical contaminants in foods by mass spectrometry. TrAC.

[92] Moretti S., Lega F., Rigoni L., Saluti G., Giusepponi D., Gioiello A., Manuali E., Rossi R., Galarini R. (2018). Multiclass screening method to detect more than fifty banned substances in bovine bile and urine. Anal. Chim. Acta.

[93] Varenina I., Bilandžić N., Luburić Đ.B., Kolanović B.S., Varga I. (2022). High resolution mass spectrometry method for the determination of 13 antibiotic groups in bovine, swine, poultry and fish meat: An effective screening and confirmation analysis approach for routine laboratories. Food Control.

[94] Brasil. Ministério da Agricultura, Pecuária e Abastecimento (2019). Instrução Normativa nº 5, de 23 de Abril de 2019. Aprova o Plano de Amostragem e os Limites de Referência para o Plano Nacional de Controle de Resíduos e Contaminantes em Produtos de Origem Animal—PNCRC de 2019 para as Cadeias de Carnes Bovina, Suína, Caprina, Ovina, Equina, de Coelho, de aves e de Avestruz, de Leite, Pescado, mel e Ovos. Diário Oficial da União.

[95] Food and Agriculture Organization (2021). Maximum Residue Limits (MRLs) and Risk Management Recommendations (RMRs) for Residues of Veterinary Drugs in Foods.

[96] European Union (2010). Commission Regulation (EU) No 37/2010 of 22 December 2009 on Pharmacologically Active Substances and Their Classification Regarding Maximum Residue Limits in Foodstuffs of Animal Origin (Text with EEA Relevance). Off. J. Eur. Union.

[97] Paula R.A.O. (2017). Resíduos de Antimicrobianos em Produtos de Origem Animal: Análise Crítica das Bulas e Avaliação Histórica dos Programas de Monitoramento no Brasil. Master’s Thesis.

[98] European Commission (2021). Commission Implementing Regulation (EU) 2021/808 of 22 March 2021 on the performance of analytical methods for residues of pharmacologically active substances used in food-producing animals and on the interpretation of results as well as on the methods to be used for sampling and repealing Decisions 2002/657/EC and 98/179/EC. Off. J. Eur. Union.

[99] European Comission (2010). Community Reference Laboratories Residues. Guidelines for the Validation of Screening Methods for Residues of Veterinary Medicines: Initial Validation and Transfer.

[100] Gondim C.S., Coelho O.A.M., Alvarenga R.L., Junqueira R.G., Souza S.V.C. (2014). An appropriate and systematized procedure for validating qualitative methods: Its application in the detection of sulfonamide residues in raw milk. Anal. Chim. Acta..

[101] Agência Nacional de Vigilância Sanitária (2022). Instrução Normativa nº 162, de 1 de julho de 2022. Estabelece a Ingestão Diária Aceitável (IDA), a Dose de Referência Aguda (DRfA) e os Limites Máximos de Resíduos (LMR) para Insumos Farmacêuticos Ativos (IFA) de Medicamentos Veterinários em Alimentos de Origem Animal. Diário Oficial da União.

[102] US Food & Drug Administration (2022). CFR—Code of Federal Regulations Title 21: Food and Drug.

[103] The Japan Food Chemical Research Foundation (2022). Maximum Residue Limits (MRLs) List of Agricultural Chemicals in Foods.

[104] Parker C. (2019). Agricultural and Veterinary Chemicals Code (MRL Standard) Instrument 2019.

[105] Huet A.-C., Bienenmann-Ploum M., Vincent U., Delahaut P. (2013). Screening methods and recent developments in the detection of anticoccidials. Anal. Bioanal. Chem..

[106] Wode F., van Baar P., Dünnbier U., Hecht F., Taute T., Jekel M., Reemtsma T. (2015). Search for over 2000 current and legacy micropollutants on a wastewater infiltration site with a UPLC-high resolution MS target screening method. Water Res..

[107] Chemaxon (2022). logP Plugin.

[108] Chemaxon (2022). Pka Plugin.

[109] Chemaxon (2022). Solubility prediction.

[110] Perkin Elmer Informatics (2019). Chemdraw Vesion 18.1: User Guide.

[111] Chemspider (2022). Search ChemSpider.

[112] National Library of Medicine (2022). PubChem: Explore Chemistry.

