# Supplementary material for: Critical Evaluation of Two Qualitative Analytical Approaches for Multiclass Determination of Veterinary Drugs in Bovine Muscle Using UHPLC-Q-Orbitrap: The Wind of Change in Brazilian Monitoring
Source: Molecules. 2023 May 17;28(10):4150. doi: 10.3390/molecules28104150 (PMC10222520; doi:10.3390/molecules28104150)

Supplementary Materials

Critical Evaluation of Two Qualitative Analytical Approaches for Multiclass Determination of Veterinary Drugs in Bovine Muscle Using UHPLC-Q-Orbitrap: The Wind of Change in Brazilian Monitoring

Ramon Alves de Oliveira Paula [1,*], Carina de Souza Gondim [1], Eduardo Morgado Schmidt [2], Maria Helena Glicério Marcelina Diniz [3], Mary Ane Gonçalves Lana [3] and Leandro Soares de Oliveira [1,4,*]

[1] Postgraduate Program in Food Science, Department of Food Science (ALM), Faculty of Pharmacy (FAFAR), Federal University of Minas Gerais (UFMG), Campus da UFMG, Antônio Carlos Avenue 6627, Belo Horizonte 31270-010, Brazil; carinasgondim@gmail.com

[2] Nova Analítica Importações e Exportações LTDA, Assungui Street, 432, Vila Gumercindo, São Paulo 04131-000, Brazil; eduardomschmidt@yahoo.com

[3] Food of the Agricultural Defense Federal Laboratory of Minas Gerais, Ministry of Agriculture and Livestock, Rômulo Joviano Avenue, s/nº, Centro, Pedro Leopoldo 33600-000, Brazil; maria.diniz@agricultura.gov.br (M.H.G.M.D.); mary.lana@agricultura.gov.br (M.A.G.L.)

[4] Department of Mechanical Engineering, Engineering School, Federal University of Minas Gerais (UFMG), Campus da UFMG, Antônio Carlos Avenue 6627, Belo Horizonte 31270-010, Brazil

* Correspondence: alvesfarmacia@yahoo.com.br (R.A.d.O.P.); leandro@demec.ufmg.br (L.S.d.O.); Tel.: +55-31-3409-3517 (L.S.d.O.)

Table of contents

Table S1. Concentration of the standard solution, maximum residue limit (MRL), minimum required performance level (MRPL) in bovine muscle……………..…………………….................................3

Table S2. Chromatographic conditions of the different methods evaluated for the Accela 1,250 Pump UHPLC system…………………………………………………………………………...................8

Table S3. Mass spectrometer conditions of different methods evaluated for the Q-Exactive Orbitrap HRMS system…………………………………………………………………………........9

Table S4. Physicochemical properties of veterinary drugs and the main conditions determined by Q-Exactive Orbitrap HRMS…………………………………..…………………………………..…10

Table S5. Retention time of veterinary drugs and their metabolites evaluated by Q-Exactive Orbitrap HRMS…………………………………………………………………………..…..…………... 168

Figure S1. General flowchart of the sample preparation procedure according to method A……….174

Figure S2. General flowchart of the sample preparation procedure according to method B……….175

Figure S3. Analyte identification criteria...176

Figure S4. Examples extracted mass traces of a spiked bovine muscle tissue at 2 MRL according to the established criteria for analyte identification…..177

Table S1. Concentration of the standard solution, maximum residue limit (MRL), minimum required performance level (MRPL) in bovine muscle.

Group/Class - Pharmacologically active Substance	Solvent used	Stock standard concentration (µg mL^{-1})	MRL in bovine muscle (µg Kg^{-1})	Reference
**Antimicrobials/Aminoglycosides**				
Amikacin	Water	512.6	500[a]	
Apramycin	Water	486.8	1000	[101]
Dihydrostreptomycin	Water	499.2	600	[101]
Spectinomycin	Water	511.3	500	[101]
Streptomycin	Water	504.1	500	[101]
Gentamicin	Water	510.8	100	[101]
Hygromycin B	Water	489.8	500[a]	
Kanamycin A	Water	505.4	100	[101]
Neomycin B	Water	494.2	500	[101]
Tobramycin	Water	200.0	500[a]	
**Antimicrobials/Amphenicols**				
Chloramphenicol	Methanol	100.2	0.30	[94]
Florfenicol	Methanol	100.0	200	[101]
Thiamphenicol	Methanol	100.3	50	[96]
**Antimicrobials/Betalactams**				
Amoxicillin	Water	195.3	50	[101]
Ampicillin	Water	194.7	50	[101]
Cephalexin	Water	297.8	200	[101]
Cefalonium	DMSO (sonicated)	194.8	10	[103]
Cephapirin	Acetonitrile/Water 75:25 (v/v)	199.8	50	[101]
Cefazolin	Water	200.7	50	[103]
Cefoperazone	Acetonitrile/Water 75:25 (v/v)	79.7	50[a]	
Cefquinome	12.5% DMSO/ methanol	167.1	50	[101]
Cloxacillin	Water	320.9	300	[101]
Dicloxacillin	Water	338.3	300	[101]
Nafcillin	Water	347.9	300	[101]
Oxacillin	Water	516.4	300	[96]
Benzylpenicillin	Water	196.9	50	[101]
Phenoxymethyl penicillin	Water	203.5	25[b,c]	[101]
**Antimicrobials/Lincosamides**				
Clindamycin	Methanol	474.2	50[a]	
Lincomycin	Methanol	491.0	100	[101]
**Antimicrobials/Macrolides**				
Azithromycin	Water	101.2	50	[94]

Group/Class - Pharmacologically active Substance	Solvent used	Stock standard concentration (μg mL^{-1})	MRL in bovine muscle (μg Kg^{-1})	Reference
Erythromycin A	Methanol	472.8	200	[101]
Spiramycin I	Methanol	497.7	200	[101]
Tilmicosin	Methanol	500.4	100	[101]
Tylosin A	Methanol	496.8	100	[101]
**Antimicrobials/Quinolones-Fluoroquinolones**				
Nalidixic acid	NaOH 1 mol L^{-1}/Methanol	1001.8	20	[94]
Oxolinic acid	NaOH 1 mol L^{-1}/Methanol	125.0	100	[101]
Ciprofloxacin	NaOH 1 mol L^{-1}/Methanol	300.9	100	[101]
Danofloxacin	Acetone/Methanol	995.0	200	[101]
Difloxacin	Acetone/Methanol	999.0	400	[101]
Enrofloxacin	Acetone/Methanol	1004.9	100	[101]
Flumequine	NaOH 1 mol L^{-1}/Methanol	204.0	500	[101]
Norfloxacin	Acetone/Methanol	1005.0	20	[94]
Sarafloxacin	Acetone/Methanol	220.1	10[d]	[101]
**Antimicrobials/Sulphonamides**				
Sulfachlorpyridazine	Methanol	1000.0	100	[101]
Sulfadiazine	Methanol	500.0	100	[101]
Sulfadimethoxine	Methanol	250.0	100	[101]
Sulfadoxine	Methanol	250.0	100	[101]
Sulfisoxazole/Sulfafurazole	Methanol	1000.0	100	[101]
Sulfamerazine	Methanol	1000.0	100	[101]
Sulfamethazine/Sulfadimidine	Methanol	250.0	100	[101]
Sulfathiazole	Methanol	1000.0	100	[101]
Sulfamethoxazole	Methanol	1000.0	100	[101]
Sulfaquinoxaline	Acetone/Methanol	1000.0	100	[101]
**Antimicrobials/Tetracyclines**				
Chlortetracycline	Methanol	511.7	200	[101]
Doxycycline	Methanol	463.0	100	[101]
Oxytetracycline	Methanol	498.7	200	[101]
Tetracycline	Methanol	515.8	200	[101]
**Antimicrobials/Others**				
Bromhexine	Methanol	490.3	10	
Dapsone	Methanol	393.6	10	
Rifampicin	Methanol	367.7	10	
Tiamulin	Methanol	171.3	100[c,d]	[101]
**Antimicrobials Prospect/Quinolones-Fluoroquinolones**				
Marbofloxacin	NaOH 1 mol L^{-1}/Methanol	100.0	150	[101]
**Antimicrobials Prospect/Macrolides**				

Group/Class - Pharmacologically active Substance	Solvent used	Stock standard concentration (μg mL^{-1})	MRL in bovine muscle (μg Kg^{-1})	Reference
Josamycin/Leucomycin A_3	Methanol	202.3	100[a]	
Leucomycin/Leucomycin A_5	Methanol	100.3	100[a]	
Tildipirosin	Methanol	446.3	400	[101]
Tulathromycin A	Methanol	270.6	300	[101]
**Antimicrobials Prospect/ Sulfonamides**				
Phthalylsulfathiazole	Methanol	251.0	100	[101]
Sulfacetamide/*N*-Sulfanilylacetamide	Methanol	250.2	100	[101]
Sulfamethoxypyridazine	Methanol	250.0	100	[101]
**Antimicrobials Prospect/Others**				
Diminazene	Methanol	218.5	500	[101]
Fosfomycin	Water	184.3	500	[103]
Isoniazid	6% DMSO in methanol	640.6	10	
Novobiocin	Methanol	287.1	1000	[102]
Rifaximin	Methanol/Water 50:50 (v/v)	282.9	10	
**Antibiotic Growth Promoters**				
Dichloroisoeverninic acid	Methanol	1000.0	800	[103]
Halquinol/Chlorhydroxyquinoline	12.5% DMSO in methanol	102.6	40[c]	[101]
Virginiamycin M_1	Methanol	100.9	100	[103]
**Anticoccidials**				
Amprolium	Methanol	1000.0	500	[102]
Clopidol	Methanol	1000.0	200	[103]
Diaveridine	Methanol	1000.0	50[b]	[103]
Decoquinate	Methanol	1000.0	1000	[103]
Diclazuril	DMSO	1000.0	50	[103]
Ethopabate	Methanol	1000.0	500[b]	[102]
Lasalocid A	Acetonitrile	1000.0	10	[101]
Maduramicin	Methanol	100.6	30[b]	[101]
Monensin A	Methanol	101.9	10	[101]
Narasin A	Methanol	1000.0	15	[101]
4,4′-Dinitrocarbanilide – DNC	DMSO	1000.0	4000[d]	[101]
Robenidine	Methanol	1000.0	200[d]	[101]
Salinomycin	DMSO	1000.0	20	[103]
Toltrazuril	DMSO	1000.0	100	[101]
Trimethoprim	Methanol	1000.0	50	[101]
**Anti-inflammatories/Steroidal**				
Prednisolone	Methanol	270.2	4	[101]
Prednisone	Methanol	276.4	4	
**Anti-inflammatories Prospect/Steroidal**				

Group/Class - Pharmacologically active Substance	Solvent used	Stock standard concentration (μg mL^{-1})	MRL in bovine muscle (μg Kg^{-1})	Reference
Isoflupredone acetate/9-Fluoroprednisolone acetate	Methanol	169.3	10	
Flumetasone	Methanol	352.8	10	
**Anti-Inflammatory/Non-Steroidal**				
Mefenamic acid	Methanol	203.3	20	[94]
Tolfenamic acid	Acetonitrile	184.5	50	[101]
Carprofen	6% DMSO in methanol	201.6	500	[101]
Ketoprofen	Acetonitrile	180.5	50	[103]
Diclofenac	6% DMSO in methanol	204.0	5	[101]
Flunixin	Methanol	99.2	20	[101]
Indomethacin	Methanol	177.5	20	[94]
Meloxicam	12.5% DMSO in methanol	192.0	20	[101]
Naproxen	6% DMSO in methanol	162.2	20	[94]
Nimesulide	Methanol	205.0	20	[94]
Piroxycam	DMSO	100.8	20	[94]
Propyphenazone	Methanol	193.4	20	[94]
**Antiparasitic/Avermectins**				
Avermectin B_{1a}/Abamectin B_{1a}	Methanol	113.1	20	[103]
Doramectin	Methanol	109.4	10	[101]
Emamectin B_{1a}	Methanol	151.1	2	[103]
Eprinomectin B_{1a}	Methanol	89.8	100	[101]
Ivermectin B_{1a}/22,23-Dihydroavermectin B_1	Methanol	93.2	30	[101]
Moxidectin	Methanol	88.6	20	[101]
**Antiparasitic/Benzimidazoles**				
Albendazole	12.5% DMSO in acetonitrile	205.5	100	[101]
Albendazole sulfone	12.5% DMSO in methanol	216.4	100	[101]
Albendazole sulfoxide	12.5% DMSO in methanol	147.6	100	[101]
Closantel	Acetonitrile	210.1	1000	[101]
Febantel	Acetonitrile	254.4	100	[101]
Fenbendazole	12.5% DMSO in acetonitrile	221.0	100	[101]
Fenbendazole sulfone	12.5% DMSO in acetonitrile	181.4	100	[101]
Flubendazole	12.5% DMSO in acetonitrile	213.7	20	[103]
2-Aminoflubendazole	12.5% DMSO in acetonitrile	134.7	20	[103]
Levamisole	Acetonitrile/ DMSO/Methanol	217.0	10	[101]
Mebendazole	Acetonitrile	204.3	20	[104]

Group/Class - Pharmacologically active Substance	Solvent used	Stock standard concentration (µg mL^{-1})	MRL in bovine muscle (µg Kg^{-1})	Reference
Oxibendazole	Methanol	103.2	100	[101]
Oxfendazole	Acetonitrile/ DMSO	211.1	100	[101]
Thiabendazole	Acetonitrile	164.8	100	[101]
Triclabendazole	Acetonitrile	404.5	250	[101]
**Antiparasitic/Phenylpyrazoles**				
Fipronil	Methanol	134.9	500	[103]
Fipronil sulfone	Methanol	87.2	500	[103]
**Antiparasitic/Nitroimidazoles**				
Dimetridazole	Acetonitrile/ DMSO	225.6	10	
Hydroxydimetridazole/HMMNI	Acetonitrile	203.3	10	
Ipronidazole	Acetonitrile	203.3	3	[94]
Hydroxy Ipronidazole	Acetonitrile	94.1	3	[94]
Metronidazole	Acetonitrile/ DMSO	246.5	10	
Hydroxymetronidazole	Acetonitrile/ DMSO	220.7	10	
Ronidazole/1-Methyl-2-carbamoyloxymethyl-5-nitroimidazole	Acetonitrile/ DMSO	203.8	10	
**Antiparasitic/Isoquinoline-pyrazines**				
Praziquantel	Methanol	240.5	300	[103]
**Beta-Agonists**				
Cimaterol	Methanol	100.2	10	
Clenbuterol	Methanol	1000.0	0.2	[101]
Ractopamine	Methanol	100.1	10	[101]
Salbutamol	Methanol	1000.0	10	
Zilpaterol	Methanol	100.8	0.5	[101]
**Sedatives**				
Acepromazine	Methanol	101.1	10	
Azaperol	Methanol	101.4	60[c]	[101]
Azaperone	Methanol	102.7	60[c]	[101]
Carazolol	Methanol	101.2	5	[101]
Chlorpromazine	Methanol	211.5	10	
**Prospecting sedatives**				
Xylazine	Methanol	100.2	20	[103]
**Corants**				
Gentian violet/Crystal violet	Acetonitrile	98.9	10	
Leucocrystal Violet	Acetonitrile	290.8	10	
Malachite green	Acetonitrile	140.7	10	
Leucomalachite green	Acetonitrile	113.9	10	

[a]Class - Pharmacologically active substance; [b]Chicken, muscle; [c]Pig, muscle; [d]Broiler chicken, muscle. Abbreviations: µg/mL: microgram per milliliter; µg Kg^{-1}: micrograms per kilogram; DMSO: dimethyl sulfoxide.

Table S2. Chromatographic conditions of the different methods evaluated for the Accela 1,250 Pump UHPLC system.

Parameter	Condition		
	Method A	Method B	
		Aqueous extract	Organic extract (ACN)
Column and Pre-column	Acquity UHPLC® BEH C18 (50 mm x 2.1 mm d.i x 1.7 µm). Waters<br>VanGuardAcquity UPLC® BEH C18 (5 mm x 2.1 mm d.i x 1.7 µm). Waters		
Column and inlet temperature	30 ± 5°C and 15 °C	35 ± 5°C and 15°C	30 ± 5°C and 15°C
Injection volume	5 µL	10 µL	
Mobile phase	MPA: 0.01% formic acid in water<br>MPB: MeOH	MPA: Amonium formate 5 mmol L^{-1} + 0.1% formic acid in water<br>MPB: H_2O/ACN 5:95 + 0.1% formic acid	MPA: Amonium formate 0.2 mol L^{-1}+ 0.1% formic acid<br>MPB: H_2O/ACN 5:95 + 0.1% formic acid
Gradient	0.0 min: 95% MPA. 5% MPB → 7.0 min: 0% MPA. 100% MPB → 10.0 min: 0% MPA. 100% MPB → 10.1 min: 95% MPA. 5% MPB → 17 min: 95% MPA. 5% MPB	0.0 min: 100% MPA. 0% MPB →2.0 min: 80% MPA. 20% MPB → 3.5 min: 5% MPA. 95% MPB → 4.5 min: 5% MPA. 95% MPB → 5.0 min: 100% MPA. 0% MPB	0.0 min: 100% MPA. 0% MPB → 1.5 min: 100% MPA. 0% MPB → 2.5 min: 2% MPA. 98% MPB → 3.5 min: 2% MPA. 98% MPB → 4.0 min: 100% MPA. 0% MPB
Flow	0.1 mL min^{-1}	0.4 mL min^{-1}	
Run time	17.0 min	20.0 min	

Abbreviations: µL: microliter; min: minute; mL min^{-1}: milliliter per minute; ACN: acetonitrile; H_2O: water; MeOH: methanol; MPA: mobile phase A; MPB: mobile phase B.

Table S3. Mass spectrometer conditions of different methods evaluated for the Q-Exactive Orbitrap HRMS system.

Parameter ($HESI^+$), ($HESI^-$)	
Ion source	
Spray voltage	3.9 kV ($HESI^+$); 2.9 kV ($HESI^-$)
Capillary temperature	350°C
Sheath gas	40 arb
Auxiliary gas	15 arb
S-Lens RF	50 arb
Full-MS	
Microscans	1
Resolution	35,000 FWHM
Target automatic gain control (AGC targed)	3.0×10^6
Maximum injection time (IT)	100 ms
Scan range	65-975 *m/z*
AIF	
Microscans	1
Resolution	17,500 FWHM
AGC targed	3.0×10^6
Maximum injection time	100 ms
Normalized collision energy (NCE)	10, 35 and 80 eV
Scan range	65-975 *m/z*

Abbreviations: Arb: arbitrary units; FWHM: full width at half maximum; ms: milli seconds; *m/z*: mass to charge.

Table S4. Physicochemical properties of veterinary drugs and the main conditions determined by Q-Exactive Orbitrap HRMS.

**Group/Class - Pharmacologically active Substance**	**Physicochemical properties**	**Monitored ion**	**Theoretical exact mass MS (*m/z*)**	**Fragment ions (*m/z*) MS²**		
				[M+H]⁺	[M-H]⁻	Others
**Antimicrobials/Aminoglycosides**						
Amikacin<br><br>CAS registration number:<br>37517-28-5<br><br>ChemSpider ID:<br>34635<br><br>PubChem CID:<br>37768<br><br>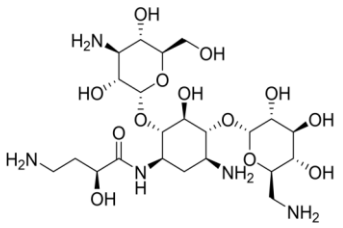	Molecular formula:<br>$C_{22}H_{43}N_5O_{13}$<br><br>Monoisotopic mass (Da): 585.2852<br><br>pKa (strongest acidic): 12.16<br><br>pKa (strongest basic): 9.61<br><br>Partition coefficient (Log P): -8.584<br><br>Intrinsic solubility (mg mL^{-1}): 0.406 (High)	$[M+H]^+$;<br>$[M-H]^-$	586.2930;<br>584.2785	425.2242;<br>264.1554;<br>163.1077;<br>324.1765;<br>467.2348	405.1993;<br>423.2099;<br>287.1238;<br>270.0986;<br>244.1305	

Group/Class - Pharmacologically active Substance	Physicochemical properties	Monitored ion	Theoretical exact mass MS (*m/z*)	Fragment ions (*m/z*) MS^2		
				$[M+H]^+$	$[M-H]^-$	Others
Apramycin<br><br>CAS registration number:<br>37321-09-8<br><br>ChemSpider ID:<br>2339128<br><br>PubChem CID:<br>3081545<br><br>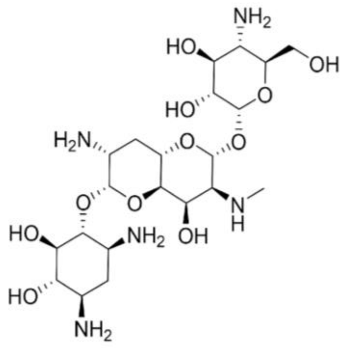	Molecular formula:<br>$C_{21}H_{41}N_5O_{11}$<br><br>Monoisotopic mass (Da): 539.2797<br><br>pKa (strongest acidic):<br>12.23<br><br>pKa (strongest basic):<br>9.05<br><br>Partition coefficient (Log P): -6.508<br><br>Intrinsic solubility (mg mL^{-1}): 1.526 (High)	$[M+H]^+$	540.2875;<br>270.6474	378.1841;<br>344.1816;<br>217.1183;<br>523.2610;<br>163.1077		$[M+2H]^{2+}$<br>217.1183;<br>163.1077

Group/Class - Pharmacologically active Substance	Physicochemical properties	Monitored ion	Theoretical exact mass MS (*m/z*)	Fragment ions (*m/z*) MS^2		
				[M+H]$^+$	[M-H]$^-$	Others
Dihydrostreptomycin<br><br>CAS registration number:<br>128-46-1<br><br>ChemSpider ID:<br>388489<br><br>PubChem CID:<br>439369<br><br>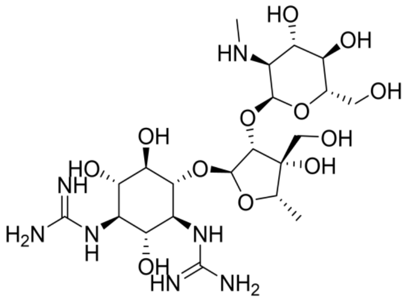	Molecular formula:<br>$C_{21}H_{41}N_7O_{12}$<br><br>Monoisotopic mass (Da): 583.2808<br><br>pKa (strongest acidic):<br>11.91<br><br>pKa (strongest basic):<br>10.91<br><br>Partition coefficient (Log P): -7.35<br><br>Intrinsic solubility (mg mL^{-1}):<br>-0.185(High)	[M+H]$^+$;<br>[M-H]$^-$;<br>[M+2H]$^{2+}$	584.2886;<br>582.2740;<br>292.6479	263.1462;<br>221.1244;<br>176.0917;<br>186.0873;<br>409.2041	407.1898;<br>261.1319;<br>234.0986;<br>243.1214;<br>219.1101	[M+2H]$^{2+}$<br>263.1462;<br>176.0917;<br>158.0812;<br>110.0600;<br>409.2041

Group/Class - Pharmacologically active Substance	Physicochemical properties	Monitored ion	Theoretical exact mass MS (*m/z*)	Fragment ions (*m/z*) MS^2		
				[M+H]$^+$	[M-H]$^-$	Others
Spectinomycin<br><br>CAS registration number: 1695-77-8<br><br>ChemSpider ID: -<br><br>PubChem CID: 15541<br><br>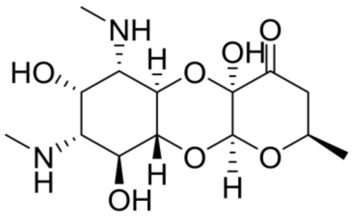	Molecular formula: $C_{14}H_{24}N_2O_7$<br><br>Monoisotopic mass (Da): 332.1578<br><br>pKa (strongest acidic): 8.98<br><br>pKa (strongest basic): 8.38<br><br>Partition coefficient (Log P): -2.076<br><br>Intrinsic solubility (mg mL^{-1}): -0.846 (High)	[M+H]$^+$;<br>[M+H$_3$O]$^+$	333.1656;<br>352.1840	98.0600;<br>94.0651;<br>207.1339<br>140.0706;<br>110.0600		[M+H$_3$O]$^+$<br>351.1762;<br>207.1339;<br>140.0706;<br>189.1234;<br>116.0706

**Group/Class - Pharmacologically active Substance**	**Physicochemical properties**	**Monitored ion**	**Theoretical exact mass MS (*m/z*)**	**Fragment ions (*m/z*) MS^2**		
				$[M+H]^+$	$[M-H]^-$	Others
Streptomycin<br><br>CAS registration number:<br>57-92-1<br><br>ChemSpider ID:<br>18508<br><br>PubChem CID:<br>19649<br><br>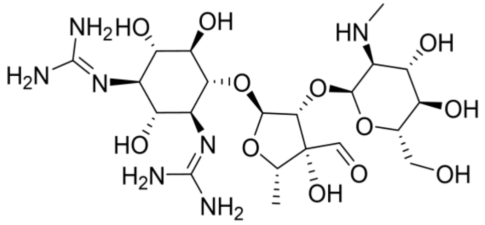	Molecular formula:<br>$C_{21}H_{39}N_7O_{12}$<br><br>Monoisotopic mass (Da): 581.2651<br><br>pKa (strongest acidic):<br>11.16<br><br>pKa (strongest basic):<br>10.73<br><br>Partition coefficient (Log P): -7.192<br><br>Intrinsic solubility (mg mL^{-1}): -0.378 (High)	$[M+H]^+$;<br>$[M-H]^-$;<br>$[M+2H]^{2+}$;<br>$[M+H_2O+H]^+$;<br>$[M+H_2O+2H]^{2+}$	582.2730;<br>580.2584;<br>291.6401;<br>600.2835;<br>300.6454	263.1462;<br>407.1885;<br>540.2511;<br>176.0917;<br>246.1197	261.1319;<br>381.1742;<br>318.1197;<br>219.1101;<br>119.0352	$[M+2H]^{2+}$<br>263.1462;<br>176.0917;<br>158.0812;<br>110.0600<br><br>$[M+H_2O+H]^+$<br>101.0597;<br>263.1462;<br>176.0917<br><br>$[M+H_2O+2H]^{2+}$<br>101.0597;<br>263.1462;<br>176.0917

Group/Class - Pharmacologically active Substance	Physicochemical properties	Monitored ion	Theoretical exact mass MS (*m/z*)	Fragment ions (*m/z*) MS^2		
				$[M+H]^+$	$[M-H]^-$	Others
Gentamicin (Gentamicin C_1)<br><br>CAS registration number: 25876-10-2<br><br>ChemSpider ID: 65328<br><br>PubChem CID: 72395<br><br>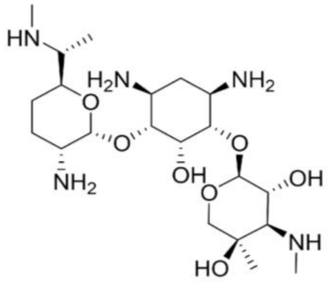 	Molecular formula: $C_{21}H_{43}N_5O_7$<br><br>Monoisotopic mass (Da): 477.3157<br><br>pKa (strongest acidic): 12.55<br><br>pKa (strongest basic): 10.03<br><br>Partition coefficient (Log P): -3.137<br><br>Intrinsic solubility (mg mL^{-1}): -0.419 (High)	$[M+H]^+$; $[M+2H]^{2+}$	478.3235; 239.6654	160.0968; 322.1973; 157.1335		$[M+2H]^{2+}$ 160.0968; 322.1973

Group/Class - Pharmacologically active Substance	Physicochemical properties	Monitored ion	Theoretical exact mass MS (*m/z*)	Fragment ions (*m/z*) MS^2		
				$[M+H]^+$	$[M-H]^-$	Others
Gentamicin (Gentamicin C_{1a})<br><br>CAS registration number: 26098-04-4<br><br>ChemSpider ID: 65329<br><br>PubChem CID: 72396<br><br>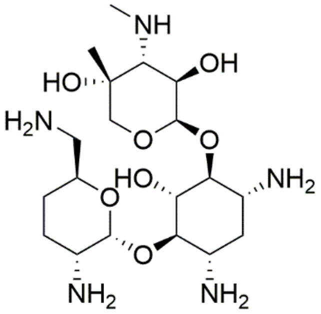	Molecular formula: $C_{19}H_{39}N_5O_7$<br><br>Monoisotopic mass (Da): 449.2844<br><br>pKa (strongest acidic): 12.55<br><br>pKa (strongest basic): 9.66<br><br>Partition coefficient (Log P): -3.986<br><br>Intrinsic solubility (mg mL^{-1}): -0.335 (High)	$[M+2H]^{2+}$	225.6498			$[M+2H]^{2+}$ 129.1022; 322.1973

Group/Class - Pharmacologically active Substance	Physicochemical properties	Monitored ion	Theoretical exact mass MS (*m/z*)	Fragment ions (*m/z*) MS^2		
				$[M+H]^+$	$[M-H]^-$	Others
Gentamicin (Gentamicin C_2-C_{2a})<br><br>CAS registration number:<br>25876-11-3<br><br>ChemSpider ID:<br>65330<br><br>PubChem CID:<br>72397<br><br>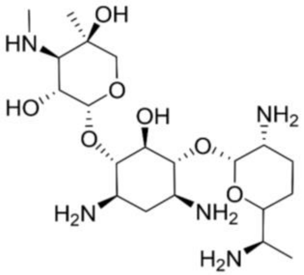	Molecular formula:<br>$C_{20}H_{41}N_5O_7$<br><br>Monoisotopic mass (Da): 463.3001<br><br>pKa (strongest acidic): 12.55<br><br>pKa (strongest basic): 9.87<br><br>Partition coefficient (Log P): -3.57<br><br>Intrinsic solubility (mg mL^{-1}): -0.638 (High)	$[M+H]^+$;<br>$[M+2H]^{2+}$	464.3079;<br>232.6576	160.0968;<br>163.1077;<br>322.1973		$[M+2H]^{2+}$<br>160.0968;<br>163.1077

Group/Class - Pharmacologically active Substance	Physicochemical properties	Monitored ion	Theoretical exact mass MS (*m/z*)	Fragment ions (*m/z*) MS^2		
				$[M+H]^+$	$[M-H]^-$	Others
Hygromycin (Hygromycin B)<br><br>CAS registration number:<br>31282-04-9<br><br>ChemSpider ID:<br>28634700<br><br>PubChem CID:<br>56928061<br><br>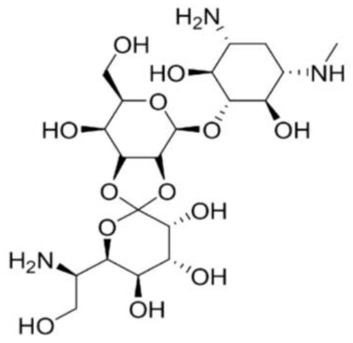	Molecular formula:<br>$C_{20}H_{37}N_3O_{13}$<br><br>Monoisotopic mass (Da): 527.2321<br><br>pKa (strongest acidic): 11.4<br><br>pKa (strongest basic): 9.4<br><br>Partition coefficient (Log P): -6.372<br><br>Intrinsic solubility (mg mL^{-1}): -0.323 (High)	$[M+H]^+$;<br>$[M-H]^-$;<br>$[M+2H]^{2+}$	528.2399;<br>526.2254;<br>264.6236	352.1238;<br>177.1234;<br>257.0656;<br>150.0761;<br>303.1551	206.0659;<br>319.1500;<br>188.0553;<br>368.1187	$[M+2H]^{2+}$<br>177.1234;<br>159.1128;<br>132.0655;<br>190.0710

Group/Class - Pharmacologically active Substance	Physicochemical properties	Monitored ion	Theoretical exact mass MS (*m/z*)	Fragment ions (*m/z*) MS^2		
				$[M+H]^+$	$[M-H]^-$	Others
Kanamycin (Kanamycin A)<br><br>CAS registration number:<br>59-01-8<br><br>ChemSpider ID:<br>388665<br><br>PubChem CID:<br>6032<br><br>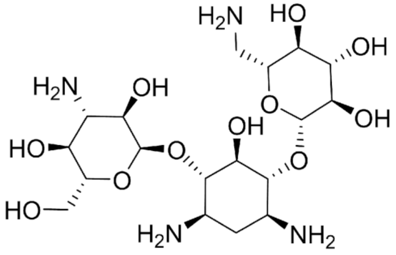	Molecular formula:<br>$C_{18}H_{36}N_4O_{11}$<br><br>Monoisotopic mass (Da): 484.2375<br><br>pKa (strongest acidic): 12.05<br><br>pKa (strongest basic): 9.34<br><br>Partition coefficient (Log P): -7.061<br><br>Intrinsic solubility (mg mL^{-1}): 0.24 (High)	$[M+H]^+$;<br>$[M-H]^-$;<br>$[M+Na]^+$	485.2453;<br>483.2308;<br>507.2273	163.1077;<br>84.0444;<br>68.0495;<br>102.0550;<br>324.1756	322.1622;<br>354.0694	$[M+Na]^+$<br>346.1582;<br>163.1077;<br>249.0921;<br>263.2345

Group/Class - Pharmacologically active Substance	Physicochemical properties	Monitored ion	Theoretical exact mass MS (*m/z*)	Fragment ions (*m/z*) MS^2		
				$[M+H]^+$	$[M-H]^-$	Others
Neomycin (Neomycin B)<br><br>CAS registration number:<br>119-04-0<br><br>ChemSpider ID:<br>8075<br><br>PubChem CID:<br>8378<br><br>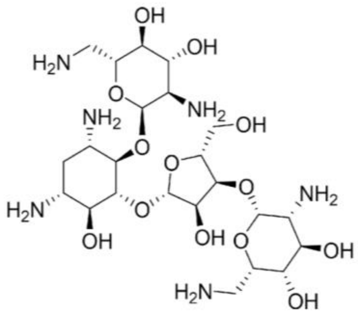	Molecular formula:<br>$C_{23}H_{46}N_6O_{13}$<br><br>Monoisotopic mass (Da): 614.3117<br><br>pKa (strongest acidic):<br>12.15<br><br>pKa (strongest basic):<br>9.3<br><br>Partition coefficient (Log P): Index -1 out of bounds for length 42<br><br>Intrinsic solubility (mg mL^{-1}): 0.525 (High)	$[M+H]^+$;<br>$[M+2H]^{2+}$	615.3196;<br>308.1634	455.2348;<br>323.1925;<br>161.0921;<br>203.1026		$[M+2H]^{2+}$<br>161.0921;<br>114.0550;<br>163.1077;<br>125.0709;<br>455.2348

**Group/Class - Pharmacologically active Substance**	**Physicochemical properties**	**Monitored ion**	**Theoretical exact mass MS (*m/z*)**	**Fragment ions (*m/z*) MS^2**		
				$[M+H]^+$	$[M-H]^-$	Others
Tobramycin<br><br>CAS registration number: 32986-56-4<br><br>ChemSpider ID: 33377<br><br>PubChem CID: 36294<br><br>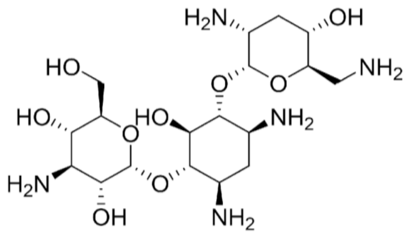	Molecular formula: $C_{18}H_{37}N_5O_9$<br><br>Monoisotopic mass (Da): 467.2586<br><br>pKa (strongest acidic): 12.53<br><br>pKa (strongest basic): 9.42<br><br>Partition coefficient (Log P): -6.478<br><br>Intrinsic solubility (mg mL^{-1}): -0.023 (High)	$[M+H]^+$;<br>$[M-H]^-$	468.2664;<br>466.2519	324.1765;<br>163.1077;<br>145.0972;<br>205.1182	305.1833;<br>234.0986;<br>304.1517;<br>178.0723;<br>287.1238	

Group/Class - Pharmacologically active Substance	Physicochemical properties	Monitored ion	Theoretical exact mass MS (*m/z*)	Fragment ions (*m/z*) MS^2		
				[M+H]$^+$	[M-H]$^-$	Others
**Antimicrobials/Amphenicols**						
Chloramphenicol<br><br>CAS registration number:<br>56-75-7<br><br>ChemSpider ID:<br>5744<br><br>PubChem CID:<br>5959<br><br>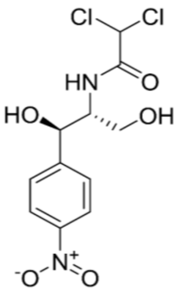	Molecular formula:<br>$C_{11}H_{12}Cl_2N_2O_5$<br><br>Monoisotopic mass (Da): 322.0118<br><br>pKa (strongest acidic): 10.79<br><br>pKa (strongest basic): -<br><br>Partition coefficient (Log P): 0.879<br><br>Intrinsic solubility (mg mL^{-1}): -2.903 (High)	[M+H]$^+$;<br>[M-H]$^-$	323.0196;<br>321.0051	274.9985;<br>165.0659;<br>82.9450;<br>151.0628;<br>305.0090	152.0353;<br>321.0051;<br>121.0293;<br>151.0274;<br>257.0335	

Group/Class - Pharmacologically active Substance	Physicochemical properties	Monitored ion	Theoretical exact mass MS (*m/z*)	Fragment ions (*m/z*) MS^2		
				[M+H]$^+$	[M-H]$^-$	Others
Florfenicol<br><br>CAS registration number: 73231-34-2<br><br>ChemSpider ID: 102776<br><br>PubChem CID: 114811<br><br>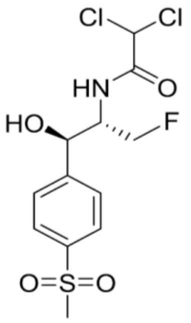	Molecular formula: $C_{12}H_{14}Cl_2FNO_4S$<br><br>Monoisotopic mass (Da): 356.9999<br><br>pKa (strongest acidic): 10.68<br><br>pKa (strongest basic): -<br><br>Partition coefficient (Log P): 0.67<br><br>Intrinsic solubility (mg mL^{-1}): -2.955 (High)	[M+H]$^+$;<br>[M-H]$^-$	358.0077;<br>355.9932	103.0542;<br>319.9907;<br>241.0053;<br>210.0582;<br>206.0366	185.0278;<br>335.9870;<br>119.0499;<br>78.9854;<br>121.0286	

Group/Class - Pharmacologically active Substance	Physicochemical properties	Monitored ion	Theoretical exact mass MS (*m/z*)	Fragment ions (*m/z*) MS^2		
				[M+H]$^+$	[M-H]$^-$	Others
Thiamphenicol<br><br>CAS registration number:<br>15318-45-3<br><br>ChemSpider ID:<br>25315<br><br>PubChem CID:<br>27200<br><br>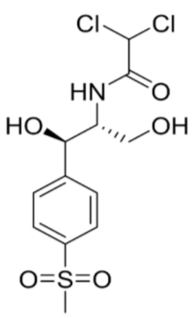	Molecular formula:<br>$C_{12}H_{15}Cl_2NO_5S$<br><br>Monoisotopic mass (Da): 355.0043<br><br>pKa (strongest acidic): 10.95<br><br>pKa (strongest basic): -<br><br>Partition coefficient (Log P): -0.221<br><br>Intrinsic solubility (mg mL^{-1}): -2.266 (High)	[M+H]$^+$;<br>[M-H]$^-$	356.0121;<br>353.9975	307.9909;<br>241.0056;<br>229.0056;<br>119.0729;<br>82.9450	353.9975;<br>185.0278;<br>78.9859;<br>121.0296;<br>119.0504	

Group/Class - Pharmacologically active Substance	Physicochemical properties	Monitored ion	Theoretical exact mass MS (*m/z*)	Fragment ions (*m/z*) MS^2		
				$[M+H]^+$	$[M-H]^-$	Others
**Antimicrobials/Betalactams**						
Amoxicillin<br><br>CAS registration number:<br>26787-78-0<br><br>ChemSpider ID:<br>31006<br><br>PubChem CID:<br>33613<br><br>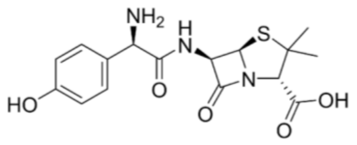	Molecular formula:<br>$C_{16}H_{19}N_3O_5S$<br><br>Monoisotopic mass (Da): 365.1040<br><br>pKa (strongest acidic): 3.23<br><br>pKa (strongest basic): 7.22<br><br>Partition coefficient (Log P): -2.308<br><br>Intrinsic solubility (mg mL^{-1}): -4.35 (Moderate)	$[M+H]^+$;<br>$[M-H]^-$	366.1118;<br>364.0973	349.0853;<br>211.0711;<br>86.0059;<br>107.0491;<br>114.0007	223.0547;<br>206.0281;<br>83.0251;<br>133.0295;<br>123.0451	

Group/Class - Pharmacologically active Substance	Physicochemical properties	Monitored ion	Theoretical exact mass MS (*m/z*)	Fragment ions (*m/z*) MS^2		
				[M+H]$^+$	[M-H]$^-$	Others
Ampicillin<br><br>CAS registration number:<br>69-53-4<br><br>ChemSpider ID:<br>6013<br><br>PubChem CID:<br>6249<br><br>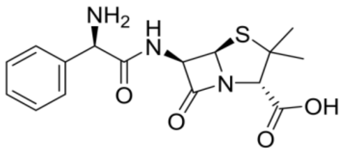	Molecular formula:<br>$C_{16}H_{19}N_3O_4S$<br><br>Monoisotopic mass (Da): 349.1091<br><br>pKa (strongest acidic): 3.24<br><br>pKa (strongest basic): 7.23<br><br>Partition coefficient (Log P): -2.005<br><br>Intrinsic solubility (mg mL^{-1}): -4.789 (Low)	[M+H]$^+$;<br>[M-H]$^-$	350.1169;<br>348.1024	106.0651;<br>160.0427;<br>89.0389;<br>79.0542;<br>95.0491	207.0598;<br>348.1023;<br>74.0070;<br>99.9863;<br>304.1125	

Group/Class - Pharmacologically active Substance	Physicochemical properties	Monitored ion	Theoretical exact mass MS (*m/z*)	Fragment ions (*m/z*) MS^2		
				$[M+H]^+$	$[M-H]^-$	Others
Cephalexin<br><br>CAS registration number:<br>15686-71-2<br><br>ChemSpider ID:<br>25541<br><br>PubChem CID:<br>27447<br><br>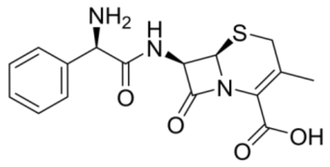	Molecular formula:<br>$C_{16}H_{17}N_3O_4S$<br><br>Monoisotopic mass (Da): 347.0934<br><br>pKa (strongest acidic):<br>3.45<br><br>pKa (strongest basic):<br>7.23<br><br>Partition coefficient (Log P): -2.141<br><br>Intrinsic solubility (mg mL^{-1}): -3.959 (Moderate)	$[M+H]^+$;<br>$[M-H]^-$;<br>$[(M+H_2-CO)+H]^+$;<br>$[M+H+CH_3OH]^+$	348.1013;<br>346.0867;<br>322.1220;<br>380.1275	68.0495;<br>160.0426;<br>158.0270;<br>106.0651	233.0390;<br>70.9835;<br>268.1092;<br>173.0720;<br>107.0615	$[(M+H_2-CO)+H]^+$<br>191.0815;<br>184.9777;<br>106.0651;<br>220.0791;<br>209.0743<br><br>$[M+H+CH_3OH]^+$<br>106.0651;<br>174.0550;<br>151.0866;<br>198.0219

**Group/Class - Pharmacologically active Substance**	**Physicochemical properties**	**Monitored ion**	**Theoretical exact mass MS (*m/z*)**	**Fragment ions (*m/z*) MS^2**		
				$[M+H]^+$	$[M-H]^-$	Others
Cefalonium<br><br>CAS registration number: 5575-21-3<br><br>ChemSpider ID: 20438<br><br>PubChem CID: 21743<br><br>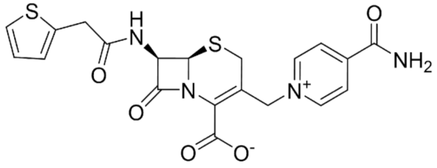	Molecular formula: $C_{20}H_{18}N_4O_5S_2$<br><br>Monoisotopic mass (Da): 458.0713<br><br>pKa (strongest acidic): 3.09<br><br>pKa (strongest basic): -0.86<br><br>Partition coefficient (Log P): -4.243<br><br>Intrinsic solubility (mg mL^{-1}): The molecule cannot be neutralized and the calculation is not defined for molecules with non-zero charge	$[M+H]^+$;<br>$[(M+H_2CO)+H]^+$	459.0791;<br>433.0999	337.0311;<br>152.0165;<br>97.0106;<br>80.0495;<br>108.0682		$[(M+H_2-CO)+H]^+$<br>119.0855;<br>337.0311;<br>311.0512;<br>228.0325;<br>209.0379

Group/Class - Pharmacologically active Substance	Physicochemical properties	Monitored ion	Theoretical exact mass MS (*m/z*)	Fragment ions (*m/z*) MS^2		
				$[M+H]^+$	$[M-H]^-$	Others
Cephapirin<br><br>CAS registration number: 21593-23-7<br><br>ChemSpider ID: -<br><br>PubChem CID: 30699<br><br>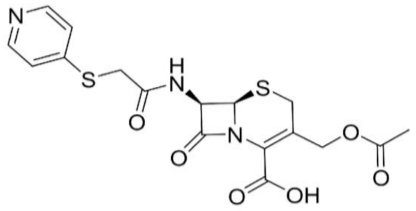	Molecular formula: $C_{17}H_{17}N_3O_6S_2$<br><br>Monoisotopic mass (Da): 423.0553<br><br>pKa (strongest acidic): 3.54<br><br>pKa (strongest basic): 5<br><br>Partition coefficient (Log P): -2.001<br><br>Intrinsic solubility (mg mL^{-1}): -3.723 (High)	$[M+H]^+$	424.0632	292.0573; 181.0431; 152.0166; 141.0482; 112.0216		

**Group/Class - Pharmacologically active Substance**	**Physicochemical properties**	**Monitored ion**	**Theoretical exact mass MS (*m/z*)**	**Fragment ions (*m/z*) MS^2**		
				$[M+H]^+$	$[M-H]^-$	Others
Cefazolin<br><br>CAS registration number:<br>25953-19-9<br><br>ChemSpider ID:<br>30723<br><br>PubChem CID:<br>33255<br><br>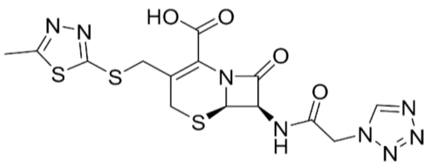	Molecular formula:<br>$C_{14}H_{14}N_8O_4S_3$<br><br>Monoisotopic mass (Da): 454.0295<br><br>pKa (strongest acidic): 3.03<br><br>pKa (strongest basic): 0.26<br><br>Partition coefficient (Log P): -1.519<br><br>Intrinsic solubility (mg mL^{-1}): -4.131 (Moderate)	$[M+H]^+$;<br>$[M-H]^-$	455.0373;<br>453.0227	156.0112;<br>80.0495;<br>323.0557;<br>112.0215;<br>97.0106	167.0285;<br>69.0207;<br>251.0131;<br>130.9743;<br>207.0233	

Group/Class - Pharmacologically active Substance	Physicochemical properties	Monitored ion	Theoretical exact mass MS (*m/z*)	Fragment ions (*m/z*) MS^2		
				[M+H]$^+$	[M-H]$^-$	Others
Cefoperazone<br><br>CAS registration number:<br>62893-19-0<br><br>ChemSpider ID:<br>40206<br><br>PubChem CID:<br>44187<br><br>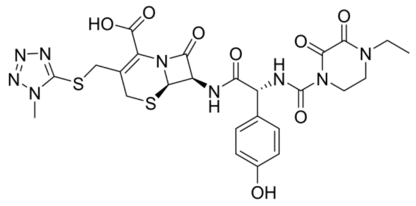	Molecular formula:<br>$C_{25}H_{27}N_9O_8S_2$<br><br>Monoisotopic mass (Da): 645.1419<br><br>pKa (strongest acidic): 3.38<br><br>pKa (strongest basic): -1.98<br><br>Partition coefficient (Log P): -0.9<br><br>Intrinsic solubility (mg mL^{-1}): -5.213 (Low)	[M+H]$^+$;<br>[M-H]$^-$	646.1497;<br>644.1351	530.1340;<br>115.0502;<br>186.0874;<br>156.0114;<br>143.0815	115.0083;<br>117.0345;<br>71.9788;<br>144.0454;<br>89.0397	

**Group/Class - Pharmacologically active Substance**	**Physicochemical properties**	**Monitored ion**	**Theoretical exact mass MS (*m/z*)**	**Fragment ions (*m/z*) MS^2**		
				[M+H]$^+$	[M-H]$^-$	Others
Cefquinome<br><br>CAS registration number: 84957-30-2<br><br>ChemSpider ID: 16736863<br><br>PubChem CID: 5464355<br><br>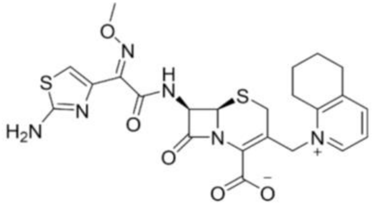	Molecular formula: $C_{23}H_{24}N_6O_5S_2$<br><br>Monoisotopic mass (Da): 528.1244<br><br>pKa (strongest acidic): 2.85<br><br>pKa (strongest basic): 3.61<br><br>Partition coefficient (Log P): -3.531<br><br>Intrinsic solubility (mg mL^{-1}): The molecule cannot be neutralized and the calculation is not defined for molecules with non-zero charge	[M+H]$^+$;<br>[M+2H]$^{2+}$;<br>[(M+H_2CO)+H]$^+$	529.1322;<br>265.0698;<br>503.1530	125.0056;<br>324.0579;<br>167.0272;<br>156.0224;<br>134.0964		[M+2H]$^{2+}$<br>134.0964;<br>199.1230;<br>167.0274;<br>125.0056<br><br>[(M+H_2-CO)+H]$^+$<br>201.0441;<br>134.0964;<br>241.0390;<br>370.0631;<br>126.0120

**Group/Class - Pharmacologically active Substance**	**Physicochemical properties**	**Monitored ion**	**Theoretical exact mass MS (*m/z*)**	**Fragment ions (*m/z*) MS^2**		
				$[M+H]^+$	$[M-H]^-$	Others
Cloxacillin<br><br>CAS registration number:<br>61-72-3<br><br>ChemSpider ID:<br>5873<br><br>PubChem CID:<br>6098<br><br>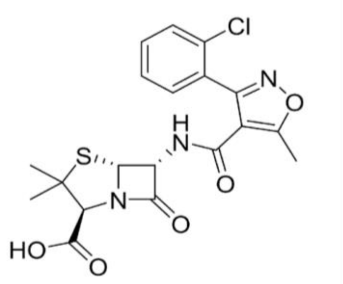	Molecular formula:<br>$C_{19}H_{18}ClN_3O_5S$<br><br>Monoisotopic mass (Da): 435.0650<br><br>pKa (strongest acidic):<br>3.75<br><br>pKa (strongest basic):<br>-0.42<br><br>Partition coefficient (Log P): 2.302<br><br>Intrinsic solubility (mg mL^{-1}): -6.259 (Low)	$[M+H]^+$;<br>$[M-H]^-$;<br>$[M+Na]^+$;<br>$[(M+H_2-CO)+H]^+$;<br>$[M+H+CH_3OH]^+$	436.0729;<br>434.0583;<br>458.0548;<br>410.0936;<br>468.0991	122.9996;<br>87.0263;<br>178.0052;<br>160.0425;<br>114.0371	293.0157;<br>74.9910;<br>65.9986;<br>83.0139;<br>257.0391	$[M+Na]^+$<br>182.0241;<br>299.0189;<br>282.0397;<br>278.0448;<br>271.0240<br><br>$[(M+H_2-CO)+H]^+$<br>174.0583;<br>128.0528;<br>364.0881;<br>100.0393;<br>220.0160<br><br>$[M+H+CH_3OH]^+$<br>160.0427;<br>309.0632;<br>436.0728;<br>232.0636

Group/Class - Pharmacologically active Substance	Physicochemical properties	Monitored ion	Theoretical exact mass MS (*m/z*)	Fragment ions (*m/z*) MS^2		
				$[M+H]^+$	$[M-H]^-$	Others
Dicloxacillin<br><br>CAS registration number:<br>3116-76-5<br><br>ChemSpider ID:<br>17358<br><br>PubChem CID:<br>18381<br><br>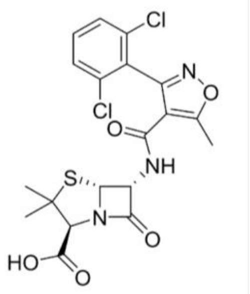 	Molecular formula:<br>$C_{19}H_{17}Cl_2N_3O_5S$<br><br>Monoisotopic mass (Da): 469.0261<br><br>pKa (strongest acidic):<br>3.75<br><br>pKa (strongest basic):<br>-0.71<br><br>Partition coefficient (Log P): 2.906<br><br>Intrinsic solubility ($mg\ mL^{-1}$):<br>-6.893 (Low)	$[M+H]^+$;<br>$[M-H]^-$;<br>$[M+Na]^+$;<br>$[(M+H_2-CO)+H]^+$;<br>$[M+H+CH_3OH]^+$	470.0339;<br>468.0193;<br>492.0158<br>444.0546;<br>502.0601	160.0427;<br>114.0372;<br>87.0263;<br>156.9606;<br>108.9840	326.9767;<br>291.0000;<br>83.0140;<br>65.9986;<br>72.9992	$[M+Na]^+$<br>332.9794;<br>182.0243;<br>316.0007;<br>312.0058<br><br>$[(M+H_2-CO)+H]^+$<br>174.0583;<br>128.0528;<br>312.0301;<br>398.0491;<br>271.0031<br><br>$[M+H+CH_3OH]^+$<br>160.0427;<br>470.0339;<br>343.0242;<br>232.0636

Group/Class - Pharmacologically active Substance	Physicochemical properties	Monitored ion	Theoretical exact mass MS (*m/z*)	Fragment ions (*m/z*) MS^2		
				$[M+H]^+$	$[M-H]^-$	Others
Nafcillin<br><br>CAS registration number:<br>147-52-4<br><br>ChemSpider ID:<br>8634<br><br>PubChem CID:<br>8982<br><br>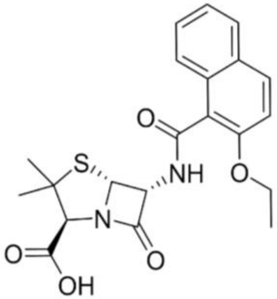	Molecular formula:<br>$C_{21}H_{22}N_2O_5S$<br><br>Monoisotopic mass (Da): 414.1244<br><br>pKa (strongest acidic): 3.31<br><br>pKa (strongest basic): -1.88<br><br>Partition coefficient (Log P): 2.289<br><br>Intrinsic solubility (mg mL^{-1}): -7.052 (Low)	$[M+H]^+$;<br>$[M-H]^-$;<br>$[(M+H_2CO)+H]^+$	415.1322;<br>413.1177;<br>389.1530	199.0754;<br>115.0542;<br>171.0441;<br>143.0491;<br>89.0386	272.0751;<br>243.0359;<br>72.9992;<br>115.0553;<br>70.9835	$[(M+H_2\text{-}CO)+H]^+$<br>199.0754;<br>372.1264;<br>343.1475;<br>115.0542;<br>143.0491

Group/Class - Pharmacologically active Substance	Physicochemical properties	Monitored ion	Theoretical exact mass MS (m/z)	Fragment ions (m/z) MS^2		
				$[M+H]^+$	$[M-H]^-$	Others
Oxacillin<br><br>CAS registration number:<br>66-79-5<br><br>ChemSpider ID:<br>5961<br><br>PubChem CID:<br>6196<br><br>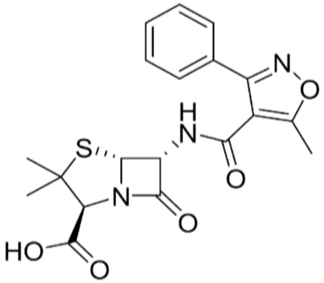	Molecular formula:<br>$C_{19}H_{19}N_3O_5S$<br><br>Monoisotopic mass (Da): 401.1040<br><br>pKa (strongest acidic):<br>3.75<br><br>pKa (strongest basic):<br>-0.12<br><br>Partition coefficient (Log P): 1.698<br><br>Intrinsic solubility (mg mL^{-1}): -5.606 (Low)	$[M+H]^+$;<br>$[M-H]^-$;<br>$[(M+H_2-CO)+H]^+$	402.1118;<br>400.0973;<br>376.1326	160.0427;<br>114.0372;<br>95.0490;<br>243.0764;<br>87.0263	259.0547;<br>74.9910;<br>65.9985;<br>83.0139;<br>70.9835	$[(M+H_2-CO)+H]^+$<br>174.0583;<br>128.0528;<br>330.1271;<br>186.0548;<br>144.0444

Group/Class - Pharmacologically active Substance	Physicochemical properties	Monitored ion	Theoretical exact mass MS (*m/z*)	Fragment ions (*m/z*) MS^2		
				$[M+H]^+$	$[M-H]^-$	Others
Benzylpenicillin<br><br>CAS registration number:<br>61-33-6<br><br>ChemSpider ID:<br>5693<br><br>PubChem CID:<br>5904<br><br>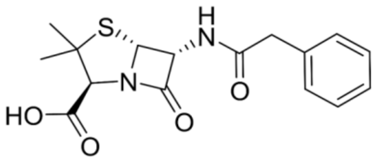	Molecular formula:<br>$C_{16}H_{18}N_2O_4S$<br><br>Monoisotopic mass (Da): 334.0982<br><br>pKa (strongest acidic): 3.53<br><br>pKa (strongest basic): -<br><br>Partition coefficient (Log P): 1.081<br><br>Intrinsic solubility (mg mL^{-1}): -4.555 (Low)	$[M+H]^+$;<br>$[M-H]^-$;<br>$[M+Na]^+$	335.1060;<br>333.0915;<br>357.0880	189.0692;<br>176.0706;<br>160.0427;<br>114.0372;<br>91.05417	192.0489;<br>255.1128;<br>171.0598;<br>289.1017	$[M+Na]^+$<br>181.0733;<br>182.0246;<br>198.0525;<br>239.0791;<br>313.0981

Group/Class - Pharmacologically active Substance	Physicochemical properties	Monitored ion	Theoretical exact mass MS (*m/z*)	Fragment ions (*m/z*) MS^2		
				$[M+H]^+$	$[M-H]^-$	Others
Phenoxymethylpenicillin/Phenoxymethyl V penicillin<br><br>CAS registration number:<br>87-08-1<br><br>ChemSpider ID:<br>6607<br><br>PubChem CID:<br>6869<br><br>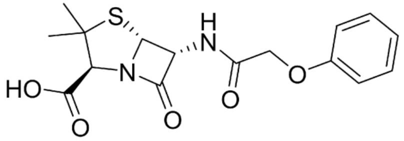	Molecular formula:<br>$C_{16}H_{18}N_2O_5S$<br><br>Monoisotopic mass (Da): 350.0931<br><br>pKa (strongest acidic): 3.39<br><br>pKa (strongest basic): -<br><br>Partition coefficient (Log P): 0.763<br><br>Intrinsic solubility (mg mL^{-1}): -4.957 (Low)	$[M+H]^+$;<br>$[M-H]^-$;<br>$[M+Na]^+$;<br>$[(M+H_2CO)+H]^+$	351.1009;<br>349.0864;<br>373.0829;<br>325.1217	87.0263;<br>160.0425;<br>114.0371;<br>70.0651;<br>105.0447	93.0346;<br>208.0438;<br>114.0019;<br>65.0397;<br>211.0547	$[M+Na]^+$<br>214.0475;<br>182.0243;<br>269.1260;<br>245.0879;<br>303.0563<br><br>$[(M+H_2\text{-}CO)+H]^+$<br>279.1162;<br>193.0972;<br>174.0583;<br>128.0528;<br>100.0393

Group/Class - Pharmacologically active Substance	Physicochemical properties	Monitored ion	Theoretical exact mass MS (*m/z*)	Fragment ions (*m/z*) MS^2		
				[M+H]$^+$	[M-H]$^-$	Others
**Antimicrobials/Lincosamides**						
Clindamycin<br><br>CAS registration number: 13441-63-9<br><br>ChemSpider ID: 393915<br><br>PubChem CID: 446598<br><br>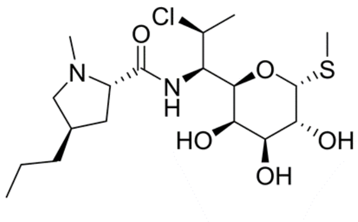	Molecular formula: $C_{18}H_{33}ClN_2O_5S$<br><br>Monoisotopic mass (Da): 424.1793<br><br>pKa (strongest acidic): 12.41<br><br>pKa (strongest basic): 7.55<br><br>Partition coefficient (Log P): 1.038<br><br>Intrinsic solubility (mg mL^{-1}): -3.463 (High)	[M+H]$^+$; [M-H]$^-$	425.1872; 423.1726	126.1277; 377.1838; 389.2105; 407.1766; 299.1965	387.1948; 279.1703; 251.1772; 223.1805; 263.1772	

Group/Class - Pharmacologically active Substance	Physicochemical properties	Monitored ion	Theoretical exact mass MS (*m/z*)	Fragment ions (*m/z*) MS^2		
				[M+H]$^+$	[M-H]$^-$	Others
Lincomycin<br><br>CAS registration number: 154-21-2<br><br>ChemSpider ID: 2272112<br><br>PubChem CID: 3000540<br><br>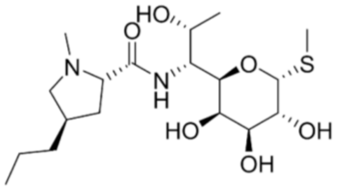	Molecular formula: $C_{18}H_{34}N_2O_6S$<br><br>Monoisotopic mass (Da): 406.2132<br><br>pKa (strongest acidic): 12.37<br><br>pKa (strongest basic): 7.97<br><br>Partition coefficient (Log P): -0.317<br><br>Intrinsic solubility (mg mL^{-1}): -2.386 (High)	[M+H]$^+$; [M-H]$^-$	407.2210; 405.2065	389.2101; 359.2173; 126.1277; 70.0651; 82.0651	405.2065; 169.1346; 127.0401; 71.0139; 85.0295	

Group/Class - Pharmacologically active Substance	Physicochemical properties	Monitored ion	Theoretical exact mass MS (*m/z*)	Fragment ions (*m/z*) MS^2		
				$[M+H]^+$	$[M-H]^-$	Others
**Antimicrobials/Macrolides**						
Azithromycin<br><br>CAS registration number:<br>83905-01-5<br><br>ChemSpider ID:<br>10482163<br><br>PubChem CID:<br>447043<br><br>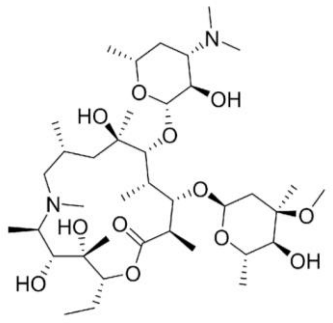	Molecular formula:<br>$C_{38}H_{72}N_2O_{12}$<br><br>Monoisotopic mass (Da): 748.5080<br><br>pKa (strongest acidic): 12.46<br><br>pKa (strongest basic): 11.16<br><br>Partition coefficient (Log P): 2.183<br><br>Intrinsic solubility (mg mL^{-1}): -1.617 (High)	$[M+H]^+$;<br>$[M+2H]^{2+}$	749.5158;<br>375.2615	591.4215;<br>158.1176;<br>116.1070;<br>83.0491;<br>72.0808		$[M+2H]^{2+}$<br>158.1176;<br>591.4215;<br>83.0491;<br>116.1070

Group/Class - Pharmacologically active Substance	Physicochemical properties	Monitored ion	Theoretical exact mass MS (*m/z*)	Fragment ions (*m/z*) MS^2		
				$[M+H]^+$	$[M-H]^-$	Others
Erythromycin (Erythromycin A)<br><br>CAS registration number:<br>114-07-8<br><br>ChemSpider ID:<br>12041<br><br>PubChem CID:<br>12560<br><br>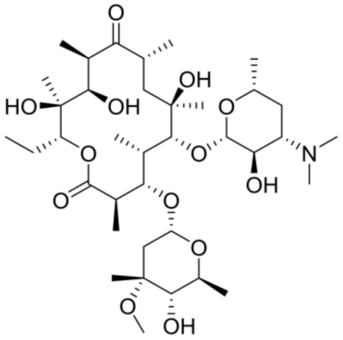	Molecular formula:<br>$C_{37}H_{67}NO_{13}$<br><br>Monoisotopic mass (Da): 733.4607<br><br>pKa (strongest acidic): 12.45<br><br>pKa (strongest basic): 9<br><br>Partition coefficient (Log P): 2.596<br><br>Intrinsic solubility (mg mL^{-1}): -2.273 (High)	$[M+H]^+$;<br>$[M-H_2O+H]^+$	734.4685;<br>716.4580	158.1176;<br>127.0754;<br>116.0706;<br>83.0491;<br>72.0808		$[M-H_2O+H]^+$<br>558.3637;<br>158.1176;<br>540.3525;<br>522.3425;<br>98.0966

Group/Class - Pharmacologically active Substance	Physicochemical properties	Monitored ion	Theoretical exact mass MS (*m/z*)	Fragment ions (*m/z*) MS^2		
				[M+H]$^+$	[M-H]$^-$	Others
Spiramycin (Spiramycin I)<br><br>CAS registration number:<br>8025-81-8<br><br>ChemSpider ID:<br>4451378<br><br>PubChem CID:<br>5356392<br><br>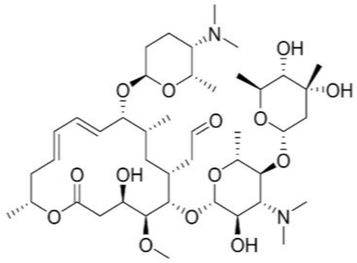	Molecular formula:<br>$C_{43}H_{74}N_2O_{14}$<br><br>Monoisotopic mass (Da): 842.5135<br><br>pKa (strongest acidic): 12.53<br><br>pKa (strongest basic): 9.33<br><br>Partition coefficient (Log P): 2.496<br><br>Intrinsic solubility (mg mL^{-1}): -0.929 (High)	[M+H]$^+$;<br>[M-H]$^-$;<br>[M+2H]$^{2+}$;<br>[M+H+CH_3OH]$^+$	843.5213;<br>841.5067;<br>422.2643;<br>875.5475	174.1125;<br>83.0491;<br>73.0522;<br>101.0597;<br>88.0757	823.4951;<br>488.3007;<br>506.3112;<br>474.2580;<br>334.1860	[M+2H]$^{2+}$<br>101.0597;<br>145.0859;<br>174.1125;<br>142.1226<br><br>[M+H+CH_3OH]$^+$<br>145.0859;<br>174.1125;<br>232.1543;<br>318.1911;<br>142.1226

Group/Class - Pharmacologically active Substance	Physicochemical properties	Monitored ion	Theoretical exact mass MS (*m/z*)	Fragment ions (*m/z*) MS^2		
				[M+H]$^+$	[M-H]$^-$	Others
Tilmicosin<br><br>CAS registration number: 108050-54-0<br><br>ChemSpider ID: 4445656<br><br>PubChem CID: 5282521<br><br>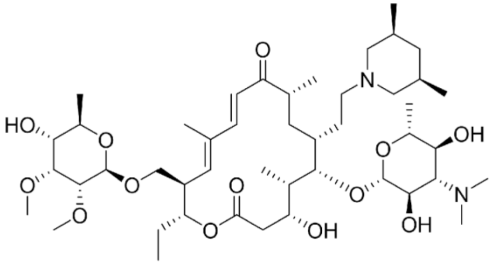	Molecular formula: $C_{46}H_{80}N_2O_{13}$<br><br>Monoisotopic mass (Da): 868.5655<br><br>pKa (strongest acidic): 12.55<br><br>pKa (strongest basic): 10.16<br><br>Partition coefficient (Log P): 4.19<br><br>Intrinsic solubility (mg mL^{-1}): -2.653 (High)	[M+H]$^+$;<br>[M-H]$^-$;<br>[M+2H]$^{2+}$	869.5733;<br>867.5588;<br>435.2903	73.0522;<br>174.1123;<br>132.1017;<br>116.0704;<br>88.07553	116.0717;<br>675.4591;<br>676.4625;<br>222.1863;<br>116.0353	[M+2H]$^{2+}$<br>99.0441;<br>143.0703;<br>340.2482;<br>174.1125

**Group/Class - Pharmacologically active Substance**	**Physicochemical properties**	**Monitored ion**	**Theoretical exact mass MS (*m/z*)**	**Fragment ions (*m/z*) MS^2**		
				[M+H]$^+$	[M-H]$^-$	Others
Tylosin (Tylosin A)<br><br>CAS registration number: 1401-69-0<br><br>ChemSpider ID: 4444097<br><br>PubChem CID: 5280440<br><br>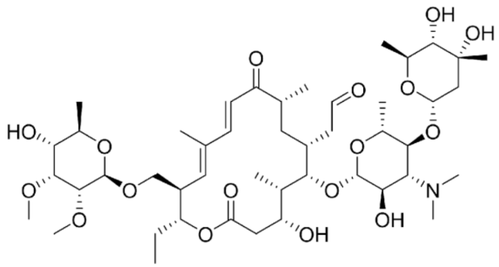	Molecular formula: $C_{46}H_{77}NO_{17}$<br><br>Monoisotopic mass (Da): 915.5186<br><br>pKa (strongest acidic): 12.45<br><br>pKa (strongest basic): 8.43<br><br>Partition coefficient (Log P): 2.316<br><br>Intrinsic solubility (mg mL^{-1}): -1.206 (High)	[M+H]$^+$	916.5264	174.1123; 145.0858; 132.1018; 101.0596; 83.0491		

Group/Class - Pharmacologically active Substance	Physicochemical properties	Monitored ion	Theoretical exact mass MS (*m/z*)	Fragment ions (*m/z*) MS^2		
				$[M+H]^+$	$[M-H]^-$	Others
**Antimicrobials/Quinolones-Fluoroquinolones**						
Nalidixic acid<br><br>CAS registration number:<br>389-08-2<br><br>ChemSpider ID:<br>4268<br><br>PubChem CID:<br>4421<br><br>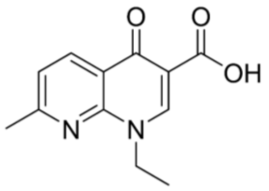	Molecular formula:<br>$C_{12}H_{12}N_2O_3$<br><br>Monoisotopic mass (Da): 232.0842<br><br>pKa (strongest acidic): 5.95<br><br>pKa (strongest basic): 4.68<br><br>Partition coefficient (Log P): 0.873<br><br>Intrinsic solubility (mg mL^{-1}):<br>-2.016 (High)	$[M+H]^+$	233.0921	205.0608;<br>104.0495;<br>159.0553;<br>131.0604;<br>187.0502		

Group/Class - Pharmacologically active Substance	Physicochemical properties	Monitored ion	Theoretical exact mass MS (*m/z*)	Fragment ions (*m/z*) MS^2		
				[M+H]$^+$	[M-H]$^-$	Others
Oxolinic acid<br><br>CAS registration number:<br>14698-29-4<br><br>ChemSpider ID:<br>4467<br><br>PubChem CID:<br>4628<br><br>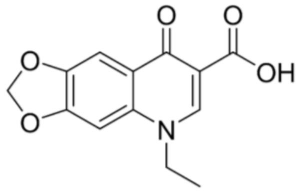	Molecular formula:<br>$C_{13}H_{11}NO_5$<br><br>Monoisotopic mass (Da): 261.0632<br><br>pKa (strongest acidic): 5.58<br><br>pKa (strongest basic): -<br><br>Partition coefficient (Log P): 1.36<br><br>Intrinsic solubility (mg mL^{-1}): -2.338 (High)	[M+H]$^+$	262.0710	244.0602;<br>234.0397;<br>130.0651;<br>160.0393;<br>172.0393		

Group/Class - Pharmacologically active Substance	Physicochemical properties	Monitored ion	Theoretical exact mass MS (*m/z*)	Fragment ions (*m/z*) MS^2		
				[M+H]$^+$	[M-H]$^-$	Others
Ciprofloxacin<br><br>CAS registration number:<br>85721-33-1<br><br>ChemSpider ID:<br>2662<br><br>PubChem CID:<br>2764<br><br>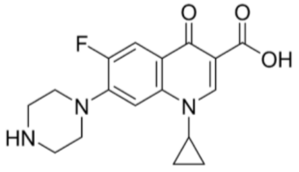	Molecular formula:<br>$C_{17}H_{18}FN_3O_3$<br><br>Monoisotopic mass (Da): 331.1327<br><br>pKa (strongest acidic):<br>5.33<br><br>pKa (strongest basic):<br>8.77<br><br>Partition coefficient (Log P): -0.88<br><br>Intrinsic solubility (mg mL^{-1}): -2.314 (High)	[M+H]$^+$	332.1405	203.0615;<br>288.1503;<br>231.0558;<br>148.0554;<br>188.0376		

Group/Class - Pharmacologically active Substance	Physicochemical properties	Monitored ion	Theoretical exact mass MS (*m/z*)	Fragment ions (*m/z*) MS^2		
				$[M+H]^+$	$[M-H]^-$	Others
Danofloxacin<br><br>CAS registration number:<br>112398-08-0<br><br>ChemSpider ID:<br>64439<br><br>PubChem CID:<br>71335<br><br>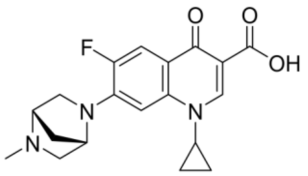	Molecular formula:<br>$C_{19}H_{20}FN_3O_3$<br><br>Monoisotopic mass (Da): 357.1483<br><br>pKa (strongest acidic): 5.26<br><br>pKa (strongest basic): 7.72<br><br>Partition coefficient (Log P): -0.386<br><br>Intrinsic solubility (mg mL^{-1}): -2.639 (High)	$[M+H]^+$	358.1562	82.0651;<br>314.1659;<br>283.1241;<br>255.0564;<br>96.0808		

Group/Class - Pharmacologically active Substance	Physicochemical properties	Monitored ion	Theoretical exact mass MS (*m/z*)	Fragment ions (*m/z*) MS^2		
				[M+H]$^+$	[M-H]$^-$	Others
Difloxacin<br><br>CAS registration number:<br>98106-17-3<br><br>ChemSpider ID:<br>50725<br><br>PubChem CID:<br>56206<br><br>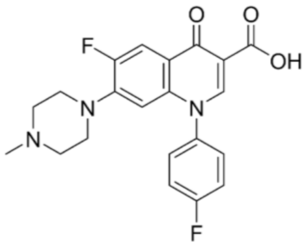	Molecular formula:<br>$C_{21}H_{19}F_2N_3O_3$<br><br>Monoisotopic mass (Da): 399.1389<br><br>pKa (strongest acidic): 5.7<br><br>pKa (strongest basic): 8.44<br><br>Partition coefficient (Log P): 0.709<br><br>Intrinsic solubility (mg mL^{-1}): -3.668 (High)	[M+H]$^+$	400.1467	356.1565;<br>299.0987;<br>227.0538;<br>209.0634;<br>75.0228		

Group/Class - Pharmacologically active Substance	Physicochemical properties	Monitored ion	Theoretical exact mass MS (*m/z*)	Fragment ions (*m/z*) MS^2		
				[M+H]$^+$	[M-H]$^-$	Others
Enrofloxacin<br><br>CAS registration number:<br>93106-60-6<br><br>ChemSpider ID:<br>64326<br><br>PubChem CID:<br>71188<br><br>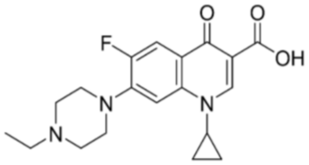	Molecular formula:<br>$C_{19}H_{22}FN_3O_3$<br><br>Monoisotopic mass (Da): 359.1640<br><br>pKa (strongest acidic): 5.32<br><br>pKa (strongest basic): 8.72<br><br>Partition coefficient (Log P): -0.359<br><br>Intrinsic solubility (mg mL^{-1}): -2.415 (High)	[M+H]$^+$;<br>[M-H]$^-$	360.1718;<br>358.1572	316.1816;<br>245.1082;<br>189.0459;<br>203.0613;<br>148.0556	189.0472;<br>314.1674;<br>274.1362;<br>182.0614;<br>220.0779	

**Group/Class - Pharmacologically active Substance**	**Physicochemical properties**	**Monitored ion**	**Theoretical exact mass MS (*m/z*)**	**Fragment ions (*m/z*) MS^2**		
				$[M+H]^+$	$[M-H]^-$	Others
Flumequine<br><br>CAS registration number:<br>42835-25-6<br><br>ChemSpider ID:<br>3257<br><br>PubChem CID:<br>3374<br><br>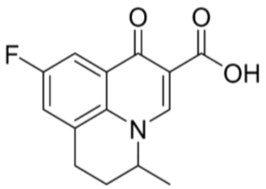	Molecular formula:<br>$C_{14}H_{12}FNO_3$<br><br>Monoisotopic mass (Da): 261.0796<br><br>pKa (strongest acidic): 5.6<br><br>pKa (strongest basic): -<br><br>Partition coefficient (Log P): 2.424<br><br>Intrinsic solubility (mg mL^{-1}): -2.583 (High)	$[M+H]^+$	262.0874	238.0499;<br>98.0152;<br>220.0404;<br>262.0874;<br>99.0077		

Group/Class - Pharmacologically active Substance	Physicochemical properties	Monitored ion	Theoretical exact mass MS (*m/z*)	Fragment ions (*m/z*) MS^2		
				$[M+H]^+$	$[M-H]^-$	Others
Norfloxacin<br><br>CAS registration number:<br>70458-96-7<br><br>ChemSpider ID:<br>4380<br><br>PubChem CID:<br>4539<br><br>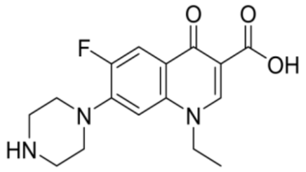	Molecular formula:<br>$C_{16}H_{18}FN_3O_3$<br><br>Monoisotopic mass (Da): 319.1327<br><br>pKa (strongest acidic):<br>5.34<br><br>pKa (strongest basic):<br>8.77<br><br>Partition coefficient (Log P): -0.988<br><br>Intrinsic solubility (mg mL^{-1}): -2.087 (High)	$[M+H]^+$	320.1405	276.1503;<br>256.1440;<br>233.1082;<br>219.0926;<br>205.0770		

Group/Class - Pharmacologically active Substance	Physicochemical properties	Monitored ion	Theoretical exact mass MS (*m/z*)	Fragment ions (*m/z*) MS^2		
				$[M+H]^+$	$[M-H]^-$	Others
Sarafloxacin<br><br>CAS registration number: 98105-99-8<br><br>ChemSpider ID: 50727<br><br>PubChem CID: 56208<br><br>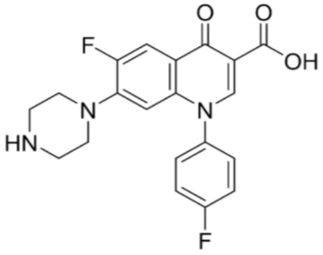 	Molecular formula: $C_{20}H_{17}F_2N_3O_3$<br><br>Monoisotopic mass (Da): 385.1233<br><br>pKa (strongest acidic): 5.7<br><br>pKa (strongest basic): 8.76<br><br>Partition coefficient (Log P): 0.396<br><br>Intrinsic solubility (mg mL^{-1}): -3.891 (Moderate)	$[M+H]^+$	386.1311	342.1411; 299.0989; 285.0832; 255.0726; 227.0539		

Group/Class - Pharmacologically active Substance	Physicochemical properties	Monitored ion	Theoretical exact mass MS (*m/z*)	Fragment ions (*m/z*) MS^2		
				$[M+H]^+$	$[M-H]^-$	Others
**Antimicrobials/Sulphonamides**						
Sulfachlorpyridazine<br><br>CAS registration number:<br>80-32-0<br><br>ChemSpider ID:<br>6382<br><br>PubChem CID:<br>6634<br><br>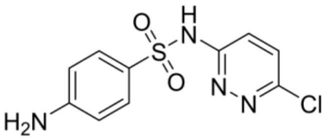	Molecular formula:<br>$C_{20}H_{17}F_2N_3O_3$<br><br>Monoisotopic mass (Da): 385.1233<br><br>pKa (strongest acidic):<br>6.6<br><br>pKa (strongest basic):<br>2.02<br><br>Partition coefficient (Log P): 0.853<br><br>Intrinsic solubility (mg mL^{-1}): -3.786 (Moderate)	$[M+H]^+$	386.1311	342.1411;<br>299.0989;<br>285.0832;<br>255.0726;<br>227.0539		

Group/Class - Pharmacologically active Substance	Physicochemical properties	Monitored ion	Theoretical exact mass MS (*m/z*)	Fragment ions (*m/z*) MS^2		
				$[M+H]^+$	$[M-H]^-$	Others
Sulfadiazine<br><br>CAS registration number:<br>68-35-9<br><br>ChemSpider ID:<br>5026<br><br>PubChem CID:<br>5215<br><br>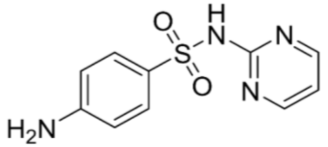	Molecular formula:<br>$C_{10}H_{10}N_4O_2S$<br><br>Monoisotopic mass (Da): 250.0519<br><br>pKa (strongest acidic):<br>6.99<br><br>pKa (strongest basic):<br>2.01<br><br>Partition coefficient (Log P): 0.387<br><br>Intrinsic solubility (mg mL^{-1}): -2.499 (High)	$[M+H]^+$;<br>$[M-H]^-$	251.0597;<br>249.0452	156.0114;<br>108.0444;<br>96.0556;<br>92.0495;<br>65.0386	185.0822;<br>94.0413;<br>93.0335;<br>92.0257;<br>222.0332	

Group/Class - Pharmacologically active Substance	Physicochemical properties	Monitored ion	Theoretical exact mass MS (*m/z*)	Fragment ions (*m/z*) MS^2		
				[M+H]$^+$	[M-H]$^-$	Others
Sulfadimethoxine<br><br>CAS registration number:<br>122-11-2<br><br>ChemSpider ID:<br>5132<br><br>PubChem CID:<br>5323<br><br>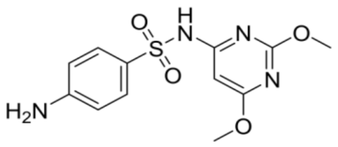	Molecular formula:<br>$C_{12}H_{14}N_4O_4S$<br><br>Monoisotopic mass (Da): 310.0730<br><br>pKa (strongest acidic): 6.91<br><br>pKa (strongest basic): 1.95<br><br>Partition coefficient (Log P): 1.261<br><br>Intrinsic solubility (mg mL^{-1}): -3.599 (High)	[M+H]$^+$;<br>[M-H]$^-$	311.0809;<br>309.0663	245.1032;<br>236.0334;<br>156.0768;<br>108.0443;<br>92.0495	309.0652;<br>66.0097;<br>65.0144;<br>122.0359;<br>154.0621	

Group/Class - Pharmacologically active Substance	Physicochemical properties	Monitored ion	Theoretical exact mass MS (*m/z*)	Fragment ions (*m/z*) MS^2		
				[M+H]$^+$	[M-H]$^-$	Others
Sulfadoxine<br><br>CAS registration number:<br>2447-57-6<br><br>ChemSpider ID:<br>16218<br><br>PubChem CID:<br>17134<br><br>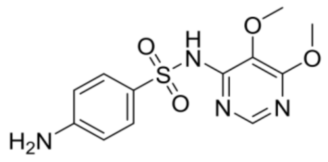	Molecular formula:<br>$C_{12}H_{14}N_4O_4S$<br><br>Monoisotopic mass (Da): 310.0730<br><br>pKa (strongest acidic): 6.12<br><br>pKa (strongest basic): 2.41<br><br>Partition coefficient (Log P): 0.582<br><br>Intrinsic solubility (mg mL^{-1}): -3.115 (High)	[M+H]$^+$;<br>[M-H]$^-$	311.0809;<br>309.0663	156.0113;<br>108.0443;<br>92.0495;<br>65.0380;<br>140.0448	309.0661;<br>156.0127;<br>294.0428;<br>279.0193;<br>215.0576	

Group/Class - Pharmacologically active Substance	Physicochemical properties	Monitored ion	Theoretical exact mass MS (*m/z*)	Fragment ions (*m/z*) MS^2		
				[M+H]$^+$	[M-H]$^-$	Others
Sulfisoxazole/Sulfafurazole<br><br>CAS registration number: 127-69-5<br><br>ChemSpider ID: 5151<br><br>PubChem CID: 5344<br><br>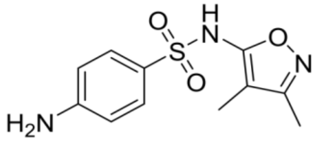	Molecular formula: $C_{11}H_{13}N_3O_3S$<br><br>Monoisotopic mass (Da): 267.0672<br><br>pKa (strongest acidic): 4.81<br><br>pKa (strongest basic): 2.17<br><br>Partition coefficient (Log P): 0.731<br><br>Intrinsic solubility (mg mL^{-1}): -2.392 (High)	[M+H]$^+$;<br>[M-H]$^-$	268.0750;<br>266.0605	268.0748;<br>156.0113;<br>113.0712;<br>108.0447;<br>92.0495	266.0605;<br>171.0233;<br>63.9223;<br>239.0496;<br>79.9811	

**Group/Class - Pharmacologically active Substance**	**Physicochemical properties**	**Monitored ion**	**Theoretical exact mass MS (*m/z*)**	**Fragment ions (*m/z*) MS^2**		
				$[M+H]^+$	$[M-H]^-$	Others
Sulfamerazine<br><br>CAS registration number:<br>127-79-7<br><br>ChemSpider ID:<br>5134<br><br>PubChem CID:<br>5325<br><br>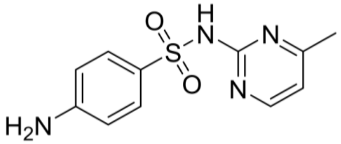	Molecular formula:<br>$C_{11}H_{12}N_4O_2S$<br><br>Monoisotopic mass (Da): 264.0676<br><br>pKa (strongest acidic): 6.99<br><br>pKa (strongest basic): 2<br><br>Partition coefficient (Log P): 0.519<br><br>Intrinsic solubility (mg mL^{-1}): -2.66 (High)	$[M+H]^+$;<br>$[M-H]^-$	265.0754;<br>263.0608	190.0281;<br>156.0113;<br>110.0712;<br>108.0443;<br>92.0495	199.0978;<br>108.0570;<br>92.0257;<br>132.0570;<br>106.0413	

Group/Class - Pharmacologically active Substance	Physicochemical properties	Monitored ion	Theoretical exact mass MS (*m/z*)	Fragment ions (*m/z*) MS^2		
				$[M+H]^+$	$[M-H]^-$	Others
Sulfamethazine/Sulfadimidine<br><br>CAS registration number: 57-68-1<br><br>ChemSpider ID: -<br><br>PubChem CID: 5327<br><br>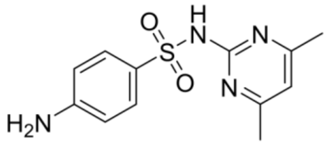	Molecular formula: $C_{12}H_{14}N_4O_2S$<br><br>Monoisotopic mass (Da): 278.0832<br><br>pKa (strongest acidic): 6.99<br><br>pKa (strongest basic): 2<br><br>Partition coefficient (Log P): 0.65<br><br>Intrinsic solubility (mg mL^{-1}): -2.818 (High)	$[M+H]^+$;<br>$[M-H]^-$	279.0910;<br>277.0765	124.0869;<br>92.0495;<br>108.0444;<br>186.0332;<br>156.0114	106.0413;<br>196.0175;<br>122.0726;<br>132.0570;<br>236.0488	

Group/Class - Pharmacologically active Substance	Physicochemical properties	Monitored ion	Theoretical exact mass MS (*m/z*)	Fragment ions (*m/z*) MS^2		
				$[M+H]^+$	$[M-H]^-$	Others
Sulfathiazole<br><br>CAS registration number:<br>72-14-0<br><br>ChemSpider ID:<br>5148<br><br>PubChem CID:<br>5340<br><br>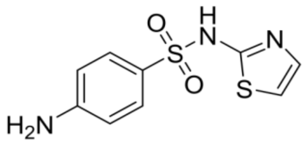	Molecular formula:<br>$C_9H_9N_3O_2S_2$<br><br>Monoisotopic mass (Da): 255.0131<br><br>pKa (strongest acidic):<br>5.73<br><br>pKa (strongest basic):<br>2.04<br><br>Partition coefficient (Log P): 0.975<br><br>Intrinsic solubility (mg mL^{-1}): -2.669 (High)	$[M+H]^+$;<br>$[M-H]^-$	256.0209;<br>254.0063	156.0114;<br>108.0444;<br>101.0168;<br>92.0495;<br>68.0495	156.0125;<br>254.0063;<br>97.9944;<br>63.9624;<br>65.0145	

**Group/Class - Pharmacologically active Substance**	**Physicochemical properties**	**Monitored ion**	**Theoretical exact mass MS (*m/z*)**	**Fragment ions (*m/z*) MS^2**		
				[M+H]$^+$	[M-H]$^-$	Others
Sulfamethoxazole<br><br>CAS registration number:<br>723-46-6<br><br>ChemSpider ID:<br>5138<br><br>PubChem CID:<br>5329<br><br>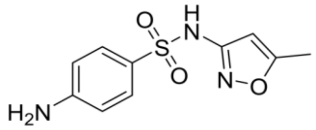	Molecular formula:<br>$C_{10}H_{11}N_3O_3S$<br><br>Monoisotopic mass (Da): 253.0516<br><br>pKa (strongest acidic): 5.86<br><br>pKa (strongest basic): 1.97<br><br>Partition coefficient (Log P): 0.791<br><br>Intrinsic solubility (mg mL^{-1}): -2.202 (High)	[M+H]$^+$;<br>[M-H]$^-$	254.0594;<br>252.0448	156.0113;<br>147.0791;<br>108.0443;<br>99.0553;<br>93.0573	156.0124;<br>252.0449;<br>79.9574;<br>92.0505;<br>108.0454	

Group/Class - Pharmacologically active Substance	Physicochemical properties	Monitored ion	Theoretical exact mass MS (*m/z*)	Fragment ions (*m/z*) MS^2		
				$[M+H]^+$	$[M-H]^-$	Others
Sulfaquinoxaline<br><br>CAS registration number:<br>59-40-5<br><br>ChemSpider ID:<br>5147<br><br>PubChem CID:<br>5338<br><br>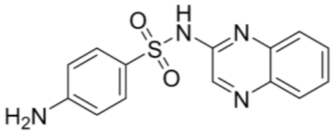	Molecular formula:<br>$C_{14}H_{12}N_4O_2S$<br><br>Monoisotopic mass (Da): 300.0676<br><br>pKa (strongest acidic): 6.79<br><br>pKa (strongest basic): 2.13<br><br>Partition coefficient (Log P): 1.552<br><br>Intrinsic solubility (mg mL^{-1}): -2.917 (High)	$[M+H]^+$;<br>$[M-H]^-$	301.0754;<br>299.0608	156.0112;<br>146.0712;<br>108.0443;<br>80.0495;<br>92.0495	299.0595;<br>144.0565;<br>142.0409;<br>117.0458;<br>115.0300	

Group/Class - Pharmacologically active Substance	Physicochemical properties	Monitored ion	Theoretical exact mass MS (*m/z*)	Fragment ions (*m/z*) MS^2		
				$[M+H]^+$	$[M-H]^-$	Others
**Antimicrobials/Tetracyclines**						
Chlortetracycline<br><br>CAS registration number:<br>57-62-5<br><br>ChemSpider ID:<br>10469370<br><br>PubChem CID:<br>54675777<br><br>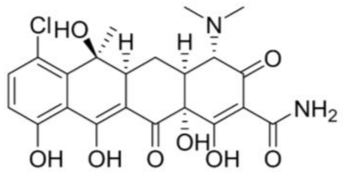 	Molecular formula: $C_{22}H_{23}ClN_2O_8$<br><br>Monoisotopic mass (Da): 478.1137<br><br>pKa (strongest acidic): 2.98<br><br>pKa (strongest basic): 8.88<br><br>Partition coefficient (Log P): -2.9<br><br>Intrinsic solubility (mg mL^{-1}): -2.958 (High)	$[M+H]^+$;<br>$[M-H]^-$	479.1216;<br>477.1070	462.0936;<br>444.0832;<br>154.0495;<br>98.0603;<br>260.0230	392.0905;<br>477.1070;<br>169.0062;<br>65.9986;<br>194.9854	

Group/Class - Pharmacologically active Substance	Physicochemical properties	Monitored ion	Theoretical exact mass MS (*m/z*)	Fragment ions (*m/z*) MS^2		
				[M+H]$^+$	[M-H]$^-$	Others
Doxycycline<br><br>CAS registration number: 564-25-0<br><br>ChemSpider ID: 10469369<br><br>PubChem CID: 54671203<br><br>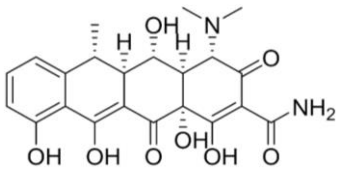	Molecular formula: $C_{22}H_{24}N_2O_8$<br><br>Monoisotopic mass (Da): 444.1527<br><br>pKa (strongest acidic): 7.45<br><br>pKa (strongest basic): 6<br><br>Partition coefficient (Log P): -0.611<br><br>Intrinsic solubility (mg mL^{-1}): -2.746 (High)	[M+H]$^+$; [M-H]$^-$	445.1605; 443.1460	428.1340; 410.1233; 98.0600; 168.0570; 154.0498	443.1460; 240.0428; 358.1296; 255.0663; 211.0411	

Group/Class - Pharmacologically active Substance	Physicochemical properties	Monitored ion	Theoretical exact mass MS (*m/z*)	Fragment ions (*m/z*) MS^2		
				[M+H]$^+$	[M-H]$^-$	Others
Oxytetracycline<br><br>CAS registration number: 79-57-2<br><br>ChemSpider ID: 10482174<br><br>PubChem CID: 54675779<br><br>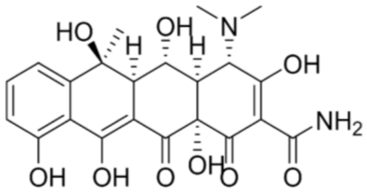	Molecular formula: $C_{22}H_{24}N_2O_9$<br><br>Monoisotopic mass (Da): 460.1476<br><br>pKa (strongest acidic): 3.18<br><br>pKa (strongest basic): 8.65<br><br>Partition coefficient (Log P): -4.569<br><br>Intrinsic solubility (mg mL^{-1}): -1.93 (High)	[M+H]$^+$; [M-H]$^-$	461.1555; 459.1409	426.1183; 201.0546; 212.0648; 184.0519; 145.0648	459.1409; 374.1245; 135.0452; 171.0452; 65.9985	

Group/Class - Pharmacologically active Substance	Physicochemical properties	Monitored ion	Theoretical exact mass MS (*m/z*)	Fragment ions (*m/z*) MS^2		
				[M+H]$^+$	[M-H]$^-$	Others
Tetracycline<br><br>CAS registration number: 60-54-8<br><br>ChemSpider ID: 10257122<br><br>PubChem CID: 54675776<br><br>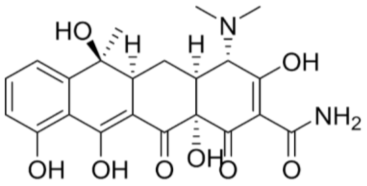	Molecular formula: $C_{22}H_{24}N_2O_8$<br><br>Monoisotopic mass (Da): 444.1527<br><br>pKa (strongest acidic): 3.25<br><br>pKa (strongest basic): 8.96<br><br>Partition coefficient (Log P): -3.499<br><br>Intrinsic solubility (mg mL^{-1}): -2.329 (High)	[M+H]$^+$; [M-H]$^-$	445.1605; 443.1460	428.1336; 410.1229; 154.0497; 445.1605; 98.0600	135.0452; 161.0244; 142.0146; 187.0765; 125.0720	

**Group/Class - Pharmacologically active Substance**	**Physicochemical properties**	**Monitored ion**	**Theoretical exact mass MS (*m/z*)**	**Fragment ions (*m/z*) MS^2**		
				[M+H]$^+$	[M-H]$^-$	Others
**Antimicrobials/Others**						
Bromhexine<br><br>CAS registration number: 3572-43-8<br><br>ChemSpider ID: 2348<br><br>PubChem CID: 2442<br><br>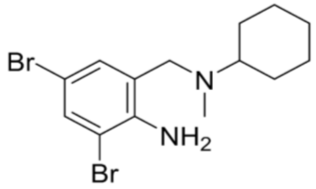	Molecular formula: $C_{14}H_{20}Br_2N_2$<br><br>Monoisotopic mass (Da): 373.9988<br><br>pKa (strongest acidic): -<br><br>pKa (strongest basic): 9.23<br><br>Partition coefficient (Log P): 4.422<br><br>Intrinsic solubility (mg mL^{-1}): -4.685 (Low)	[M+H]$^+$	375.0066	375.0066; 261.8862; 114.1277; 104.0495; 95.0491		

**Group/Class - Pharmacologically active Substance**	**Physicochemical properties**	**Monitored ion**	**Theoretical exact mass MS (*m/z*)**	**Fragment ions (*m/z*) MS^2**		
				$[M+H]^+$	$[M-H]^-$	Others
Dapsone<br><br>CAS registration number:<br>80-08-0<br><br>ChemSpider ID:<br>2849<br><br>PubChem CID:<br>2955<br><br>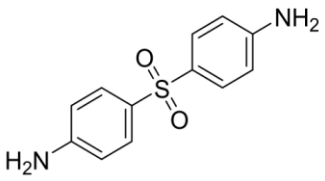	Molecular formula:<br>$C_{12}H_{12}N_2O_2S$<br><br>Monoisotopic mass (Da): 248.0614<br><br>pKa (strongest acidic): -<br><br>pKa (strongest basic): 2.39<br><br>Partition coefficient (Log P): 1.27<br><br>Intrinsic solubility (mg mL^{-1}): -2.91 (High)	$[M+H]^+$;<br>$[M-H]^-$	249.0692<br>247.0547	156.0114;<br>108.0444;<br>65.0386;<br>92.0495;<br>80.0495	79.9574;<br>97.0659;<br>107.0377;<br>121.0295;<br>95.0502	

**Group/Class - Pharmacologically active Substance**	**Physicochemical properties**	**Monitored ion**	**Theoretical exact mass MS (*m/z*)**	**Fragment ions (*m/z*) MS^2**		
				$[M+H]^+$	$[M-H]^-$	Others
Rifampicin<br><br>CAS registration number:<br>13292-46-1<br><br>ChemSpider ID:<br>10468813<br><br>PubChem CID:<br>135900090<br><br>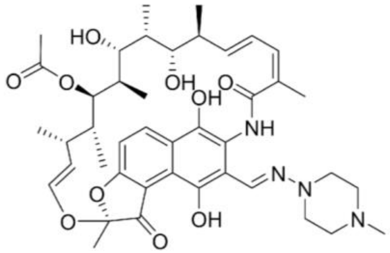	Molecular formula:<br>$C_{43}H_{58}N_4O_{12}$<br><br>Monoisotopic mass (Da): 822.4046<br><br>pKa (strongest acidic): 6.94<br><br>pKa (strongest basic): 7.53<br><br>Partition coefficient (Log P): 2.833<br><br>Intrinsic solubility (mg mL^{-1}): -5.484 (Low)	$[M+H]^+$;<br>$[M-H]^-$	823.4124;<br>821.3979	91.0542;<br>95.0855;<br>791.3862;<br>151.0754;<br>105.0699	297.0517;<br>270.0408;<br>397.1514;<br>65.9985;<br>298.0595	

Group/Class - Pharmacologically active Substance	Physicochemical properties	Monitored ion	Theoretical exact mass MS (*m/z*)	Fragment ions (*m/z*) MS^2		
				$[M+H]^+$	$[M-H]^-$	Others
Tiamulin<br><br>CAS registration number:<br>55297-95-5<br><br>ChemSpider ID:<br>571196<br><br>PubChem CID:<br>656958<br><br>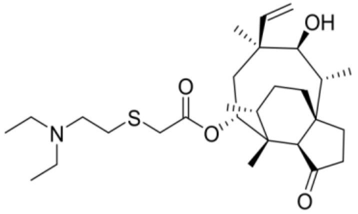	Molecular formula:<br>$C_{28}H_{47}NO_4S$<br><br>Monoisotopic mass (Da): 493.3220<br><br>pKa (strongest acidic): 14.43<br><br>pKa (strongest basic): 9.51<br><br>Partition coefficient (Log P): 4.501<br><br>Intrinsic solubility ($mg\ mL^{-1}$): -4.309 (Moderate)	$[M+H]^+$	494.3299	192.1053;<br>119.0161;<br>95.0491;<br>91.0542;<br>73.0106		

Group/Class - Pharmacologically active Substance	Physicochemical properties	Monitored ion	Theoretical exact mass MS (*m/z*)	Fragment ions (*m/z*) MS^2		
				[M+H]$^+$	[M-H]$^-$	Others
**Antimicrobials Prospect/Quinolones-Fluoroquinolones**						
Marbofloxacin<br><br>CAS registration number: 115550-35-1<br><br>ChemSpider ID: 54663<br><br>PubChem CID: 60651<br><br>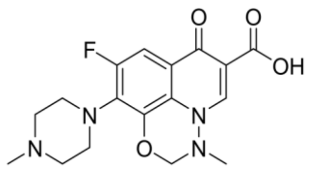	Molecular formula: $C_{17}H_{19}FN_4O_4$<br><br>Monoisotopic mass (Da): 362.1385<br><br>pKa (strongest acidic): 5.1<br><br>pKa (strongest basic): 8.38<br><br>Partition coefficient (Log P): -1.887<br><br>Intrinsic solubility (mg mL^{-1}): -2.06 (High)	[M+H]$^+$	363.1463	320.1036; 277.0615; 205.0404; 72.0807; 70.0651		

Group/Class - Pharmacologically active Substance	Physicochemical properties	Monitored ion	Theoretical exact mass MS (*m/z*)	Fragment ions (*m/z*) MS²		
				[M+H]⁺	[M-H]⁻	Others
**Antimicrobials Prospect/Macrolides**						
Leucomycin/Leucomycin A₅<br><br>CAS registration number: 18361-45-0<br><br>ChemSpider ID: 21250867<br><br>PubChem CID: 5282324<br><br>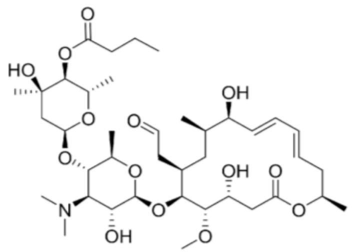	Molecular formula: $C_{39}H_{65}NO_{14}$<br><br>Monoisotopic mass (Da): 771.4400<br><br>pKa (strongest acidic): 12.68<br><br>pKa (strongest basic): 8.51<br><br>Partition coefficient (Log P): 2.488<br><br>Intrinsic solubility (mg mL^{-1}): -2.201 (High)	[M+H]⁺	772.4478	174.1125; 109.0648; 558.3273; 215.1278; 132.1019		

Group/Class - Pharmacologically active Substance	Physicochemical properties	Monitored ion	Theoretical exact mass MS (*m/z*)	Fragment ions (*m/z*) MS^2		
				$[M+H]^+$	$[M-H]^-$	Others
Josamycin/Leucomycin A$_3$<br><br>CAS registration number:<br>16846-24-5<br><br>ChemSpider ID:<br>4445361<br><br>PubChem CID:<br>5282165<br><br>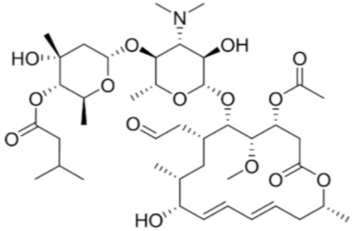	Molecular formula:<br>$C_{42}H_{69}NO_{15}$<br><br>Monoisotopic mass (Da): 827.4662<br><br>pKa (strongest acidic): 12.71<br><br>pKa (strongest basic): 8.51<br><br>Partition coefficient (Log P): 3.216<br><br>Intrinsic solubility (mg mL^{-1}): -2.608 (High)	$[M+H]^+$	828.4740	174.1125;<br>109.0648;<br>83.0491;<br>79.0543;<br>81.0699		

Group/Class - Pharmacologically active Substance	Physicochemical properties	Monitored ion	Theoretical exact mass MS (*m/z*)	Fragment ions (*m/z*) MS^2		
				[M+H]$^+$	[M-H]$^-$	Others
Tildipirosin<br><br>CAS registration number: 328898-40-4<br><br>ChemSpider ID: 30790722<br><br>PubChem CID: 24860548<br><br>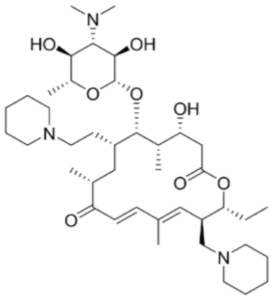	Molecular formula: $C_{41}H_{71}N_3O_8$<br><br>Monoisotopic mass (Da): 733.5236<br><br>pKa (strongest acidic): 12.68<br><br>pKa (strongest basic): 10.05<br><br>Partition coefficient (Log P): 4.457<br><br>Intrinsic solubility (mg mL^{-1}): -2.731 (High)	[M+H]$^+$;<br>[M+3H]$^{3+}$	734.5314;<br>245.5153	561.4262;<br>98.0964;<br>73.0522;<br>70.0651;<br>116.0706		[M+3H]$^{3+}$<br>281.2138;<br>174.1125;<br>98.0964;<br>88.0757

Group/Class - Pharmacologically active Substance	Physicochemical properties	Monitored ion	Theoretical exact mass MS (*m/z*)	Fragment ions (*m/z*) MS^2		
				[M+H]$^+$	[M-H]$^-$	Others
Tulathromycin (Tulathromycin A)<br><br>CAS registration number: 217500-96-4<br><br>ChemSpider ID: 8008030<br><br>PubChem CID: 9832301<br><br>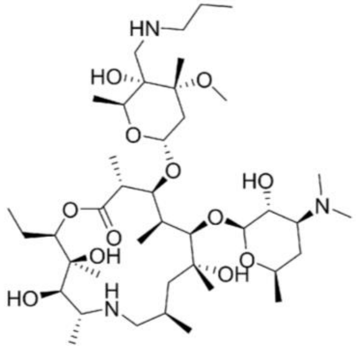	Molecular formula: $C_{41}H_{79}N_3O_{12}$<br><br>Monoisotopic mass (Da): 805.5658<br><br>pKa (strongest acidic): 12.2<br><br>pKa (strongest basic): 10.21<br><br>Partition coefficient (Log P): 2.5<br><br>Intrinsic solubility (mg mL^{-1}): -1.691 (High)	[M+H]$^+$;<br>[M+2H]$^{2+}$;<br>[M+3H]$^{3+}$	806.5737;<br>403.7905;<br>269.5294	577.4059;<br>72.0808;<br>158.1176;<br>116.1073;<br>98.0964		[M+2H]$^{2+}$<br>230.1751;<br>158.1176;<br>116.1070;<br>72.0808<br><br>[M+3H]$^{3+}$<br>158.1176;<br>72.0808;<br>116.0706

Group/Class - Pharmacologically active Substance	Physicochemical properties	Monitored ion	Theoretical exact mass MS (*m/z*)	Fragment ions (*m/z*) MS^2		
				[M+H]$^+$	[M-H]$^-$	Others
**Antimicrobials Prospect/ Sulfonamides**						
Phthalylsulfathiazole<br><br>CAS registration number: 85-73-4<br><br>ChemSpider ID: 4641<br><br>PubChem CID: 4806<br><br>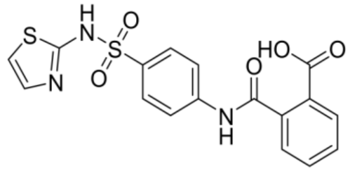	Molecular formula: $C_{17}H_{13}N_3O_5S_2$<br><br>Monoisotopic mass (Da): 403.0291<br><br>pKa (strongest acidic): 4.38<br><br>pKa (strongest basic): 0.59<br><br>Partition coefficient (Log P): 2.553<br><br>Intrinsic solubility (mg mL^{-1}): -4.801 (Low)	[M+H]$^+$; [M-H]$^-$	404.0369; 402.0224	149.0233; 256.0209; 156.0113; 108.0443; 92.0493	358.0315; 254.0066; 196.0767; 190.0447; 260.0376	

Group/Class - Pharmacologically active Substance	Physicochemical properties	Monitored ion	Theoretical exact mass MS (*m/z*)	Fragment ions (*m/z*) MS^2		
				$[M+H]^+$	$[M-H]^-$	Others
Sulfacetamide/N-Sulfanilylacetamide<br><br>CAS registration number:<br>144-80-9<br><br>ChemSpider ID:<br>5129<br><br>PubChem CID:<br>5320<br><br>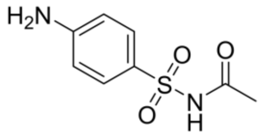	Molecular formula:<br>$C_8H_{10}N_2O_3S$<br><br>Monoisotopic mass (Da): 214.0407<br><br>pKa (strongest acidic):<br>5.6<br><br>pKa (strongest basic):<br>2.14<br><br>Partition coefficient (Log P): -0.261<br><br>Intrinsic solubility (mg mL^{-1}): -1.51 (High)	$[M+H]^+$;<br>$[M-H]^-$	215.0485;<br>213.0339	215.1252;<br>156.0112;<br>108.0443;<br>92.0493;<br>68.0495	195.0223;<br>171.0223	

Group/Class - Pharmacologically active Substance	Physicochemical properties	Monitored ion	Theoretical exact mass MS (*m/z*)	Fragment ions (*m/z*) MS^2		
				[M+H]$^+$	[M-H]$^-$	Others
Sulfamethoxypyridazine<br><br>CAS registration number:<br>80-35-3<br><br>ChemSpider ID:<br>5139<br><br>PubChem CID:<br>5330<br><br>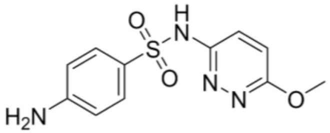	Molecular formula:<br>$C_{11}H_{12}N_4O_3S$<br><br>Monoisotopic mass (Da): 280.0625<br><br>pKa (strongest acidic): 6.84<br><br>pKa (strongest basic): 2.02<br><br>Partition coefficient (Log P): 0.466<br><br>Intrinsic solubility (mg mL^{-1}):<br>-2.908 (High)	[M+H]$^+$;<br>[M-H]$^-$	281.0703;<br>279.0557	156.0113;<br>126.0662;<br>108.0443;<br>92.0495;<br>126.0662	156.0123;<br>279.0551;<br>79.9574;<br>92.0505;<br>264.0322	

Group/Class - Pharmacologically active Substance	Physicochemical properties	Monitored ion	Theoretical exact mass MS (*m/z*)	Fragment ions (*m/z*) MS^2		
				$[M+H]^+$	$[M-H]^-$	Others
**Antimicrobials Prospect/Others**						
Diminazene<br><br>CAS registration number:<br>536-71-0<br><br>ChemSpider ID:<br>2264<br><br>PubChem CID:<br>2354<br><br>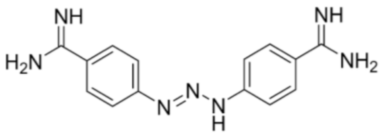	Molecular formula:<br>$C_{14}H_{15}N_7$<br><br>Monoisotopic mass (Da): 281.1384<br><br>pKa (strongest acidic): -<br><br>pKa (strongest basic): 12.07<br><br>Partition coefficient (Log P): 1.763<br><br>Intrinsic solubility (mg mL^{-1}): -4.181 (Moderate)	$[M+H]^+$;<br>$[M-H]^-$	282.1462;<br>280.1316	119.0604;<br>237.1135	252.1244;<br>235.0978;<br>193.0760;<br>210.1026;<br>218.0713	

Group/Class - Pharmacologically active Substance	Physicochemical properties	Monitored ion	Theoretical exact mass MS (*m/z*)	Fragment ions (*m/z*) MS^2		
				[M+H]$^+$	[M-H]$^-$	Others
Fosfomycin<br><br>CAS registration number:<br>23155-02-4<br><br>ChemSpider ID:<br>394204<br><br>PubChem CID:<br>446987<br><br>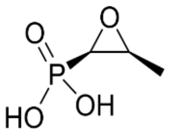	Molecular formula:<br>$C_3H_7O_4P$<br><br>Monoisotopic mass (Da): 138.0077<br><br>pKa (strongest acidic): 1.25<br><br>pKa (strongest basic): -<br><br>Partition coefficient (Log P): -0.738<br><br>Intrinsic solubility (mg mL^{-1}): 1.629 (High)	[M+H]$^+$;<br>[M+Ca]$^+$	139.0155;<br>178.9781	98.9842;<br>121.0049;<br>82.9893;<br>109.0049		[M+Ca]$^+$<br>98.9842;<br>121.0049;<br>82.9893;<br>109.0049

Group/Class - Pharmacologically active Substance	Physicochemical properties	Monitored ion	Theoretical exact mass MS (*m/z*)	Fragment ions (*m/z*) MS^2		
				$[M+H]^+$	$[M-H]^-$	Others
Isoniazid<br><br>CAS registration number:<br>54-85-3<br><br>ChemSpider ID:<br>3635<br><br>PubChem CID:<br>3767<br><br>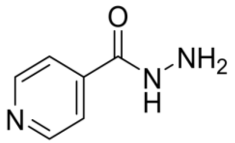	Molecular formula:<br>$C_6H_7N_3O$<br><br>Monoisotopic mass (Da): 137.0584<br><br>pKa (strongest acidic): 13.61<br><br>pKa (strongest basic): 3.26<br><br>Partition coefficient (Log P): -0.69<br><br>Intrinsic solubility (mg mL^{-1}): -0.41 (High)	$[M+H]^+$	138.0662	121.0397;<br>109.0524;<br>138.0662;<br>79.0417;<br>78.0338		

**Group/Class - Pharmacologically active Substance**	**Physicochemical properties**	**Monitored ion**	**Theoretical exact mass MS (*m/z*)**	**Fragment ions (*m/z*) MS^2**		
				[M+H]$^+$	[M-H]$^-$	Others
Novobiocin<br><br>CAS registration number: 303-81-1<br><br>ChemSpider ID: 10226117<br><br>PubChem CID: 54675769<br><br>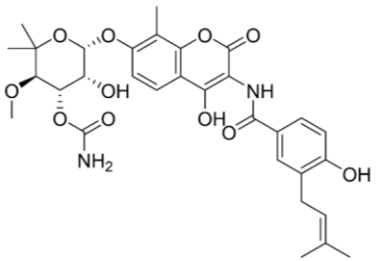	Molecular formula: $C_{31}H_{36}N_2O_{11}$<br><br>Monoisotopic mass (Da): 612.2314<br><br>pKa (strongest acidic): 5.51<br><br>pKa (strongest basic): -<br><br>Partition coefficient (Log P): 3.409<br><br>Intrinsic solubility (mg mL^{-1}): -5.919 (Low)	[M+H]$^+$	613.2392	189.0910; 218.1023; 77.0386; 151.0390; 133.0284		

Group/Class - Pharmacologically active Substance	Physicochemical properties	Monitored ion	Theoretical exact mass MS (*m/z*)	Fragment ions (*m/z*) MS^2		
				[M+H]$^+$	[M-H]$^-$	Others
Rifaximin<br><br>CAS registration number: 80621-81-4<br><br>ChemSpider ID: 10482302<br><br>PubChem CID: 6436173<br><br>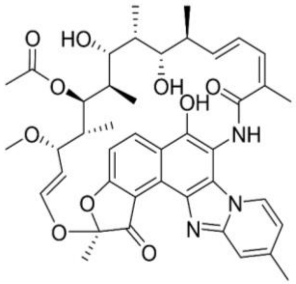	Molecular formula: $C_{43}H_{51}N_3O_{11}$<br><br>Monoisotopic mass (Da): 785.3518<br><br>pKa (strongest acidic): 6.72<br><br>pKa (strongest basic): 5.89<br><br>Partition coefficient (Log P): 4.599<br><br>Intrinsic solubility (mg mL^{-1}): -7.704 (Low)	[M+H]$^+$; [M-H]$^-$	786.3596; 784.3451	91.0542; 95.0855; 151.0754; 754.3334; 65.0386	784.3451; 360.0987; 482.1718; 156.0567; 65.9985	

Group/Class - Pharmacologically active Substance	Physicochemical properties	Monitored ion	Theoretical exact mass MS (*m/z*)	Fragment ions (*m/z*) MS^2		
				$[M+H]^+$	$[M-H]^-$	Others
**Antibiotic Growth Promoters**						
Avilamycin (Dichloroisoeverninic acid)<br><br>CAS registration number:<br>4101-80-8<br><br>ChemSpider ID:<br>10311534<br><br>PubChem CID:<br>21724963<br><br>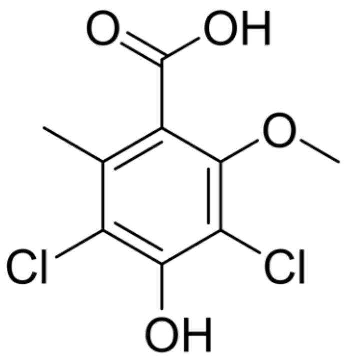	Molecular formula:<br>$C_9H_8Cl_2O_4$<br><br>Monoisotopic mass (Da): 249.9794<br><br>pKa (strongest acidic): 3.4<br><br>pKa (strongest basic): -<br><br>Partition coefficient (Log P): 2.891<br><br>Intrinsic solubility (mg mL^{-1}): -2.95 (High)	$[M+H]^-$	248.9727		204.9818;<br>189.9583;<br>174.9712;<br>169.0051;<br>233.9481	
Halquinol/Chlorhydroxyquinoline (7-Chloro-8-quinolinol)<br><br>CAS registration number:<br>876-86-8	Molecular formula:<br>C_9H_6ClNO<br><br>Monoisotopic mass (Da): 179.0132	$[M+H]^+$	180.0211	145.0522;<br>162.0105;<br>117.0573;<br>127.0417;<br>116.0495		

**Group/Class - Pharmacologically active Substance**	**Physicochemical properties**	**Monitored ion**	**Theoretical exact mass MS (*m/z*)**	**Fragment ions (*m/z*) MS^2**		
				$[M+H]^+$	$[M-H]^-$	Others
ChemSpider ID:<br>23089<br><br>PubChem CID:<br>24691<br><br>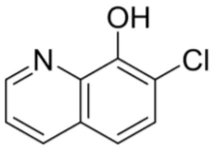	pKa (strongest acidic):<br>8.33<br><br>pKa (strongest basic):<br>4.16<br><br>Partition coefficient (Log P): 2.431<br><br>Intrinsic solubility (mg mL^{-1}): -2.367 (High)					
Halquinol/Chlorhydroxyquinoline (5,7-Dichloro-8-quinolinol/Chloroxine)<br><br>CAS registration number:<br>773-76-2	Molecular formula:<br>$C_9H_5Cl_2NO$<br><br>Monoisotopic mass (Da): 212.9743	$[M+H]^+$	213.9821	179.0132;<br>164.9976;<br>150.0105;<br>122.9996;<br>151.0183		

**Group/Class - Pharmacologically active Substance**	**Physicochemical properties**	**Monitored ion**	**Theoretical exact mass MS (*m/z*)**	**Fragment ions (*m/z*) MS^2**		
				[M+H]$^+$	[M-H]$^-$	Others
ChemSpider ID:<br>2621<br><br>PubChem CID:<br>2722<br><br>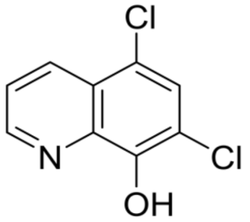 	pKa (strongest acidic):<br>7.85<br><br>pKa (strongest basic):<br>3.4<br><br>Partition coefficient (Log P): 3.035<br><br>Intrinsic solubility (mg mL^{-1}): -3.139 (High)					
Halquinol/Chlorhydroxyquinoline (5-Chloro-8-hydroxyquinoline)<br><br>CAS registration number:<br>130-16-5<br><br>ChemSpider ID:	Molecular formula:<br>C_9H_6ClNO<br><br>Monoisotopic mass (Da): 179.0132	[M+H]$^+$	180.0211	145.0522;<br>117.0573;<br>162.0105;<br>116.0495;<br>127.0417		

Group/Class - Pharmacologically active Substance	Physicochemical properties	Monitored ion	Theoretical exact mass MS (*m/z*)	Fragment ions (*m/z*) MS^2		
				$[M+H]^+$	$[M-H]^-$	Others
2715<br><br>PubChem CID: 2817<br><br>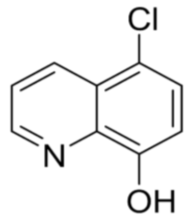	pKa (strongest acidic): 8.77<br><br>pKa (strongest basic): 4.01<br><br>Partition coefficient (Log P): 2.431<br><br>Intrinsic solubility (mg mL^{-1}): -2.367 (High)					
Virginiamycin (Virginiamycin M_1)<br><br>CAS registration number: 21411-53-0<br><br>ChemSpider ID: 10222381	Molecular formula: $C_{28}H_{35}N_3O_7$<br><br>Monoisotopic mass (Da): 525.2470	$[M+H]^+$;<br>$[M-H]^-$;<br>$[M+Na]^+$	526.2548;<br>524.2402;<br>548.2367	109.1016;<br>355.1281;<br>508.2439;<br>337.1185	245.0557;<br>219.0764;<br>420.2180;<br>263.0662;<br>206.1185	$[M+Na]^+$<br>287.0638;<br>243.0737;<br>109.1012;<br>284.1618;<br>114.0550

Group/Class - Pharmacologically active Substance	Physicochemical properties	Monitored ion	Theoretical exact mass MS (*m/z*)	Fragment ions (*m/z*) MS^2		
				[M+H]$^+$	[M-H]$^-$	Others
PubChem CID: 5459319<br>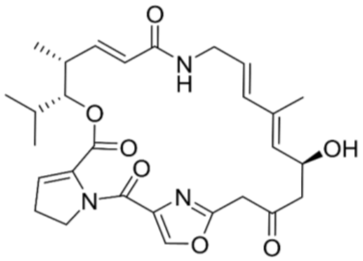	pKa (strongest acidic): 13.17<br><br>pKa (strongest basic): -0.62<br><br>Partition coefficient (Log P): 2.376<br><br>Intrinsic solubility (mg mL^{-1}): -4.097 (Moderate)					
**Anticoccidials**						
Amprolium<br><br>CAS registration number: 121-25-5	Molecular formula: $C_{14}H_{19}N_4$<br><br>Monoisotopic mass (Da): 243.1604	[M]$^+$;<br>[M+Na]$^+$;<br>[M+NH$_4$]$^+$	243.1604;<br>266.1502;<br>261.1948			[M]$^+$<br>150.1026;<br>122.0713;<br>94.0651;<br>174.1026;

**Group/Class - Pharmacologically active Substance**	**Physicochemical properties**	**Monitored ion**	**Theoretical exact mass MS (*m/z*)**	**Fragment ions (*m/z*) MS^2**		
				$[M+H]^+$	$[M-H]^-$	Others
ChemSpider ID:<br>66070<br><br>PubChem CID:<br>73341<br><br>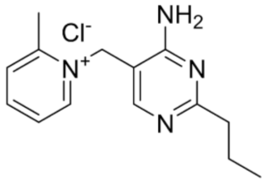	pKa (strongest acidic): -<br><br>pKa (strongest basic):<br>5.33<br><br>Partition coefficient (Log P): -2.308<br><br>Intrinsic solubility ($mg\ mL^{-1}$): The molecule cannot be neutralized and the calculation is not defined for molecules with non-zero charge					108.0556<br><br>$[M+Na]^+$<br>150.1026;<br>122.0713;<br>94.0651;<br>174.1026;<br>108.0556<br><br>$[M+NH_4]^+$<br>150.1026;<br>122.0713;<br>94.0651;<br>174.1026;<br>108.0556
Clopidol<br><br>CAS registration number:<br>2971-90-6<br><br>ChemSpider ID:	Molecular formula:<br>$C_7H_7Cl_2NO$<br><br>Monoisotopic mass (Da): 190.9899	$[M+H]^+$;<br>$[M+Na]^+$	191.9978;<br>213.9797	101.0153;<br>86.9996;<br>65.0386;<br>72.9840;<br>128.0262		$[M+Na]^+$<br>84.9606;<br>170.9613;<br>120.9815;<br>101.0153;<br>86.9996

Group/Class - Pharmacologically active Substance	Physicochemical properties	Monitored ion	Theoretical exact mass MS (*m/z*)	Fragment ions (*m/z*) MS^2		
				$[M+H]^+$	$[M-H]^-$	Others
17084<br><br>PubChem CID: 18087<br><br>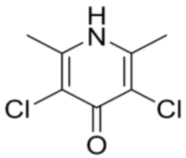	No ionizable atoms found.<br><br>pKa (strongest acidic): -<br><br>pKa (strongest basic): -<br><br>Partition coefficient (Log P): 1.144<br><br>Intrinsic solubility (mg mL^{-1}): -2.335 (High)					
Diaveridine<br><br>CAS registration number: 5355-16-8<br><br>ChemSpider ID:	Molecular formula: $C_{13}H_{16}N_4O_2$<br><br>Monoisotopic mass (Da): 260.1268	$[M+H]^+$; $[M+Na]^+$	261.1346; 283.1166	245.1033; 123.0665; 95.0491; 81.0447; 245.1033		$[M+Na]^+$ 74.0964; 152.1182

Group/Class - Pharmacologically active Substance	Physicochemical properties	Monitored ion	Theoretical exact mass MS (*m/z*)	Fragment ions (*m/z*) MS^2		
				$[M+H]^+$	$[M-H]^-$	Others
20162<br><br>PubChem CID:<br>21453<br><br>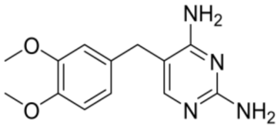	pKa (strongest acidic): -<br><br>pKa (strongest basic):<br>7.16<br><br>Partition coefficient<br>(Log P): 1.442<br><br>Intrinsic solubility<br>(mg mL^{-1}): -2.803<br>(High)					
Decoquinate<br><br>CAS registration number:<br>18507-89-6<br><br>ChemSpider ID:	Molecular formula:<br>$C_{24}H_{35}NO_5$<br><br>Monoisotopic mass<br>(Da): 417.2510	$[M+H]^+$;<br>$[M-H]^-$;<br>$[M+Na]^+$	418.2588;<br>416.2443;<br>440.2407	390.2275;<br>222.0397;<br>148.0393;<br>121.0284;<br>250.0710	118.0298;<br>275.0799;<br>65.9985;<br>218.0095;<br>246.0408	$[M+Na]^+$<br>321.1571;<br>236.0596;<br>130.1590;<br>167.9927;<br>261.1335

Group/Class - Pharmacologically active Substance	Physicochemical properties	Monitored ion	Theoretical exact mass MS (*m/z*)	Fragment ions (*m/z*) MS^2		
				[M+H]$^+$	[M-H]$^-$	Others
27081<br><br>PubChem CID:<br>29112<br><br>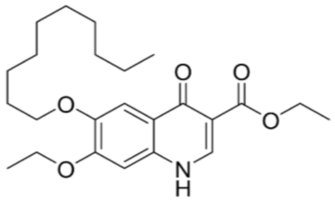	pKa (strongest acidic): 5.82<br><br>pKa (strongest basic): -<br><br>Partition coefficient (Log P): 6.164<br><br>Intrinsic solubility (mg mL^{-1}): -7.341 (Low)					
Diclazuril<br><br>CAS registration number:<br>101831-37-2<br><br>ChemSpider ID:	Molecular formula: $C_{17}H_9Cl_3N_4O_2$<br><br>Monoisotopic mass (Da): 405.9786	[M-H]$^-$	404.9718		337.9711;<br>298.9783;<br>186.9943;<br>404.9718;<br>235.0070	

Group/Class - Pharmacologically active Substance	Physicochemical properties	Monitored ion	Theoretical exact mass MS (*m/z*)	Fragment ions (*m/z*) MS^2		
				[M+H]$^+$	[M-H]$^-$	Others
401855<br><br>PubChem CID:<br>456389<br><br>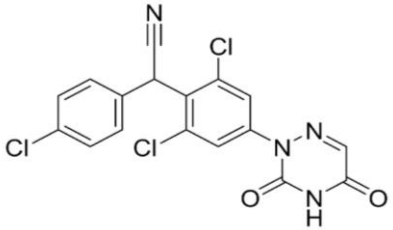	pKa (strongest acidic): 6.5<br><br>pKa (strongest basic): -<br><br>Partition coefficient (Log P): 4.234<br><br>Intrinsic solubility (mg mL^{-1}): 5.448 (Low)					
Ethopabate<br><br>CAS registration number:<br>59-06-3<br><br>ChemSpider ID:	Molecular formula: $C_{12}H_{15}NO_4$<br><br>Monoisotopic mass (Da): 237.0996	[M+H]$^+$;<br>[M-H]$^-$;<br>[M+Na]$^+$	238.1074;<br>236.0928;<br>260.0893	238.1074;<br>136.0393;<br>164.0706;<br>206.0812	208.0604;<br>206.0448;<br>192.0291;<br>207.0526	[M+Na]$^+$<br>147.1168;<br>101.0597;<br>182.0448;<br>168.0655;<br>196.0968

Group/Class - Pharmacologically active Substance	Physicochemical properties	Monitored ion	Theoretical exact mass MS (*m/z*)	Fragment ions (*m/z*) MS^2		
				[M+H]$^+$	[M-H]$^-$	Others
5812<br><br>PubChem CID:<br>6034<br><br>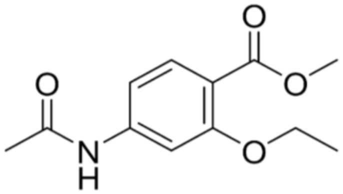	pKa (strongest acidic):<br>13.31<br><br>pKa (strongest basic): -<br><br>Partition coefficient<br>(Log P): 1.414<br><br>Intrinsic solubility<br>(mg mL^{-1}): -2.243<br>(High)					
Lasalocid (Lasalocid A)<br><br>CAS registration number:<br>25999-31-9<br><br>ChemSpider ID:	Molecular formula:<br>$C_{34}H_{54}O_8$<br><br>Monoisotopic mass<br>(Da): 590.3813	[M+H]$^+$;<br>[M-H]$^-$;<br>[M+Na]$^+$;<br>[M+NH$_4$]$^+$	591.3892;<br>589.3746;<br>613.3711;<br>608.4157	237.1849;<br>337.2737;<br>95.0855;<br>133.1012;<br>91.0542	235.0976;<br>173.0972;<br>121.0659;<br>157.0659;<br>191.1078	[M+Na]$^+$<br>377.2662;<br>559.3394;<br>557.3500;<br>359.2557;<br>481.2561

Group/Class - Pharmacologically active Substance	Physicochemical properties	Monitored ion	Theoretical exact mass MS (*m/z*)	Fragment ions (*m/z*) MS^2		
				[M+H]$^+$	[M-H]$^-$	Others
4514598<br><br>PubChem CID:<br>5360807<br>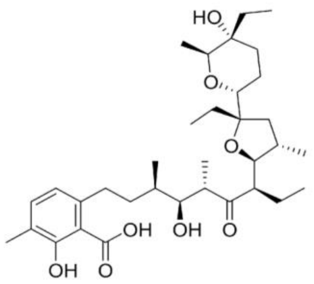	pKa (strongest acidic): 2.64<br><br>pKa (strongest basic): -<br><br>Partition coefficient (Log P): 7.665<br><br>Intrinsic solubility (mg mL^{-1}): -5.983 (Low)					[M+NH$_4$]$^+$<br>237.1849;<br>573.3786;<br>91.0542;<br>133.1012;<br>95.0855
Maduramicin<br><br>CAS registration number:<br>79356-08-4<br><br>ChemSpider ID:	Molecular formula: $C_{47}H_{80}O_{17}$<br><br>Monoisotopic mass (Da): 916.5390	[M+H]$^+$;<br>[M-H]$^-$;<br>[M+Na]$^+$;<br>[M+NH$_4$]$^+$	917.5468;<br>915.5323;<br>939.5288;<br>934.5734	877.5284;<br>633.4337;<br>451.2666;<br>473.2878;<br>433.2562	112.0530;<br>84.0217;<br>613.3593;<br>99.0452;<br>839.5162	[M+Na]$^+$<br>877.5284;<br>451.2666;<br>473.2878;<br>433.2562;<br>397.1833

**Group/Class - Pharmacologically active Substance**	**Physicochemical properties**	**Monitored ion**	**Theoretical exact mass MS (*m/z*)**	**Fragment ions (*m/z*) MS^2**		
				$[M+H]^+$	$[M-H]^-$	Others
169976<br><br>PubChem CID:<br>196129<br><br>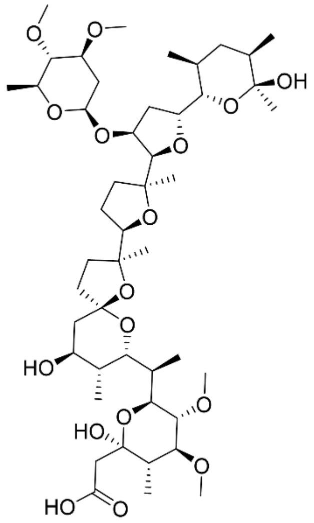	pKa (strongest acidic):<br>4.01<br><br>pKa (strongest basic): -<br><br>Partition coefficient<br>(Log P): 4.774<br><br>Intrinsic solubility<br>(mg mL^{-1}): -3.941<br>(High)					$[M+NH_4]^+$<br>629.4047;<br>375.2530;<br>109.1012;<br>67.0542;<br>91.0542
Monensin (Monensin A)<br><br>CAS registration number:<br>17090-79-8<br><br>ChemSpider ID:	Molecular formula:<br>$C_{36}H_{62}O_{11}$<br><br>Monoisotopic mass<br>(Da): 670.4287	$[M+H]^+$;<br>$[M-H]^-$;<br>$[M+Na]^+$;<br>$[M+NH_4]^+$	671.4365;<br>669.4219;<br>693.4184;<br>688.4630	461.2874;<br>479.2979;<br>443.2768;<br>501.3187;<br>461.2000	87.0452;<br>101.0608;<br>137.0972;<br>71.0502;<br>85.0295	$[M+Na]^+$<br>675.4079;<br>461.2874;<br>581.3806;<br>479.2979;<br>443.2768

Group/Class - Pharmacologically active Substance	Physicochemical properties	Monitored ion	Theoretical exact mass MS (*m/z*)	Fragment ions (*m/z*) MS^2		
				[M+H]$^+$	[M-H]$^-$	Others
389937<br><br>PubChem CID:<br>441145<br><br>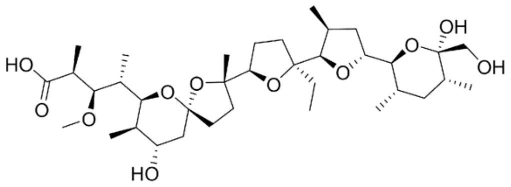	pKa (strongest acidic): 4.24<br><br>pKa (strongest basic): -<br><br>Partition coefficient (Log P): 4.816<br><br>Intrinsic solubility (mg mL^{-1}): -5.073 (Low)					$[M+NH_4]^+$<br>635.4154;<br>461.3264;<br>125.0961;<br>149.0961;<br>91.0542
Narasin (Narasin A)<br><br>CAS registration number:<br>55134-13-9<br><br>ChemSpider ID:	Molecular formula: $C_{43}H_{72}O_{11}$<br><br>Monoisotopic mass (Da): 764.5069	[M+H]$^+$;<br>[M-H]$^-$;<br>[M+Na]$^+$;<br>$[M+NH_4]^+$	765.5147;<br>763.5002;<br>787.4967;<br>782.5413	431.2404;<br>403.2455;<br>207.1356;<br>531.3316;<br>279.1567	255.1602;<br>87.0452;<br>69.0346;<br>83.0502;<br>99.0815	[M+Na]$^+$<br>431.2424;<br>403.2455;<br>207.1356;<br>531.3316;<br>279.1567

Group/Class - Pharmacologically active Substance	Physicochemical properties	Monitored ion	Theoretical exact mass MS (*m/z*)	Fragment ions (*m/z*) MS²		
				[M+H]$^+$	[M-H]$^-$	Others
58911<br><br>PubChem CID:<br>65452<br>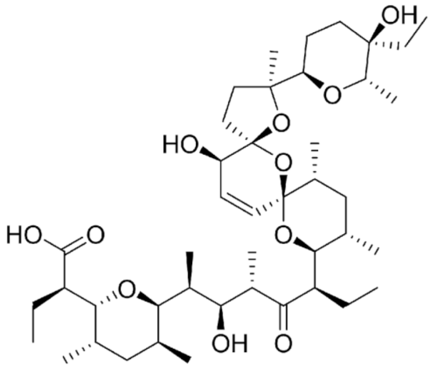	pKa (strongest acidic): 4.5<br><br>pKa (strongest basic): -<br><br>Partition coefficient (Log P): 7.877<br><br>Intrinsic solubility (mg mL^{-1}): -7.343 (Low)					[M+NH$_4$]$^+$<br>373.2373;<br>91.0542;<br>95.0855;<br>165.1274;<br>81.0699
Nicarbazin (4,4'-Dinitrocarbanilide - DNC)<br><br>CAS registration number:<br>587-90-6<br><br>ChemSpider ID:	Molecular formula: $C_{13}H_{10}N_4O_5$<br><br>Monoisotopic mass (Da): 302.0646	[M+H]$^+$;<br>[M-H]$^-$;<br>[M+Na]$^+$	303.0724;<br>301.0578;<br>325.0543	93.0573;<br>139.0502;<br>66.0464;<br>65.0386;<br>74.0151	137.0357;<br>107.0376;<br>91.0427;<br>65.9985;<br>90.0348	[M+Na]$^+$<br>248.9781;<br>245.0067;<br>125.0709;<br>169.0346;<br>219.0012

Group/Class - Pharmacologically active Substance	Physicochemical properties	Monitored ion	Theoretical exact mass MS (*m/z*)	Fragment ions (*m/z*) MS^2		
				$[M+H]^+$	$[M-H]^-$	Others
9137<br><br>PubChem CID:<br>9509<br><br>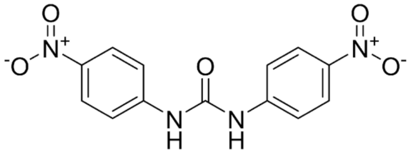	pKa (strongest acidic): 10.99<br><br>pKa (strongest basic): -<br><br>Partition coefficient (Log P): 2.999<br><br>Intrinsic solubility (mg mL^{-1}): -4.256 (Moderate)					
Robenidine<br><br>CAS registration number:<br>25875-51-8<br><br>ChemSpider ID:	Molecular formula: $C_{15}H_{13}Cl_2N_5$<br><br>Monoisotopic mass (Da): 333.0543	$[M+H]^+$;<br>$[M-H]^-$;<br>$[M+Na]^+$	334.0621;<br>332.0475;<br>356.0440	138.0105;<br>139.0058;<br>155.0371;<br>75.0229;<br>140.0262	195.0443;<br>153.0225;<br>115.0302;<br>125.0161;<br>89.0397	$[M+Na]^+$<br>107.0328;<br>133.0760;<br>125.0153;<br>218.0330

**Group/Class - Pharmacologically active Substance**	**Physicochemical properties**	**Monitored ion**	**Theoretical exact mass MS (*m/z*)**	**Fragment ions (*m/z*) MS^2**		
				$[M+H]^+$	$[M-H]^-$	Others
30671<br><br>PubChem CID:<br>33196<br><br>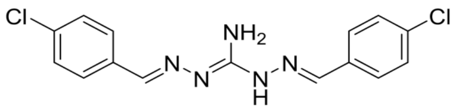	pKa (strongest acidic): -<br><br>pKa (strongest basic):<br>2.01<br><br>Partition coefficient<br>(Log P): 3.985<br><br>Intrinsic solubility<br>(mg mL^{-1}): -5.533 (Low)					
Salinomycin<br><br>CAS registration number:<br>53003-10-4<br><br>ChemSpider ID:<br>2342058	Molecular formula:<br>$C_{42}H_{70}O_{11}$<br><br>Monoisotopic mass<br>(Da): 750.4913	$[M+H]^+$;<br>$[M-H]^-$;<br>$[M+Na]^+$;<br>$[M+NH_4]^+$	751.4991;<br>749.4845;<br>773.4810;<br>768.5256	431.2474;<br>265.1410;<br>207.1356;<br>91.0542;<br>67.0542	241.1434;<br>407.2428;<br>337.2010;<br>221.1172;<br>259.1540	$[M+Na]^+$<br>431.2474;<br>265.1410;<br>207.1356;<br>91.0542;<br>530.3155

Group/Class - Pharmacologically active Substance	Physicochemical properties	Monitored ion	Theoretical exact mass MS (*m/z*)	Fragment ions (*m/z*) MS[2]		
				$[M+H]^+$	$[M-H]^-$	Others
PubChem CID: 3085092<br>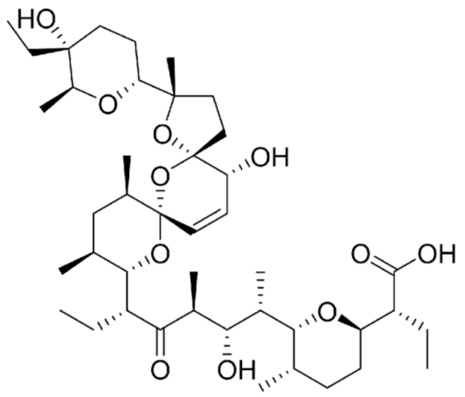	pKa (strongest acidic): 4.45<br><br>pKa (strongest basic): -<br><br>Partition coefficient (Log P): 7.512<br><br>Intrinsic solubility (mg mL^{-1}): -6.995 (Low)					$[M+NH_4]^+$ 225.1485; 91.0542; 67.0542; 81.0699; 165.1274
Toltrazuril<br><br>CAS registration number: 69004-03-1<br><br>ChemSpider ID:	Molecular formula: $C_{18}H_{14}F_3N_3O_4S$<br><br>Monoisotopic mass (Da): 425.0652	$[M+H]^+$; $[M-H]^-$	426.0730; 424.0584	192.9929; 348.0700; 325.1057; 283.0399; 148.0393	316.9813; 404.9767; 213.0919; 138.0274; 424.4000	

Group/Class - Pharmacologically active Substance	Physicochemical properties	Monitored ion	Theoretical exact mass MS (*m/z*)	Fragment ions (*m/z*) MS^2		
				[M+H]$^+$	[M-H]$^-$	Others
61859<br><br>PubChem CID:<br>68591<br><br>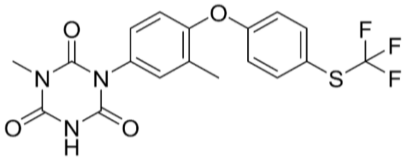	pKa (strongest acidic):<br>7.42<br><br>pKa (strongest basic): -<br><br>Partition coefficient (Log P): 5.053<br><br>Intrinsic solubility (mg mL^{-1}): -6.816 (Low)					
Trimethoprim<br><br>CAS registration number:<br>738-70-5<br><br>ChemSpider ID:	Molecular formula:<br>$C_{14}H_{18}N_4O_3$<br><br>Massa monoisotópica (Da):<br>290.1373	[M+H]$^+$	291.1452	275.1137;<br>261.0982;<br>245.1033;<br>81.0447;<br>123.0665		

Group/Class - Pharmacologically active Substance	Physicochemical properties	Monitored ion	Theoretical exact mass MS (*m/z*)	Fragment ions (*m/z*) MS^2		
				$[M+H]^+$	$[M-H]^-$	Others
5376<br><br>PubChem CID:<br>5578<br>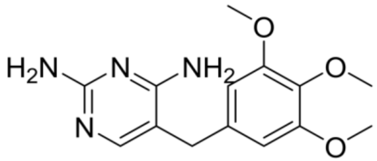	pKa (strongest acidic): -<br><br>pKa (strongest basic): 7.16<br><br>Partition coefficient (Log P): 1.284<br><br>Intrinsic solubility (mg mL^{-1}): -2.801 (High)					
**Anti-inflammatories/Steroidal**						
Prednisolone<br><br>CAS registration number:<br>50-24-8	Molecular formula: $C_{21}H_{28}O_5$<br><br>Monoisotopic mass (Da): 360.1931	$[M+H]^+$	361.2010	343.1901;<br>147.0804;<br>121.0648;<br>91.0542;<br>325.1798		

Group/Class - Pharmacologically active Substance	Physicochemical properties	Monitored ion	Theoretical exact mass MS (*m/z*)	Fragment ions (*m/z*) MS^2		
				[M+H]$^+$	[M-H]$^-$	Others
ChemSpider ID: 5552<br><br>PubChem CID: 5755<br><br>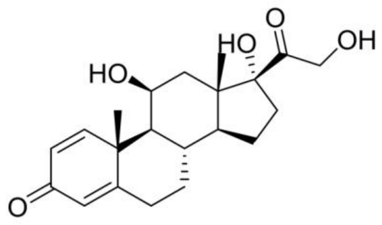	pKa (strongest acidic): 12.59<br><br>pKa (strongest basic): -<br><br>Partition coefficient (Log P): 1.273<br><br>Intrinsic solubility (mg mL^{-1}): -3.46 (High)					
Prednisone<br><br>CAS registration number: 53-03-2<br><br>ChemSpider ID: 5656	Molecular formula: $C_{21}H_{26}O_5$<br><br>Monoisotopic mass (Da): 358.1775	[M+H]$^+$; [M-H]$^-$	359.1853; 357.1708	147.0804; 91.0542; 359.1853; 341.1747; 237.1271	327.1602; 123.0452; 149.0608; 122.0373; 121.0659	

Group/Class - Pharmacologically active Substance	Physicochemical properties	Monitored ion	Theoretical exact mass MS (*m/z*)	Fragment ions (*m/z*) MS^2		
				[M+H]$^+$	[M-H]$^-$	Others
PubChem CID: 5865<br>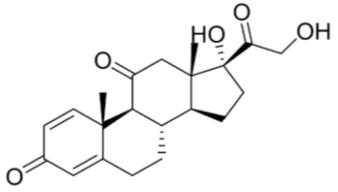	pKa (strongest acidic): 12.58<br><br>pKa (strongest basic): -<br><br>Partition coefficient (Log P): 1.659<br><br>Intrinsic solubility (mg mL^{-1}): -3.379 (High)					
**Anti-inflammatories Prospect/Steroidal**<br>*Isoflupredone acetate/9-Fluoroprednisolone acetate*<br><br>CAS registration number:	Molecular formula: $C_{23}H_{29}FO_6$<br><br>Monoisotopic mass (Da): 420.1943	[M+H]$^+$	421.2021	341.1747; 91.0542; 147.0804; 265.1587; 121.0648		

Group/Class - Pharmacologically active Substance	Physicochemical properties	Monitored ion	Theoretical exact mass MS (*m/z*)	Fragment ions (*m/z*) MS^2		
				[M+H]$^+$	[M-H]$^-$	Others
338-98-7<br><br>ChemSpider ID:<br>194836<br><br>PubChem CID:<br>224246<br><br>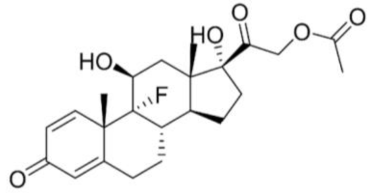	pKa (strongest acidic):<br>12.57<br><br>pKa (strongest basic): -<br><br>Partition coefficient (Log P): 1.758<br><br>Intrinsic solubility (mg mL^{-1}): -3.834 (High)					
Flumetasone<br><br>CAS registration number:<br>2135-17-3<br><br>ChemSpider ID:<br>15632	Molecular formula:<br>$C_{22}H_{28}F_2O_5$<br><br>Monoisotopic mass (Da): 410.1899<br><br>pKa (strongest acidic):	[M+H]$^+$	411.1978	121.0648;<br>253.1223;<br>91.0542;<br>391.1915;<br>95.0491		

Group/Class - Pharmacologically active Substance	Physicochemical properties	Monitored ion	Theoretical exact mass MS (*m/z*)	Fragment ions (*m/z*) MS^2		
				[M+H]$^+$	[M-H]$^-$	Others
PubChem CID: 16490<br>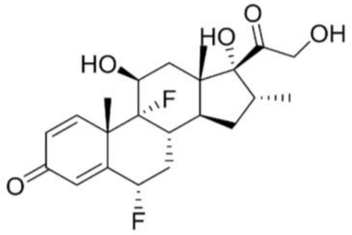	12.42<br><br>pKa (strongest basic): -<br><br>Partition coefficient (Log P): 1.342<br><br>Intrinsic solubility (mg mL^{-1}): -3.918 (Moderate)					
**Anti-Inflammatory/Non-Steroidal**						
Mefenamic acid<br><br>CAS registration number: 61-68-7<br><br>ChemSpider ID:	Molecular formula: $C_{15}H_{15}NO_2$<br><br>Monoisotopic mass (Da): 241.1097	[M+H]$^+$; [M-H]$^-$	242.1176; 240.1030	224.1070; 209.0835; 180.0808; 208.0757; 181.0886	196.1132; 192.0819; 180.0819; 194.0975; 179.0866	

Group/Class - Pharmacologically active Substance	Physicochemical properties	Monitored ion	Theoretical exact mass MS (*m/z*)	Fragment ions (*m/z*) MS^2		
				[M+H]$^+$	[M-H]$^-$	Others
3904<br><br>PubChem CID:<br>4044<br><br>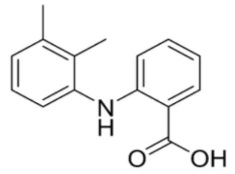	pKa (strongest acidic):<br>2.47<br><br>pKa (strongest basic):<br>5.04<br><br>Partition coefficient<br>(Log P): 4.402<br><br>Intrinsic solubility<br>(mg mL^{-1}): -4.068<br>(Moderate)					
Tolfenamic acid<br><br>CAS registration number:<br>13710-19-5<br><br>ChemSpider ID:<br>3904	Molecular formula:<br>$C_{14}H_{12}ClNO_2$<br><br>Monoisotopic mass<br>(Da): 261.0551<br><br>pKa (strongest acidic):<br>4.17	[M+H]$^+$;<br>[M-H]$^-$	262.0629;<br>260.0484	180.0804;<br>229.0281;<br>244.0517;<br>209.0837;<br>208.0763	216.0575;<br>228.0211;<br>242.0367;<br>107.0496	

Group/Class - Pharmacologically active Substance	Physicochemical properties	Monitored ion	Theoretical exact mass MS (m/z)	Fragment ions (m/z) MS^2		
				$[M+H]^+$	$[M-H]^-$	Others
PubChem CID:<br>610479<br><br>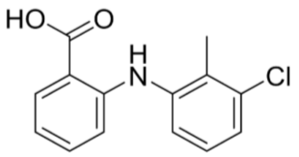	pKa (strongest basic):<br>1.8<br><br>Partition coefficient (Log P): 5.488<br><br>Intrinsic solubility (mg mL^{-1}): -4.301 (Moderate)					
Carprofen<br><br>CAS registration number:<br>53716-49-7<br><br>ChemSpider ID:<br>2483	Molecular formula:<br>$C_{15}H_{12}ClNO_2$<br><br>Monoisotopic mass (Da): 273.0551<br><br>pKa (strongest acidic):<br>4.11	$[M-H]^-$	272.0484		228.0586;<br>226.0429;<br>190.0662;<br>188.0506;<br>162.0349	

Group/Class - Pharmacologically active Substance	Physicochemical properties	Monitored ion	Theoretical exact mass MS (*m/z*)	Fragment ions (*m/z*) MS^2		
				[M+H]$^+$	[M-H]$^-$	Others
PubChem CID:<br>2581<br>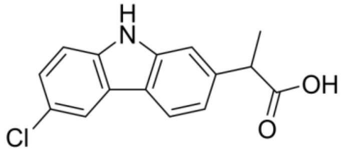	pKa (strongest basic): -<br><br>Partition coefficient (Log P): 3.876<br><br>Intrinsic solubility (mg mL^{-1}): -4.876 (Low)					
Ketoprofen<br><br>CAS registration number:<br>22071-15-4<br><br>ChemSpider ID:<br>3693	Molecular formula: $C_{16}H_{14}O_3$<br><br>Monoisotopic mass (Da): 254.0938<br><br>pKa (strongest acidic): 4	[M+H]$^+$;<br>[M-H]$^-$	255.1016;<br>253.0870	105.0335;<br>209.0961;<br>95.0491;<br>255.1016;<br>105.0447	197.0608;<br>185.0608;<br>169.0660;<br>209.0961;<br>194.0726	

Group/Class - Pharmacologically active Substance	Physicochemical properties	Monitored ion	Theoretical exact mass MS (*m/z*)	Fragment ions (*m/z*) MS^2		
				[M+H]$^+$	[M-H]$^-$	Others
PubChem CID:<br>3825<br>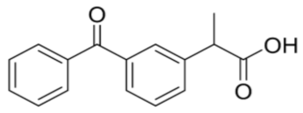	pKa (strongest basic): -<br><br>Partition coefficient (Log P): 3.613<br><br>Intrinsic solubility (mg mL^{-1}): -3.854 (Moderate)					
Diclofenac<br><br>CAS registration number:<br>15307-86-5<br><br>ChemSpider ID:<br>2925	Molecular formula:<br>$C_{14}H_{11}Cl_2NO_2$<br><br>Monoisotopic mass (Da): 295.0161<br><br>pKa (strongest acidic):<br>4.01	[M+H]$^+$;<br>[M-H]$^-$	296.0240;<br>294.0094	214.0418;<br>215.0496;<br>250.0185;<br>169.0648;<br>278.0134	250.0185;<br>214.0418;<br>178.0651	

Group/Class - Pharmacologically active Substance	Physicochemical properties	Monitored ion	Theoretical exact mass MS (*m/z*)	Fragment ions (*m/z*) MS^2		
				$[M+H]^+$	$[M-H]^-$	Others
PubChem CID:<br>3033<br>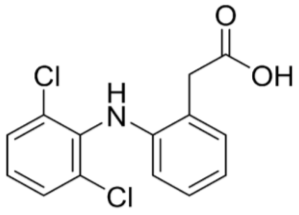	pKa (strongest basic):<br>-1.08<br><br>Partition coefficient (Log P): 4.259<br><br>Intrinsic solubility ($mg\ mL^{-1}$): -4.298 (Moderate)					
Flunixin<br><br>CAS registration number:<br>38677-85-9<br><br>ChemSpider ID:<br>34911	Molecular formula:<br>$C_{14}H_{11}F_3N_2O_2$<br><br>Monoisotopic mass (Da): 296.0767<br><br>pKa (strongest acidic):<br>0.89	$[M+H]^+$;<br>$[M-H]^-$	297.0845;<br>295.0700	279.0737;<br>277.0782;<br>264.0505;<br>259.0675;<br>236.0556	251.0802;<br>209.0520;<br>189.0458;<br>138.0349;<br>74.0037	

Group/Class - Pharmacologically active Substance	Physicochemical properties	Monitored ion	Theoretical exact mass MS (*m/z*)	Fragment ions (*m/z*) MS^2		
				[M+H]$^+$	[M-H]$^-$	Others
PubChem CID:<br>38081<br>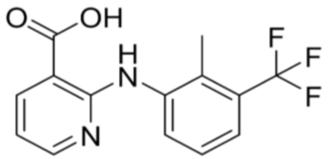	pKa (strongest basic):<br>5.37<br><br>Partition coefficient (Log P): 3.458<br><br>Intrinsic solubility (mg mL^{-1}): -3.995 (Moderate)					
Indomethacin<br><br>CAS registration number:<br>53-86-1<br><br>ChemSpider ID:<br>3584	Molecular formula:<br>$C_{19}H_{16}ClNO_4$<br><br>Monoisotopic mass (Da): 357.0762<br><br>pKa (strongest acidic):<br>4.38	[M+H]$^+$;<br>[M-H]$^-$	358.0841;<br>356.0695	138.9945;<br>139.0053;<br>75.0229;<br>74.0151;<br>174.0913	312.0797;<br>297.0562;<br>282.0328;<br>158.0611;<br>252.0222	

Group/Class - Pharmacologically active Substance	Physicochemical properties	Monitored ion	Theoretical exact mass MS (m/z)	Fragment ions (m/z) MS^2		
				$[M+H]^+$	$[M-H]^-$	Others
PubChem CID:<br>3715<br>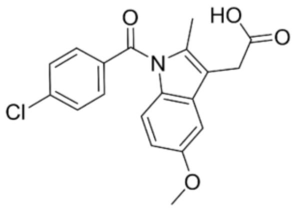	pKa (strongest basic): -<br><br>Partition coefficient (Log P): 3.53<br><br>Intrinsic solubility ($mg\ mL^{-1}$): -4.252 (Moderate)					
Meloxicam<br><br>CAS registration number:<br>71125-38-7<br><br>ChemSpider ID:<br>10442740	Molecular formula: $C_{14}H_{13}N_3O_4S_2$<br><br>Monoisotopic mass (Da): 351.0342<br><br>pKa (strongest acidic): 4.87	$[M+H]^+$;<br>$[M-H]^-$	352.0420;<br>350.0275	115.0324;<br>68.9793;<br>73.0106;<br>141.0117;<br>88.0215	146.0610;<br>286.0656;<br>113.0179;<br>119.0502;<br>97.9944	

Group/Class - Pharmacologically active Substance	Physicochemical properties	Monitored ion	Theoretical exact mass MS (*m/z*)	Fragment ions (*m/z*) MS^2		
				$[M+H]^+$	$[M-H]^-$	Others
PubChem CID: 54677470<br>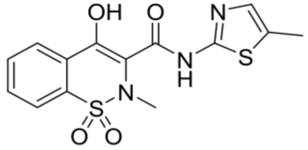	pKa (strongest basic): 0.47<br><br>Partition coefficient (Log P): 1.329<br><br>Intrinsic solubility (mg mL^{-1}): -3.947 (Moderate)					
Naproxen<br><br>CAS registration number: 22204-53-1<br><br>ChemSpider ID: 137720	Molecular formula: $C_{14}H_{14}O_3$<br><br>Monoisotopic mass (Da): 230.0938<br><br>pKa (strongest acidic): 4.24	$[M+H]^+$; $[M-H]^-$	231.1016; 229.0870	185.0961; 170.0726; 141.0699; 154.0777; 153.0699	169.0659; 170.0737; 139.0553; 141.0710; 115.0553	

Group/Class - Pharmacologically active Substance	Physicochemical properties	Monitored ion	Theoretical exact mass MS (*m/z*)	Fragment ions (*m/z*) MS^2		
				[M+H]$^+$	[M-H]$^-$	Others
PubChem CID: 156391<br>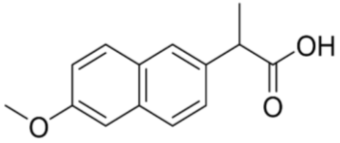	pKa (strongest basic): -<br><br>Partition coefficient (Log P): 2.986<br><br>Intrinsic solubility (mg mL^{-1}): -3.447 (High)					
Nimesulide<br><br>CAS registration number: 51803-78-2<br><br>ChemSpider ID: 4339	Molecular formula: $C_{13}H_{12}N_2O_5S$<br><br>Monoisotopic mass (Da): 308.0461<br><br>pKa (strongest acidic): 6.56	[M-H]$^-$	307.0394		307.0394; 229.0619; 198.0561; 122.0248; 78.9859	

Group/Class - Pharmacologically active Substance	Physicochemical properties	Monitored ion	Theoretical exact mass MS (*m/z*)	Fragment ions (*m/z*) MS^2		
				[M+H]$^+$	[M-H]$^-$	Others
PubChem CID: 4495<br>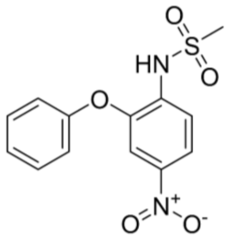	pKa (strongest basic): -<br><br>Partition coefficient (Log P): 1.787<br><br>Intrinsic solubility (mg mL^{-1}): -3.654 (High)					
Piroxycam<br><br>CAS registration number: 36322-90-4<br><br>ChemSpider ID: 10442653	Molecular formula: $C_{15}H_{13}N_3O_4S$<br><br>Monoisotopic mass (Da): 331.0621<br><br>pKa (strongest acidic): 5.06	[M+H]$^+$	332.0700	96.0444; 95.0604; 136.0867; 121.0396; 78.0338		

Group/Class - Pharmacologically active Substance	Physicochemical properties	Monitored ion	Theoretical exact mass MS (*m/z*)	Fragment ions (*m/z*) MS^2		
				$[M+H]^+$	$[M-H]^-$	Others
PubChem CID:<br>54676228<br>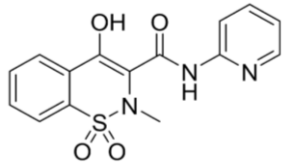	pKa (strongest basic):<br>3.89<br><br>Partition coefficient (Log P): 0.39<br><br>Intrinsic solubility (mg mL^{-1}): -3.269 (High)					
Propyphenazone<br><br>CAS registration number:<br>479-92-5<br><br>ChemSpider ID:<br>3646<br><br>PubChem CID:	Molecular formula:<br>$C_{14}H_{18}N_2O$<br><br>Monoisotopic mass (Da): 230.1414<br><br>pKa (strongest acidic): -	$[M+H]^+$	231.1492	146.0964;<br>189.1022;<br>112.1121;<br>95.0491;<br>82.0651		

Group/Class - Pharmacologically active Substance	Physicochemical properties	Monitored ion	Theoretical exact mass MS (*m/z*)	Fragment ions (*m/z*) MS^2		
				[M+H]$^+$	[M-H]$^-$	Others
3778 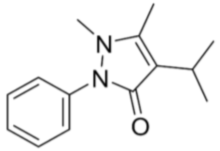	pKa (strongest basic): 0.67<br><br>Partition coefficient (Log P): 2.074<br><br>Intrinsic solubility (mg mL^{-1}): -2.757 (High)					
**Antiparasitic**<br>*Avermectin B_{1a}/Abamectin B_{1a}*<br><br>CAS registration number: 65195-55-3<br><br>ChemSpider ID:	Molecular formula: $C_{48}H_{72}O_{14}$<br><br>Monoisotopic mass (Da): 872.4917	[M+H]$^+$;<br>[M-H]$^-$;<br>[M+Na]$^+$;<br>[M+NH$_4$]$^+$	873.4995;<br>871.4849;<br>895.4814;<br>890.5260	69.0335;<br>145.0859;<br>113.0597;<br>305.2111;<br>193.1587	229.1081;<br>85.0295;<br>84.0217;<br>67.0189;<br>835.4638	[M+Na]$^+$<br>69.0335;<br>145.0859;<br>113.0597;<br>305.2111;<br>193.1587

Group/Class - Pharmacologically active Substance	Physicochemical properties	Monitored ion	Theoretical exact mass MS (*m/z*)	Fragment ions (*m/z*) MS^2		
				[M+H]$^+$	[M-H]$^-$	Others
10286553<br><br>PubChem CID: 66434889<br><br>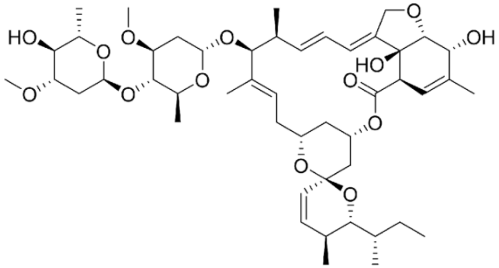	pKa (strongest acidic): 12.47<br><br>pKa (strongest basic): -<br><br>Partition coefficient (Log P): 5.848<br><br>Intrinsic solubility (mg mL^{-1}): -4.844 (Moderate)					[M+NH$_4$]$^+$ 305.2111; 113.0597; 69.0335; 95.0491; 145.0859
Doramectin<br><br>CAS registration number: 117704-25-3<br><br>ChemSpider ID: 8008478	Molecular formula: $C_{50}H_{74}O_{14}$<br><br>Monoisotopic mass (Da): 898.5073<br><br>pKa (strongest acidic): 12.47	[M+H]$^+$; [M+Na]$^+$; [M+NH$_4$]$^+$	899.5151; 921.4971; 916.5417	331.2268; 593.3473; 67.0542		

Group/Class - Pharmacologically active Substance	Physicochemical properties	Monitored ion	Theoretical exact mass MS (*m/z*)	Fragment ions (*m/z*) MS^2		
				[M+H]$^+$	[M-H]$^-$	Others
PubChem CID:<br>9832750<br>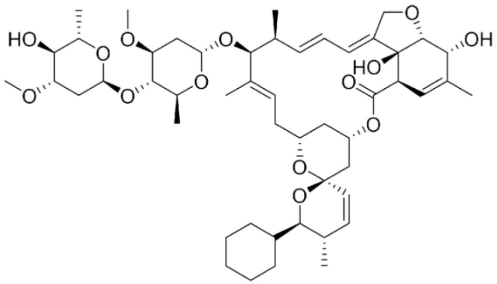	pKa (strongest basic): -<br><br>Partition coefficient (Log P): 6.274<br><br>Intrinsic solubility (mg mL^{-1}): -5.467 (Low)					
Emamectin (Emamectin B_{1a})<br><br>CAS registration number:<br>119791-41-2<br><br>ChemSpider ID:<br>9311801	Molecular formula:<br>$C_{49}H_{75}NO_{13}$<br><br>Monoisotopic mass (Da): 885.5233<br><br>pKa (strongest acidic): 12.47	[M+H]$^+$;<br>[M-H]$^-$	886.5311;<br>884.5166	158.1176;<br>82.0651;<br>67.0417;<br>126.0913;<br>91.0542	242.1398;<br>84.0217;<br>97.0659;<br>848.4954;<br>840.5267	

Group/Class - Pharmacologically active Substance	Physicochemical properties	Monitored ion	Theoretical exact mass MS (*m/z*)	Fragment ions (*m/z*) MS^2		
				$[M+H]^+$	$[M-H]^-$	Others
PubChem CID:<br>11549937<br>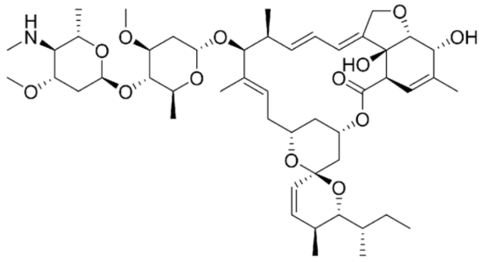	pKa (strongest basic): -<br><br>Partition coefficient (Log P): 6.724<br><br>Intrinsic solubility ($mg\ mL^{-1}$): -0.079 (High)					
Eprinomectin (Eprinomectin B_{1a})<br><br>CAS registration number:<br>133305-88-1<br><br>ChemSpider ID:<br>16736607<br><br>PubChem CID:	Molecular formula:<br>$C_{50}H_{75}NO_{14}$<br><br>Monoisotopic mass (Da): 913.5182<br><br>pKa (strongest acidic):<br>12.49	$[M+H]^+$;<br>$[M+Na]^+$	914.5260;<br>936.5080	186.1125;<br>112.0757;<br>154.0863;<br>68.0495;<br>67.0542		$[M+Na]^+$<br>186.1125;<br>112.0757;<br>154.0863;<br>68.0495;<br>67.0542

Group/Class - Pharmacologically active Substance	Physicochemical properties	Monitored ion	Theoretical exact mass MS (*m/z*)	Fragment ions (*m/z*) MS^2		
				$[M+H]^+$	$[M-H]^-$	Others
6444397<br>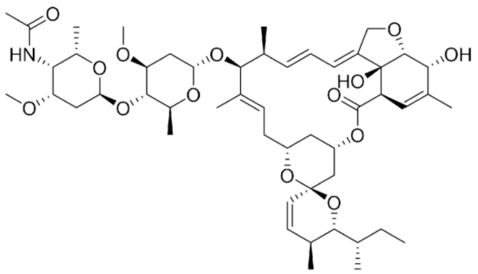	pKa (strongest basic):<br>-1.34<br><br>Partition coefficient<br>(Log P): 5.56<br><br>Intrinsic solubility<br>(mg mL^{-1}):<br>-4.915 (Moderate)					
Ivermectin B_{1a}/22,23-Dihydroavermectin B_1<br><br>CAS registration number:<br>70288-86-7<br><br>ChemSpider ID:<br>7988461	Molecular formula:<br>$C_{48}H_{74}O_{14}$<br><br>Monoisotopic mass<br>(Da): 874.5073<br><br>pKa (strongest acidic):<br>12.47	$[M+H]^+$;<br>$[M+Na]^+$;<br>$[M+NH_4]^+$	875.5151;<br>897.4971;<br>892.5417	569.3473;<br>307.2268;<br>714.4337;<br>570.3551		$[M+Na]^+$<br>713.4259;<br>569.3473;<br>307.2268;<br>714.4337;<br>570.3551<br><br>$[M+NH_4]^+$

Group/Class - Pharmacologically active Substance	Physicochemical properties	Monitored ion	Theoretical exact mass MS (*m/z*)	Fragment ions (*m/z*) MS^2		
				[M+H]$^+$	[M-H]$^-$	Others
PubChem CID: 6321424<br>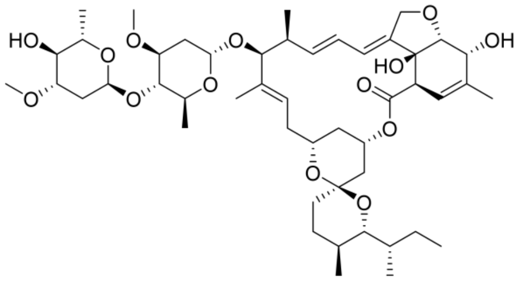	pKa (strongest basic): -<br><br>Partition coefficient (Log P): 5.826<br><br>Intrinsic solubility (mg mL^{-1}): 1.957 (High)					569.3473; 307.2268; 95.0491; 113.0597; 69.0335
Albendazole<br><br>CAS registration number: 54965-21-8<br><br>ChemSpider ID: 1998<br><br>PubChem CID: 2082	Molecular formula: $C_{12}H_{15}N_3O_2S$<br><br>Monoisotopic mass (Da): 265.0880<br><br>pKa (strongest acidic): 9.68	[M+H]$^+$; [M-H]$^-$	266.0958; 264.0812	234.0692; 192.0224; 159.0427; 191.0148; 131.0478	189.0002; 232.0550; 159.9975; 161.0053; 81.9757	

**Group/Class - Pharmacologically active Substance**	**Physicochemical properties**	**Monitored ion**	**Theoretical exact mass MS (*m/z*)**	**Fragment ions (*m/z*) MS^2**		
				$[M+H]^+$	$[M-H]^-$	Others
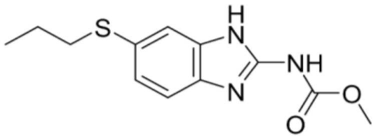	pKa (strongest basic): 4.21<br><br>Partition coefficient (Log P): 3.205<br><br>Intrinsic solubility (mg mL^{-1}): -4.382 (Moderate)					
Albendazole metabolite (Albendazole sulfone)<br><br>CAS registration number: 75184-71-3<br><br>ChemSpider ID: 48031	Molecular formula: $C_{12}H_{15}N_3O_4S$<br><br>Monoisotopic mass (Da): 297.0778<br><br>pKa (strongest acidic): 8.72	$[M+H]^+$;<br>$[M-H]^-$	298.0856;<br>296.0711	266.0591;<br>131.0478;<br>224.0122;<br>159.0424;<br>191.0325	264.0448;<br>157.0282;<br>129.0332;<br>115.0301;<br>133.0282	

**Group/Class - Pharmacologically active Substance**	**Physicochemical properties**	**Monitored ion**	**Theoretical exact mass MS (*m/z*)**	**Fragment ions (*m/z*) MS^2**		
				$[M+H]^+$	$[M-H]^-$	Others
PubChem CID:<br>53174<br>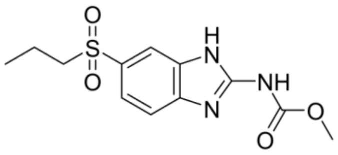	pKa (strongest basic):<br>2.74<br><br>Partition coefficient<br>(Log P): 1.673<br><br>Intrinsic solubility<br>(mg mL^{-1}): -3.437<br>(High)					
Albendazole metabolite (Albendazole sulfoxide)<br><br>CAS registration number:<br>54029-12-8<br><br>ChemSpider ID:<br>75767	Molecular formula:<br>$C_{12}H_{15}N_3O_3S$<br><br>Monoisotopic mass<br>(Da): 281.0829<br><br>pKa (strongest acidic):<br>9.26	$[M+H]^+$;<br>$[M-H]^-$	282.0907;<br>280.0761	240.0435;<br>208.0174;<br>191.0688;<br>159.0427;<br>191.0148	204.9951;<br>248.0499;<br>173.0231;<br>129.0332;<br>65.0145	

Group/Class - Pharmacologically active Substance	Physicochemical properties	Monitored ion	Theoretical exact mass MS (*m/z*)	Fragment ions (*m/z*) MS^2		
				[M+H]$^+$	[M-H]$^-$	Others
PubChem CID: 83969<br>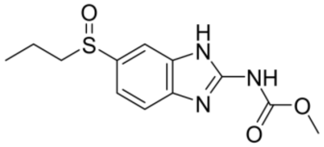	pKa (strongest basic): 3.58<br><br>Partition coefficient (Log P): 1.566<br><br>Intrinsic solubility (mg mL^{-1}): -3.389 (High)					
Febantel<br><br>CAS registration number: 58306-30-2<br><br>ChemSpider ID: 4514715<br><br>PubChem CID:	Molecular formula: $C_{20}H_{22}N_4O_6S$<br><br>Monoisotopic mass (Da): 446.1255<br><br>pKa (strongest acidic): 9.48	[M+H]$^+$; [M-H]$^-$	447.1333; 445.1187	415.1071; 383.0809; 280.0539; 116.0369; 109.0106	298.0643; 266.0393; 189.0001; 159.9975; 161.0053	

Group/Class - Pharmacologically active Substance	Physicochemical properties	Monitored ion	Theoretical exact mass MS (*m/z*)	Fragment ions (*m/z*) MS^2		
				$[M+H]^+$	$[M-H]^-$	Others
135449328<br>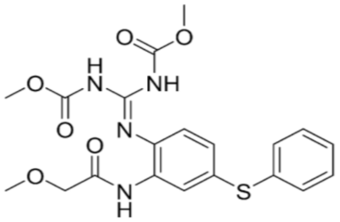	pKa (strongest basic): -<br><br>Partition coefficient (Log P): 3.166<br><br>Intrinsic solubility (mg mL^{-1}): -4.682 (Low)					
Fenbendazole<br><br>CAS registration number: 43210-67-9<br><br>ChemSpider ID: 3217<br><br>PubChem CID:	Molecular formula: $C_{15}H_{13}N_3O_2S$<br><br>Monoisotopic mass (Da): 299.0723<br><br>pKa (strongest acidic): 9.59	$[M+H]^+$;<br>$[M-H]^-$	300.0801;<br>298.0656	268.0536;<br>190.0068;<br>159.0426;<br>109.0106;<br>131.0478	266.0394;<br>189.0002;<br>159.9975;<br>161.0053;<br>81.9757	

Group/Class - Pharmacologically active Substance	Physicochemical properties	Monitored ion	Theoretical exact mass MS (*m/z*)	Fragment ions (*m/z*) MS^2		
				[M+H]$^+$	[M-H]$^-$	Others
3334<br>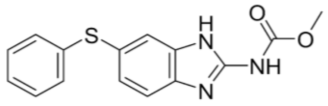	pKa (strongest basic): 4.06<br><br>Partition coefficient (Log P): 3.99<br><br>Intrinsic solubility (mg mL^{-1}): -5.36 (Low)					
Fenbendazole metabolite (Fenbendazole sulfone)<br><br>CAS registration number: 54029-20-8<br><br>ChemSpider ID: 142389<br><br>PubChem CID:	Molecular formula: $C_{15}H_{13}N_3O_4S$<br><br>Monoisotopic mass (Da): 331.0621<br><br>pKa (strongest acidic): 8.79	[M+H]$^+$; [M-H]$^-$	332.0700; 330.0554	300.0437; 159.0427; 131.0478; 104.0369; 77.0260	298.0292; 160.0152; 129.0332; 115.0301; 106.0173	

Group/Class - Pharmacologically active Substance	Physicochemical properties	Monitored ion	Theoretical exact mass MS (*m/z*)	Fragment ions (*m/z*) MS^2		
				[M+H]$^+$	[M-H]$^-$	Others
162136<br>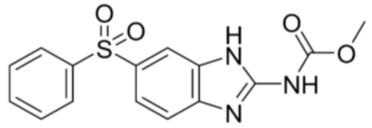	pKa (strongest basic): 2.82<br><br>Partition coefficient (Log P): 2.756<br><br>Intrinsic solubility (mg mL^{-1}): -4.397 (Moderate)					
Flubendazole<br><br>CAS registration number: 31430-15-6<br><br>ChemSpider ID: 32932<br><br>PubChem CID:	Molecular formula: $C_{16}H_{12}FN_3O_3$<br><br>Monoisotopic mass (Da): 313.0857<br><br>pKa (strongest acidic): 9.17	[M+H]$^+$	314.0936	74.0150; 282.0673; 123.0241; 123.0353; 75.0229		

Group/Class - Pharmacologically active Substance	Physicochemical properties	Monitored ion	Theoretical exact mass MS (*m/z*)	Fragment ions (*m/z*) MS^2		
				[M+H]$^+$	[M-H]$^-$	Others
35802<br>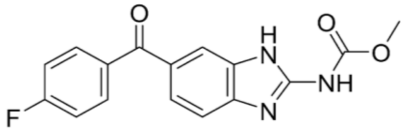	pKa (strongest basic): 3.42<br><br>Partition coefficient (Log P): 3.403<br><br>Intrinsic solubility (mg mL^{-1}): -5.207 (Low)					
Flubendazole metabolite (2-Aminoflubendazole)<br><br>CAS registration number: 82050-13-3<br><br>ChemSpider ID: 22797878	Molecular formula: $C_{14}H_{10}FN_3O$<br><br>Monoisotopic mass (Da): 255.0802<br><br>pKa (strongest acidic): 12.01	[M+H]$^+$	256.0881	256.0881; 123.0241; 113.0396; 123.0353; 75.0229		

**Group/Class - Pharmacologically active Substance**	**Physicochemical properties**	**Monitored ion**	**Theoretical exact mass MS (*m/z*)**	**Fragment ions (*m/z*) MS^2**		
				$[M+H]^+$	$[M-H]^-$	Others
PubChem CID: 57358774<br>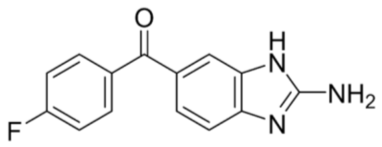	pKa (strongest basic): 6.35<br><br>Partition coefficient (Log P): 2.717<br><br>Intrinsic solubility (mg mL^{-1}): -4.857 (Low)					
Mebendazole<br><br>CAS registration number: 31431-39-7<br><br>ChemSpider ID: 3890<br><br>PubChem CID:	Molecular formula: $C_{16}H_{13}N_3O_3$<br><br>Monoisotopic mass (Da): 295.0951<br><br>pKa (strongest acidic): 9.17	$[M+H]^+$;<br>$[M-H]^-$	296.1030;<br>294.0884	264.0768;<br>130.0400;<br>104.0369;<br>95.0491;<br>105.0447	262.0622;<br>129.0332;<br>115.0302;<br>160.0152;<br>106.0173	

Group/Class - Pharmacologically active Substance	Physicochemical properties	Monitored ion	Theoretical exact mass MS (*m/z*)	Fragment ions (*m/z*) MS^2		
				[M+H]+	[M-H]-	Others
4030<br>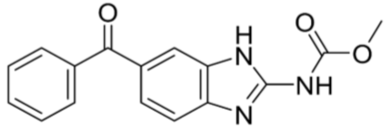	pKa (strongest basic): 3.42<br><br>Partition coefficient (Log P): 3.26<br><br>Intrinsic solubility (mg mL^{-1}): -4.937 (Low)					
Oxibendazole<br><br>CAS registration number: 20559-55-1<br><br>ChemSpider ID: 4461<br><br>PubChem CID:	Molecular formula: $C_{12}H_{15}N_3O_3$<br><br>Monoisotopic mass (Da): 249.1108<br><br>pKa (strongest acidic): 10	[M+H]+	250.1186	218.0924; 176.0455; 148.0505; 81.0335; 80.0495		

**Group/Class - Pharmacologically active Substance**	**Physicochemical properties**	**Monitored ion**	**Theoretical exact mass MS (*m/z*)**	**Fragment ions (*m/z*) MS^2**		
				[M+H]$^+$	[M-H]$^-$	Others
4622<br>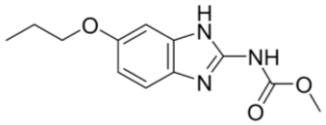	pKa (strongest basic): 4.73<br><br>Partition coefficient (Log P): 2.522<br><br>Intrinsic solubility (mg mL^{-1}): -3.412 (High)					
Oxfendazole<br><br>CAS registration number: 53716-50-0<br><br>ChemSpider ID: 37316<br><br>PubChem CID:	Molecular formula: $C_{15}H_{13}N_3O_3S$<br><br>Monoisotopic mass (Da): 315.0672<br><br>pKa (strongest acidic): 9.26	[M+H]$^+$;<br>[M-H]$^-$	316.0750;<br>314.0605	284.0488;<br>191.0689;<br>223.0576;<br>299.0723;<br>159.0427	282.0332;<br>204.9954;<br>237.0203;<br>173.0233	

Group/Class - Pharmacologically active Substance	Physicochemical properties	Monitored ion	Theoretical exact mass MS (*m/z*)	Fragment ions (*m/z*) MS^2		
				[M+H]$^+$	[M-H]$^-$	Others
40854<br>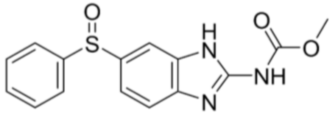	pKa (strongest basic): 3.57<br><br>Partition coefficient (Log P): 2.615<br><br>Intrinsic solubility (mg mL^{-1}): -4.359 (Moderate)					
Thiabendazole<br><br>CAS registration number: 148-79-8<br><br>ChemSpider ID: 5237<br><br>PubChem CID:	Molecular formula: $C_{10}H_7N_3S$<br><br>Monoisotopic mass (Da): 201.0355<br><br>pKa (strongest acidic): 10.28	[M+H]$^+$	202.0433	175.0324; 131.0604; 65.0386; 92.0495; 104.0495		

Group/Class - Pharmacologically active Substance	Physicochemical properties	Monitored ion	Theoretical exact mass MS (*m/z*)	Fragment ions (*m/z*) MS^2		
				[M+H]$^+$	[M-H]$^-$	Others
5430<br>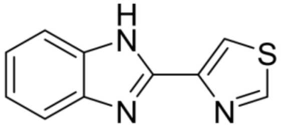	pKa (strongest basic): 4.08<br><br>Partition coefficient (Log P): 2.329<br><br>Intrinsic solubility (mg mL^{-1}): -3.097 (High)					
Triclabendazole<br><br>CAS registration number: 68786-66-3<br><br>ChemSpider ID: 45565<br><br>PubChem CID: 50248	Molecular formula: $C_{14}H_9Cl_3N_2OS$<br><br>Monoisotopic mass (Da): 357.9496<br><br>pKa (strongest acidic): 10.46	[M+H]$^+$;<br>[M-H]$^-$	358.9574;<br>356.9428	343.9339;<br>273.9962;<br>198.9727;<br>170.9779;<br>242.0241	196.9582;<br>65.9986;<br>341.9194;<br>211.9817;<br>152.9861	

Group/Class - Pharmacologically active Substance	Physicochemical properties	Monitored ion	Theoretical exact mass MS (*m/z*)	Fragment ions (*m/z*) MS^2		
				$[M+H]^+$	$[M-H]^-$	Others
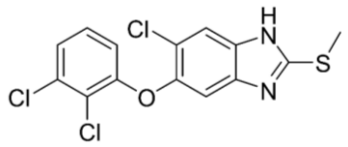	pKa (strongest basic): 4.54<br><br>Partition coefficient (Log P): 5.884<br><br>Intrinsic solubility (mg mL^{-1}): -7.325 (Low)					
Fipronil<br><br>CAS registration number: 120068-37-3<br><br>ChemSpider ID: 3235<br><br>PubChem CID:	Molecular formula: $C_{12}H_4Cl_2F_6N_4OS$<br><br>Monoisotopic mass (Da): 435.9382<br><br>pKa (strongest acidic): -	$[M-H]^-$	434.9314		329.9596; 349.9585; 183.0176; 163.0113; 143.0051	

Group/Class - Pharmacologically active Substance	Physicochemical properties	Monitored ion	Theoretical exact mass MS (*m/z*)	Fragment ions (*m/z*) MS^2		
				[M+H]$^+$	[M-H]$^-$	Others
3352<br>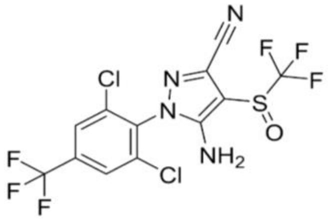	pKa (strongest basic): 0.18<br><br>Partition coefficient (Log P): 4.489<br><br>Intrinsic solubility (mg mL^{-1}): -5.959 (Low)					
Fipronil metabolite (Fipronil sulfone)<br><br>CAS registration number: 120068-36-2<br><br>ChemSpider ID: 2336427	Molecular formula: $C_{12}H_4Cl_2F_6N_4O_2S$<br><br>Monoisotopic mass (Da): 451.9331<br><br>pKa (strongest acidic): -	[M-H]$^-$	450.9263		414.9497; 281.9923; 243.9894; 218.0097; 148.0077	

Group/Class - Pharmacologically active Substance	Physicochemical properties	Monitored ion	Theoretical exact mass MS (*m/z*)	Fragment ions (*m/z*) MS^2		
				[M+H]$^+$	[M-H]$^-$	Others
PubChem CID: 3078139<br>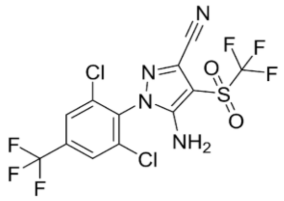	pKa (strongest basic): -0.45<br><br>Partition coefficient (Log P): 4.596<br><br>Intrinsic solubility (mg mL^{-1}): -5.967 (Low)					
Levamisole<br><br>CAS registration number: 14769-73-4<br><br>ChemSpider ID: 25037	Molecular formula: $C_{11}H_{12}N_2S$<br><br>Monoisotopic mass (Da): 204.0716<br><br>pKa (strongest acidic): -	[M+H]$^+$	205.0794	178.0685; 91.0542; 123.0263; 118.0651; 117.0699		

Group/Class - Pharmacologically active Substance	Physicochemical properties	Monitored ion	Theoretical exact mass MS (*m/z*)	Fragment ions (*m/z*) MS^2		
				$[M+H]^+$	$[M-H]^-$	Others
PubChem CID: 26879<br>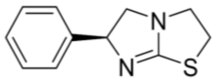	pKa (strongest basic): 11.58<br><br>Partition coefficient (Log P): 2.358<br><br>Intrinsic solubility (mg mL^{-1}): -3.223 (High)					
Moxidectin<br><br>CAS registration number: 113507-06-5<br><br>ChemSpider ID: 22901017	Molecular formula: $C_{37}H_{53}NO_8$<br><br>Monoisotopic mass (Da): 639.3766<br><br>pKa (strongest acidic): 12.55	$[M+H]^+$;<br>$[M-H]^-$;<br>$[M+Na]^+$	640.3844;<br>638.3698;<br>662.3663	91.0542;<br>528.2956;<br>98.0600;<br>199.1117;<br>498.2847	528.2956	

Group/Class - Pharmacologically active Substance	Physicochemical properties	Monitored ion	Theoretical exact mass MS (*m/z*)	Fragment ions (*m/z*) MS^2		
				[M+H]$^+$	[M-H]$^-$	Others
PubChem CID:<br>16760141<br>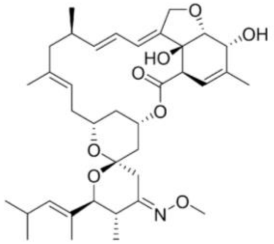	pKa (strongest basic):<br>2.01<br><br>Partition coefficient (Log P): 5.67<br><br>Intrinsic solubility (mg mL^{-1}): -6.539 (Low)					
Dimetridazole<br><br>CAS registration number:<br>551-92-8<br><br>ChemSpider ID:<br>2980	Molecular formula:<br>$C_5H_7N_3O_2$<br><br>Monoisotopic mass (Da): 141.0533<br><br>pKa (strongest acidic): -	[M+H]$^+$	142.0611	112.0631;<br>95.0604;<br>81.0447;<br>97.0396;<br>96.0682		

Group/Class - Pharmacologically active Substance	Physicochemical properties	Monitored ion	Theoretical exact mass MS (*m/z*)	Fragment ions (*m/z*) MS^2		
				[M+H]$^+$	[M-H]$^-$	Others
PubChem CID:<br>3090<br>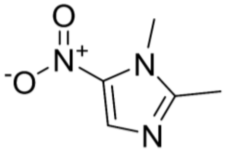	pKa (strongest basic):<br>2.9<br><br>Partition coefficient (Log P): 0.231<br><br>Intrinsic solubility (mg mL^{-1}): -0.286 (High)					
Dimetridazole metabolite (Hydroxydimetridazole/HMMNI)<br><br>CAS registration number:<br>936-05-0<br><br>ChemSpider ID:	Molecular formula:<br>$C_5H_7N_3O_3$<br><br>Monoisotopic mass (Da): 157.0482<br><br>pKa (strongest acidic):<br>13.8	[M+H]$^+$	158.0560	140.0455;<br>66.0338;<br>69.0447;<br>67.0417;<br>94.0525		

Group/Class - Pharmacologically active Substance	Physicochemical properties	Monitored ion	Theoretical exact mass MS (*m/z*)	Fragment ions (*m/z*) MS^2		
				[M+H]$^+$	[M-H]$^-$	Others
484517<br><br>PubChem CID:<br>557356<br><br>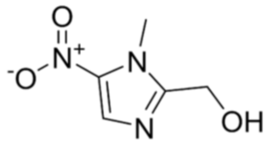	pKa (strongest basic):<br>1.36<br><br>Partition coefficient<br>(Log P): -0.586<br><br>Intrinsic solubility<br>(mg mL^{-1}): 0.103 (High)					
Ipronidazole<br><br>CAS registration number:<br>14885-29-1<br><br>ChemSpider ID:<br>25097<br><br>PubChem CID:	Molecular formula:<br>$C_7H_{11}N_3O_2$<br><br>Monoisotopic mass<br>(Da): 169.0846<br><br>pKa (strongest acidic): -	[M+H]$^+$	170.0924	109.0760;<br>84.0808;<br>124.0995;<br>96.0682;<br>125.0709		

Group/Class - Pharmacologically active Substance	Physicochemical properties	Monitored ion	Theoretical exact mass MS (*m/z*)	Fragment ions (*m/z*) MS^2		
				[M+H]$^+$	[M-H]$^-$	Others
26951<br>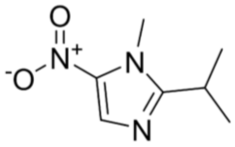	pKa (strongest basic): 2.66<br><br>Partition coefficient (Log P): 1.474<br><br>Intrinsic solubility (mg mL^{-1}): -1.173 (High)					
Ipronidazole metabolite (Hydroxy Ipronidazole)<br><br>CAS registration number: 35175-14-5<br><br>ChemSpider ID: 520172	Molecular formula: $C_7H_{11}N_3O_3$<br><br>Monoisotopic mass (Da): 185.0795<br><br>pKa (strongest acidic): 13.54	[M+H]$^+$	186.0873	168.0767; 82.0651; 186.0873; 121.0760; 138.0788		

Group/Class - Pharmacologically active Substance	Physicochemical properties	Monitored ion	Theoretical exact mass MS (*m/z*)	Fragment ions (*m/z*) MS^2		
				[M+H]$^+$	[M-H]$^-$	Others
PubChem CID: 598402<br>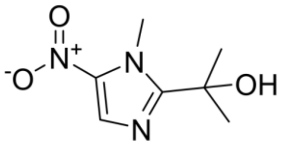	pKa (strongest basic): 1.4<br><br>Partition coefficient (Log P): 0.415<br><br>Intrinsic solubility (mg mL^{-1}): -0.751 (High)					
Metronidazole<br><br>CAS registration number: 443-48-1<br><br>ChemSpider ID: 4029	Molecular formula: $C_6H_9N_3O_3$<br><br>Monoisotopic mass (Da): 171.0638<br><br>pKa (strongest acidic): 15.42	[M+H]$^+$	172.0717	128.0455; 82.0525; 81.0447; 111.0427; 98.0475		

Group/Class - Pharmacologically active Substance	Physicochemical properties	Monitored ion	Theoretical exact mass MS (*m/z*)	Fragment ions (*m/z*) MS^2		
				$[M+H]^+$	$[M-H]^-$	Others
PubChem CID:<br>4173<br>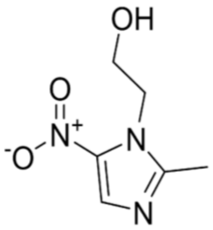	pKa (strongest basic):<br>2.81<br><br>Partition coefficient<br>(Log P): -0.459<br><br>Intrinsic solubility<br>(mg mL^{-1}): -0.16 (High)					
Metronidazole metabolite<br>*(Hydroxymetronidazole)*<br><br>CAS registration number:<br>4812-40-2<br><br>ChemSpider ID:<br>108713	Molecular formula:<br>$C_6H_9N_3O_4$<br><br>Monoisotopic mass<br>(Da): 187.0588<br><br>pKa (strongest acidic):<br>13.77	$[M+H]^+$	188.0666	144.0405;<br>126.0298;<br>123.0554;<br>68.0495;<br>80.0369		

**Group/Class - Pharmacologically active Substance**	**Physicochemical properties**	**Monitored ion**	**Theoretical exact mass MS (*m/z*)**	**Fragment ions (*m/z*) MS^2**		
				[M+H]$^+$	[M-H]$^-$	Others
PubChem CID:<br>121858<br><br>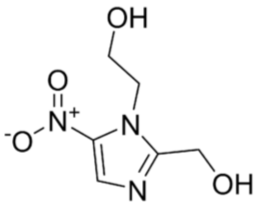	pKa (strongest basic):<br>1.27<br><br>Partition coefficient<br>(Log P): -1.277<br><br>Intrinsic solubility<br>(mg mL^{-1}): 0.237 (High)					
Ronidazole/1-Methyl-2-carbamoyloxymethyl-5-nitroimidazole<br><br>CAS registration number:<br>7681-76-7<br><br>ChemSpider ID:<br>4915	Molecular formula:<br>$C_6H_8N_4O_4$<br><br>Monoisotopic mass<br>(Da): 200.0540<br><br>pKa (strongest acidic):<br>13.97	[M+H]$^+$;<br>[M+Na]$^+$	201.0618;<br>223.0438	140.0455;<br>67.0417;<br>66.0338;<br>110.0475;<br>94.0525		[M+Na]$^+$<br>199.9941;<br>144.9883;<br>162.9988

Group/Class - Pharmacologically active Substance	Physicochemical properties	Monitored ion	Theoretical exact mass MS (*m/z*)	Fragment ions (*m/z*) MS²		
				[M+H]⁺	[M-H]⁻	Others
PubChem CID:<br>5094<br><br>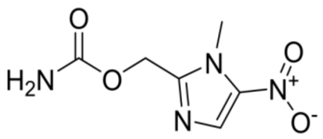	pKa (strongest basic):<br>1.34<br><br>Partition coefficient<br>(Log P): -0.479<br><br>Intrinsic solubility<br>(mg mL^{-1}): -0.46 (High)					
Praziquantel<br><br>CAS registration number:<br>55268-74-1<br><br>ChemSpider ID:<br>4722<br><br>PubChem CID:<br>4891	Molecular formula:<br>$C_{19}H_{24}N_2O_2$<br><br>Monoisotopic mass<br>(Da): 312.1832<br><br>pKa (strongest acidic): -<br><br>pKa (strongest basic):<br>-0.6	[M+H]⁺	313.1911	203.1179;<br>174.0913;<br>132.0808;<br>129.0699;<br>83.0855		

Group/Class - Pharmacologically active Substance	Physicochemical properties	Monitored ion	Theoretical exact mass MS (*m/z*)	Fragment ions (*m/z*) MS^2		
				$[M+H]^+$	$[M-H]^-$	Others
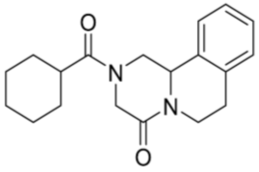	Partition coefficient (Log P): 2.302<br><br>Intrinsic solubility (mg mL^{-1}): -4.586 (Low)					
Closantel<br><br>CAS registration number: 57808-65-8<br><br>ChemSpider ID: 38827<br><br>PubChem CID: 42574	Molecular formula: $C_{22}H_{14}Cl_2I_2N_2O_2$<br><br>Monoisotopic mass (Da): 661.8516<br><br>pKa (strongest acidic): 6.41<br><br>pKa (strongest basic): -	$[M+H]^+$;<br>$[M-H]^-$	662.8595;<br>660.8449	264.0339;<br>194.0964;<br>635.8485;<br>214.0418;<br>534.8094	126.9050;<br>344.8279;<br>532.9315;<br>315.0086;<br>279.0320	

Group/Class - Pharmacologically active Substance	Physicochemical properties	Monitored ion	Theoretical exact mass MS (*m/z*)	Fragment ions (*m/z*) MS^2		
				[M+H]$^+$	[M-H]$^-$	Others
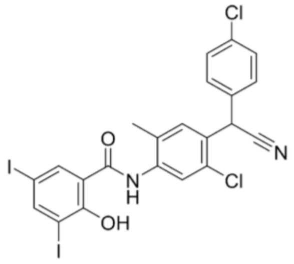	Partition coefficient (Log P): 7.713<br><br>Intrinsic solubility (mg mL^{-1}): -6.977 (Low)					
**Beta-Agonists**						
Cimaterol<br><br>CAS registration number: 54239-37-1<br><br>ChemSpider ID: 2653	Molecular formula: $C_{12}H_{17}N_3O$<br><br>Monoisotopic mass (Da): 219.1366<br><br>pKa (strongest acidic): 14.11	[M+H]$^+$	220.1444	202.1331;<br>160.0863;<br>116.0495;<br>143.0599;<br>89.0386		

Group/Class - Pharmacologically active Substance	Physicochemical properties	Monitored ion	Theoretical exact mass MS (*m/z*)	Fragment ions (*m/z*) MS^2		
				$[M+H]^+$	$[M-H]^-$	Others
PubChem CID: 2755<br>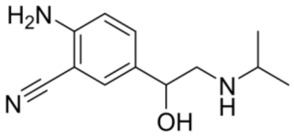	pKa (strongest basic): 9.57<br><br>Partition coefficient (Log P): 0.702<br><br>Intrinsic solubility (mg mL^{-1}): -1.605 (High)					
Clenbuterol<br><br>CAS registration number: 37148-27-9<br><br>ChemSpider ID: 2681<br><br>PubChem CID: 2783	Molecular formula: $C_{12}H_{18}Cl_2N_2O$<br><br>Monoisotopic mass (Da): 276.0791<br><br>pKa (strongest acidic): 14.06	$[M+H]^+$	277.0869	203.0135; 132.0682; 259.0763; 168.0449; 205.0118		

Group/Class - Pharmacologically active Substance	Physicochemical properties	Monitored ion	Theoretical exact mass MS (*m/z*)	Fragment ions (*m/z*) MS^2		
				$[M+H]^+$	$[M-H]^-$	Others
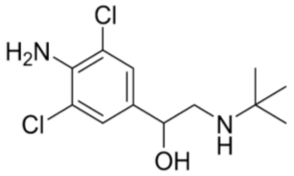	pKa (strongest basic): 9.63<br><br>Partition coefficient (Log P): 2.334<br><br>Intrinsic solubility (mg mL^{-1}): -2.992 (High)					
Ractopamine<br><br>CAS registration number: 97825-25-7<br><br>ChemSpider ID: 50604<br><br>PubChem CID:	Molecular formula: $C_{18}H_{23}NO_3$<br><br>Monoisotopic mass (Da): 301.1673<br><br>pKa (strongest acidic): 9.19	$[M+H]^+$	302.1751	107.0491; 284.1645; 164.1070; 121.0648; 136.0757		

Group/Class - Pharmacologically active Substance	Physicochemical properties	Monitored ion	Theoretical exact mass MS (*m/z*)	Fragment ions (*m/z*) MS²		
				[M+H]+	[M-H]-	Others
56052<br>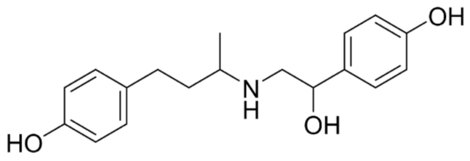	pKa (strongest basic): 9.89<br><br>Partition coefficient (Log P): 2.204<br><br>Intrinsic solubility (mg mL^{-1}): -2.46 (High)					
Salbutamol<br><br>CAS registration number: 18559-94-9<br><br>ChemSpider ID: 1999<br><br>PubChem CID: 2083	Molecular formula: $C_{13}H_{21}NO_3$<br><br>Monoisotopic mass (Da): 239.1516<br><br>pKa (strongest acidic): 10.12	$[M+H]^+$;<br>$[M-H]^-$	240.1594;<br>238.1449	222.1489;<br>166.0864;<br>148.0758;<br>121.0648;<br>91.0542	218.1187;<br>220.1343;<br>190.1237;<br>144.0455;<br>134.0373	

Group/Class - Pharmacologically active Substance	Physicochemical properties	Monitored ion	Theoretical exact mass MS (*m/z*)	Fragment ions (*m/z*) MS^2		
				[M+H]$^+$	[M-H]$^-$	Others
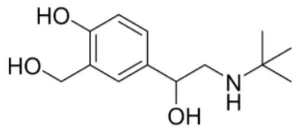	pKa (strongest basic): 9.4<br><br>Partition coefficient (Log P): 0.344<br><br>Intrinsic solubility (mg mL^{-1}): -0.796 (High)					
Zilpaterol<br><br>CAS registration number: 117827-79-9<br><br>ChemSpider ID: 2343170<br><br>PubChem CID:	Molecular formula: $C_{14}H_{19}N_3O_2$<br><br>Monoisotopic mass (Da): 261.1472<br><br>pKa (strongest acidic): 12.97	[M+H]$^+$	262.1550	244.1442; 202.0974; 185.0709; 157.0760; 130.0651		

Group/Class - Pharmacologically active Substance	Physicochemical properties	Monitored ion	Theoretical exact mass MS (*m/z*)	Fragment ions (*m/z*) MS^2		
				[M+H]$^+$	[M-H]$^-$	Others
25218684<br>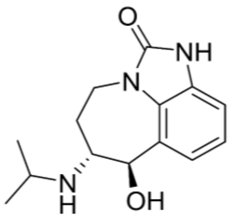	pKa (strongest basic): 9.14<br><br>Partition coefficient (Log P): 0.84<br><br>Intrinsic solubility (mg mL^{-1}): -2.459 (High)					
**Sedatives**						
Acepromazine<br><br>CAS registration number: 61-00-7<br><br>ChemSpider ID: 5852	Molecular formula: $C_{19}H_{22}N_2OS$<br><br>Monoisotopic mass (Da): 326.1447<br><br>pKa (strongest acidic): -	[M+H]$^+$	327.1526	254.0627; 239.0757; 86.0968; 178.0651; 150.0464		

Group/Class - Pharmacologically active Substance	Physicochemical properties	Monitored ion	Theoretical exact mass MS (*m/z*)	Fragment ions (*m/z*) MS^2		
				$[M+H]^+$	$[M-H]^-$	Others
PubChem CID: 6077<br>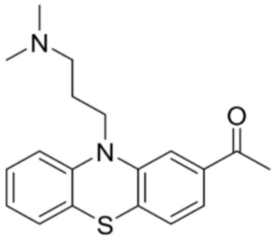	pKa (strongest basic): 9.11<br><br>Partition coefficient (Log P): 3.489<br><br>Intrinsic solubility (mg mL^{-1}): -4.437 (Moderate)					
Azaperol<br><br>CAS registration number: 2804-05-9<br><br>ChemSpider ID: 2338342<br><br>PubChem CID: 3080589	Molecular formula: $C_{19}H_{24}FN_3O$<br><br>Monoisotopic mass (Da): 329.1898<br><br>pKa (strongest acidic): 14.44	$[M+H]^+$	330.1976	312.1869; 192.1183; 149.1074; 147.0917; 121.0762		

Group/Class - Pharmacologically active Substance	Physicochemical properties	Monitored ion	Theoretical exact mass MS (*m/z*)	Fragment ions (*m/z*) MS^2		
				[M+H]$^+$	[M-H]$^-$	Others
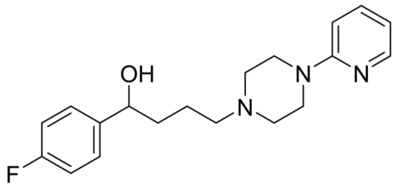	pKa (strongest basic): 8.75<br><br>Partition coefficient (Log P): 3.119<br><br>Intrinsic solubility (mg mL^{-1}): -2.692 (High)					
Azaperone<br><br>CAS registration number: 1649-18-9<br><br>ChemSpider ID: 14695<br><br>PubChem CID:	Molecular formula: $C_{19}H_{22}FN_3O$<br><br>Monoisotopic mass (Da): 327.1741<br><br>pKa (strongest acidic): -	[M+H]$^+$	328.1820	165.0710; 149.1073; 147.0917; 121.0762; 123.0241		

Group/Class - Pharmacologically active Substance	Physicochemical properties	Monitored ion	Theoretical exact mass MS (*m/z*)	Fragment ions (*m/z*) MS^2		
				[M+H]$^+$	[M-H]$^-$	Others
15443<br>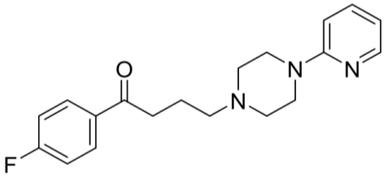	pKa (strongest basic): 8.31<br><br>Partition coefficient (Log P): 3.206<br><br>Intrinsic solubility (mg mL^{-1}): -3.149 (High)					
Carazolol<br><br>CAS registration number: 57775-29-8<br><br>ChemSpider ID: 64783<br><br>PubChem CID:	Molecular formula: $C_{18}H_{22}N_2O_2$<br><br>Monoisotopic mass (Da): 298.1676<br><br>pKa (strongest acidic): 14.03	[M+H]$^+$	299.1754	194.0964; 139.0542; 116.1070; 89.0386; 74.0600		

Group/Class - Pharmacologically active Substance	Physicochemical properties	Monitored ion	Theoretical exact mass MS (*m/z*)	Fragment ions (*m/z*) MS^2		
				[M+H]$^+$	[M-H]$^-$	Others
71739<br>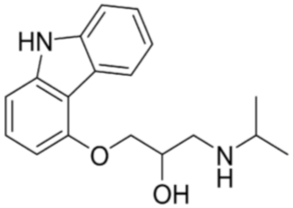	pKa (strongest basic): 9.27<br><br>Partition coefficient (Log P): 2.712<br><br>Intrinsic solubility (mg mL^{-1}): -4.379 (Moderate)					
Chlorpromazine<br><br>CAS registration number: 50-53-3<br><br>ChemSpider ID: 2625<br><br>PubChem CID:	Molecular formula: $C_{17}H_{19}ClN_2S$<br><br>Monoisotopic mass (Da): 318.0952<br><br>pKa (strongest acidic): -	[M+H]$^+$	319.1030	86.0964; 246.0139; 319.1030; 214.0418; 239.0763		

Group/Class - Pharmacologically active Substance	Physicochemical properties	Monitored ion	Theoretical exact mass MS (*m/z*)	Fragment ions (*m/z*) MS^2		
				$[M+H]^+$	$[M-H]^-$	Others
2726<br>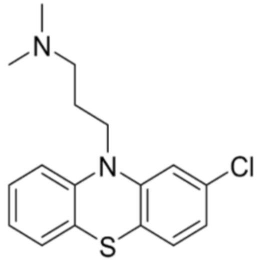	pKa (strongest basic): 9.2<br><br>Partition coefficient (Log P): 4.535<br><br>Intrinsic solubility (mg mL^{-1}): -4.848 (Low)					
**Prospecting sedatives**<br>*Xylazine*<br><br>CAS registration number: 7361-61-7<br><br>ChemSpider ID:	Molecular formula: $C_{12}H_{16}N_2S$<br><br>Monoisotopic mass (Da): 220.1029	$[M+H]^+$	221.1107	164.0528; 105.0699; 95.0491; 90.0372; 71.9902		

Group/Class - Pharmacologically active Substance	Physicochemical properties	Monitored ion	Theoretical exact mass MS (*m/z*)	Fragment ions (*m/z*) MS^2		
				$[M+H]^+$	$[M-H]^-$	Others
5505<br><br>PubChem CID: 5707<br><br>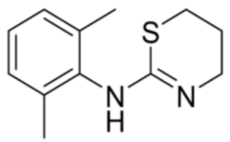	pKa (strongest acidic): -<br><br>pKa (strongest basic): 9.54<br><br>Partition coefficient (Log P): 3.633<br><br>Intrinsic solubility (mg mL^{-1}): -3.572 (Moderate)					
**Corants**						
Gentian violet/Crystal violet<br><br>CAS registration number: 548-62-9<br><br>ChemSpider ID:	Molecular formula: $C_{25}H_{30}N_3$<br><br>Monoisotopic mass (Da): 372.2434	$[M]^+$	372.2434			$[M]^+$ 356.2121; 328.1934; 239.0855; 340.1808; 268.1121

Group/Class - Pharmacologically active Substance	Physicochemical properties	Monitored ion	Theoretical exact mass MS (*m/z*)	Fragment ions (*m/z*) MS^2		
				[M+H]$^+$	[M-H]$^-$	Others
10588<br><br>PubChem CID: 11057<br><br>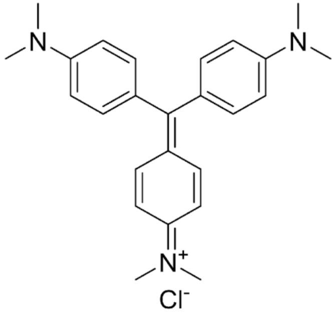	pKa (strongest acidic): -<br><br>pKa (strongest basic): 4.83<br><br>Partition coefficient (Log P): 1.396<br><br>Intrinsic solubility (mg mL^{-1}): The molecule cannot be neutralized and the calculation is not defined for molecules with non-zero charge					
Gentian violet/Crystal violet metabolite (Leucocrystal Violet)<br><br>CAS registration number: 603-48-5<br><br>ChemSpider ID:	Molecular formula: $C_{25}H_{31}N_3$<br><br>Monoisotopic mass (Da): 373.2513<br><br>pKa (strongest acidic): -	[M+H]$^+$	374.2591	358.2278; 239.1543; 237.1386; 165.0699; 152.0621		

Group/Class - Pharmacologically active Substance	Physicochemical properties	Monitored ion	Theoretical exact mass MS (*m/z*)	Fragment ions (*m/z*) MS^2		
				$[M+H]^+$	$[M-H]^-$	Others
62270<br><br>PubChem CID:<br>69048<br><br>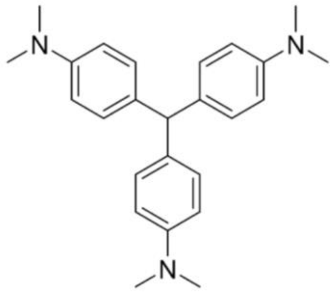	pKa (strongest basic):<br>5.36<br><br>Partition coefficient<br>(Log P): 5.81<br><br>Intrinsic solubility<br>(mg mL^{-1}): -4.153<br>(Moderate)					
Malachite green<br><br>CAS registration number:<br>569-64-2<br><br>ChemSpider ID:<br>10820	Molecular formula:<br>$C_{23}H_{25}N_2$<br><br>Monoisotopic mass<br>(Da): 329.2012<br><br>pKa (strongest acidic):	$[M]^+$	329.2012			$[M]^+$<br>313.1699;<br>208.1121;<br>165.0699;<br>239.0855;<br>163.0542

Group/Class - Pharmacologically active Substance	Physicochemical properties	Monitored ion	Theoretical exact mass MS (*m/z*)	Fragment ions (*m/z*) MS^2		
				$[M+H]^+$	$[M-H]^-$	Others
PubChem CID:<br>11294<br><br>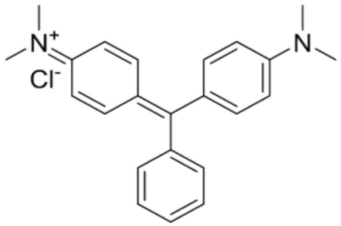	pKa (strongest basic):<br>4.52<br><br>Partition coefficient (Log P): 1.287<br><br>Intrinsic solubility (mg mL^{-1}): The molecule cannot be neutralized and the calculation is not defined for molecules with non-zero charge					
Malachite green metabolite (Leucomalachite green)<br><br>CAS registration number:<br>129-73-7<br><br>ChemSpider ID:	Molecular formula:<br>$C_{23}H_{26}N_2$<br><br>Monoisotopic mass (Da): 330.2091<br><br>pKa (strongest acidic): -	$[M+H]^+$	331.2169	239.1543;<br>223.1230;<br>165.0699;<br>153.0699;<br>152.0621		

Group/Class - Pharmacologically active Substance	Physicochemical properties	Monitored ion	Theoretical exact mass MS (*m/z*)	Fragment ions (*m/z*) MS^2		
				[M+H]$^+$	[M-H]$^-$	Others
60551<br><br>PubChem CID: 67215<br><br>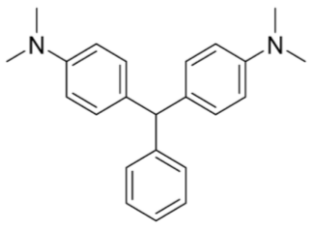	pKa (strongest basic): 5.17<br><br>Partition coefficient (Log P): 5.702<br><br>Intrinsic solubility (mg mL^{-1}): -4.376 (Moderate)					

Values were calculated using online Data taken from Chemicalize: log P: octanol/water partition coefficient [107]; pKa: acid/base dissociation constants data taken from computational predictions from [108]; solubility information: low: if < 0.01 mg mL^{-1}; moderate: if is between 0.01 and 0.06 mg mL^{-1} and high if > 0.06 mg mL^{-1} [109]. Chemical structures were drawn using Chemdraw version 18.1 [110]. Identification numbers were taken from Chemspider [111] and PubChem [112].

Table S5. Retention time of veterinary drugs and their metabolites evaluated by Q-Exactive Orbitrap HRMS.

**Group/Class - Pharmacologically active Substance**	**Retention time (min)**					
	Method A		Method B*			
			Aqueous extract (Tube 1)		Organic extract (Tube 2)	
	HESI$^+$	HESI$^-$	HESI$^+$	HESI$^-$	HESI$^+$	HESI$^-$
**Antimicrobials/Aminoglycosides**						
Amikacin						
Apramycin						
Dihydrostreptomycin						
Spectinomycin						
Streptomycin						
Gentamicin (Gentamicin C_1)						
Gentamicin (Gentamicin C_{1a})						
Gentamicin (Gentamicin C_2-C_{2a})						
Hygromycin (Hygromycin B)						
Kanamycin (Kanamycin A)						
Neomycin (Neomycin B)						
Tobramycin						
**Antimicrobials/Amphenicols**						
Chloramphenicol				8.53		
Florfenicol		7.19	7.27			
Thiamphenicol		6.47		4.92		
**Antimicrobials/Betalactams**						
Amoxicillin						
Ampicillin	6.85		7.39			
Cephalexin	6.62		5.07			
Cefalonium			4.89			
Cephapirin	5.78		3.82			
Cefazolin			6.26			
Cefoperazone			8.15			
Cefquinome	6.17		5.32			
Cloxacillin	9.55[a]		11.08[b]			
Dicloxacillin	9.85[b]		11.36			
Nafcillin	9.84[c]		9.20			
Oxacillin	9.38[a]		9.79[c]			
Benzylpenicillin	7.26		9.96			
Phenoxymethylpenicillin/Phenoxymethyl V penicillin	9.45[a]		10.48[c]			
**Antimicrobials/Lincosamides**						
Clindamycin	8.47		9.41			

**Group/Class - Pharmacologically active Substance**	**Retention time (min)**					
	Method A		Method B*			
			Aqueous extract (Tube 1)		Organic extract (Tube 2)	
	HESI$^+$	HESI$^-$	HESI$^+$	HESI$^-$	HESI$^+$	HESI$^-$
Lincomycin		6.31	4.54			
**Antimicrobials/Macrolides**						
Azithromycin			9.34			
Erythromycin (Erythromycin A-H_2O)	9.20		10.58[d]			
Spiramycin (Spiramycin I)	7.35[e]		9.34			
Tilmicosin		6.30	9.92			
Tylosin (Tylosin A)	8.84		10.39			
**Antimicrobials/Quinolones-Fluoroquinolones**						
Nalidixic acid	8.89		10.09			
Oxolinic acid	8.12		8.90			
Ciprofloxacin	6.62		6.29			
Danofloxacin	6.63		6.78			
Difloxacin	6.83		7.72			
Enrofloxacin	6.62		6.94			
Flumequine	9.11		10.40			
Norfloxacin	6.56		6.07			
Sarafloxacin	6.94		7.62			
**Antimicrobials/Sulphonamides**						
Sulfachlorpyridazine	6.99		6.49			
Sulfadiazine	5.54		2.41			
Sulfadimethoxine	7.94		9.52			
Sulfadoxine	7.15			7.21		
Sulfisoxazole/Sulfafurazole	7.25		8.03			
Sulfamerazine		6.19	3.97			
Sulfamethazine/Sulfadimidine	6.68		5.19			
Sulfathiazole	5.86		3.65			
Sulfamethoxazole	7.07		7.19			
Sulfaquinoxaline	8.09		9.67			
**Antimicrobials/Tetracyclines**						
Chlortetracycline	7.82		8.51			
Doxycycline	6.80		6.21			
Oxytetracycline		6.93				
Tetracycline	8.35		6.21			
**Antimicrobials/Others**						
Bromhexine	9.06		10.41			
Dapsone	6.47		6.12			

Group/Class - Pharmacologically active Substance	Retention time (min)					
	Method A		Method B*			
			Aqueous extract (Tube 1)		Organic extract (Tube 2)	
	HESI+	HESI-	HESI+	HESI-	HESI+	HESI-
Rifampicin		9.90		11.47		
Tiamulin	8.86		10.64			
**Antimicrobials Prospect/Quinolones-Fluoroquinolones**						
Marbofloxacin	6.23		5.60			
**Antimicrobials Prospect/Macrolides**						
Josamycin/Leucomycin A_3			11.04			
Leucomycin/Leucomycin A_5	8.98		10.61			
Tildipirosin	5.43				9.23	
Tulathromycin (Tulathromycin A)	6.30		5.44			
**Antimicrobials Prospect/ Sulfonamides**						
Phthalylsulfathiazole		7.48	8.62			
Sulfacetamide/*N*-Sulfanilylacetamide	5.62			1.46		
Sulfamethoxypyridazine	6.78		5.75			
**Antimicrobials Prospect/Others**						
Diminazene	5.39					
Fosfomycin						
Isoniazid	1.35					
Novobiocin					10.58	
Rifaximin		10.35		11.75		
**Antibiotic Growth Promoters**						
Avilamycin (Dichloroisoeverninic acid)		7.99		7.06		
Halquinol/Chlorhydroxyquinoline (7-Chloro-8-quinolinol)					9.98	
Halquinol/Chlorhydroxyquinoline (5,7-Dichloro-8-quinolinol/Chloroxine)					10.58	
Halquinol/Chlorhydroxyquinoline (5-Chloro-8-hydroxyquinoline)					10.16	
Virginiamycin (Virginiamycin M_1)	9.60[a]		10.96			
**Anticoccidials**						
Amprolium						
Clopidol	6.40		2.94			
Diaveridine	6.12		4.68			
Decoquinate		13.46	13.49			
Diclazuril		11.02				10.55
Ethopabate	8.65[a]		9.33			
Lasalocid (Lasalocid A)						13.12
Maduramicin						

**Group/Class - Pharmacologically active Substance**	**Retention time (min)**					
	Method A		Method B*			
			Aqueous extract (Tube 1)		Organic extract (Tube 2)	
	HESI$^+$	HESI$^-$	HESI$^+$	HESI$^-$	HESI$^+$	HESI$^-$
Monensin (Monensin A)						
Narasin (Narasin A)						
Nicarbazin (4,4′-Dinitrocarbanilide - DNC)		10.69				10.37
Robenidine	9.87				10.18	
Salinomycin	13.08[a]			13.84		
Toltrazuril						
Trimethoprim	6.28				9.35	
**Anti-inflammatories/Steroidal**						
Prednisolone			10.03			
Prednisone			10.02			
**Anti-inflammatories Prospect/Steroidal**						
Isoflupredone acetate/9-Fluoroprednisolone acetate						
Flumetasone						
**Anti-Inflammatory/Non-Steroidal**						
Mefenamic acid		11.99				10.70
Tolfenamic acid		11.06				10.41
Carprofen		11.08		11.93		
Ketoprofen		9.96	11.22			
Diclofenac				12.11		
Flunixin		11.05		11.13		
Indomethacin				12.12		
Meloxicam		9.74	11.24			
Naproxen						
Nimesulide		9.82		11.63		
Piroxycam	8.66		10.16			
Propyphenazone	9.19		10.38			
**Antiparasitic/Avermectins**						
Avermectin B_{1a}/Abamectin B_{1a}						
Doramectin						
Emamectin (Emamectin B_{1a})	11.48					
Eprinomectin (Eprinomectin B_{1a})						
Ivermectin B_{1a}/22,23-Dihydroavermectin B_1						
Moxidectin					11.73	
**Antiparasitic/Benzimidazoles**						
Albendazole	10.04		10.48			

Group/Class - Pharmacologically active Substance	Retention time (min)					
	Method A		Method B*			
			Aqueous extract (Tube 1)		Organic extract (Tube 2)	
	$HESI^+$	$HESI^-$	$HESI^+$	$HESI^-$	$HESI^+$	$HESI^-$
Albendazole metabolite (Albendazole sulfone)	8.07		8.57			
Albendazole metabolite (Albendazole sulfoxide)	7.95		7.01			
Closantel		11.06		14.64		
Febantel	10.80		12.18			
Fenbendazole	10.57		11.24			
Fenbendazole metabolite (Fenbendazole sulfone)	8.66		10.16			
Flubendazole	9.68		10.58			
Flubendazole metabolite (2-Aminoflubendazole)	8.07		8.79			
Levamisole	5.83		3.52			
Mebendazole	9.44		10.34			
Oxibendazole	8.96		9.11			
Oxfendazole	8.85		9.21			
Thiabendazole	7.10		4.53			
Triclabendazole		11.84			10.67	
**Antiparasitic/Phenylpyrazoles**						
Fipronil		10.90		12.65		
Fipronil metabolite (Fipronil sulfone)		11.14		13.04		
**Antiparasitic/Nitroimidazoles**						
Dimetridazole						
Dimetridazole metabolite (Hydroxydimetridazole/HMMNI)						
Ipronidazole	7.87		6.70			
Ipronidazole metabolite (Hydroxy Ipronidazole)	7.28					
Metronidazole						
Metronidazole metabolite (Hydroxymetronidazole)						
Ronidazole/1-Methyl-2-carbamoyloxymethyl-5-nitroimidazole						
**Antiparasitic/Isoquinoline-pyrazines**						
Praziquantel	10.16		11.24			
**Beta-Agonists**						
Cimaterol						
Clenbuterol						
Ractopamine						
Salbutamol	5.26					
Zilpaterol						
**Sedatives**						

Group/Class - Pharmacologically active Substance	Retention time (min)					
	Method A		Method B*			
			Aqueous extract (Tube 1)		Organic extract (Tube 2)	
	$HESI^+$	$HESI^-$	$HESI^+$	$HESI^-$	$HESI^+$	$HESI^-$
Acepromazine	8.88		10.39			
Azaperol	7.04		6.12			
Azaperone	7.44		7.10			
Carazolol	7.58		9.19			
Chlorpromazine	9.61		10.94			
**Prospecting sedatives**						
Xylazine	7.01		6.51			
**Corants**						
Gentian violet/Crystal violet	9.68[f]		11.87[f]			
Gentian violet/Crystal violet metabolite (Leucocrystal Violet)			10.13			
Malachite green	9.12[f]					
Malachite green metabolite (Leucomalachite green)	12.56		12.17			

Adducts: [a]$[M+Na]^+$; [b]$[(M-H-CH_3OH)]^+$; [c]$[(M+H_2-CO)+H]^+$; [d]$[M-H_2O+H]^+$; [e]$[M+2H]^{2+}$; [f]$[M]^+$. Abbreviations: $HESI^+$: heated electrospray ionization source operated in positive ion mode; $HESI^-$: heated electrospray ionization source operated in negative ion mode; min: minute. *In method B, the analytes marked in gray were analyzed by the organic extract.

Figure S1. General flowchart of the sample preparation procedure according to method A.

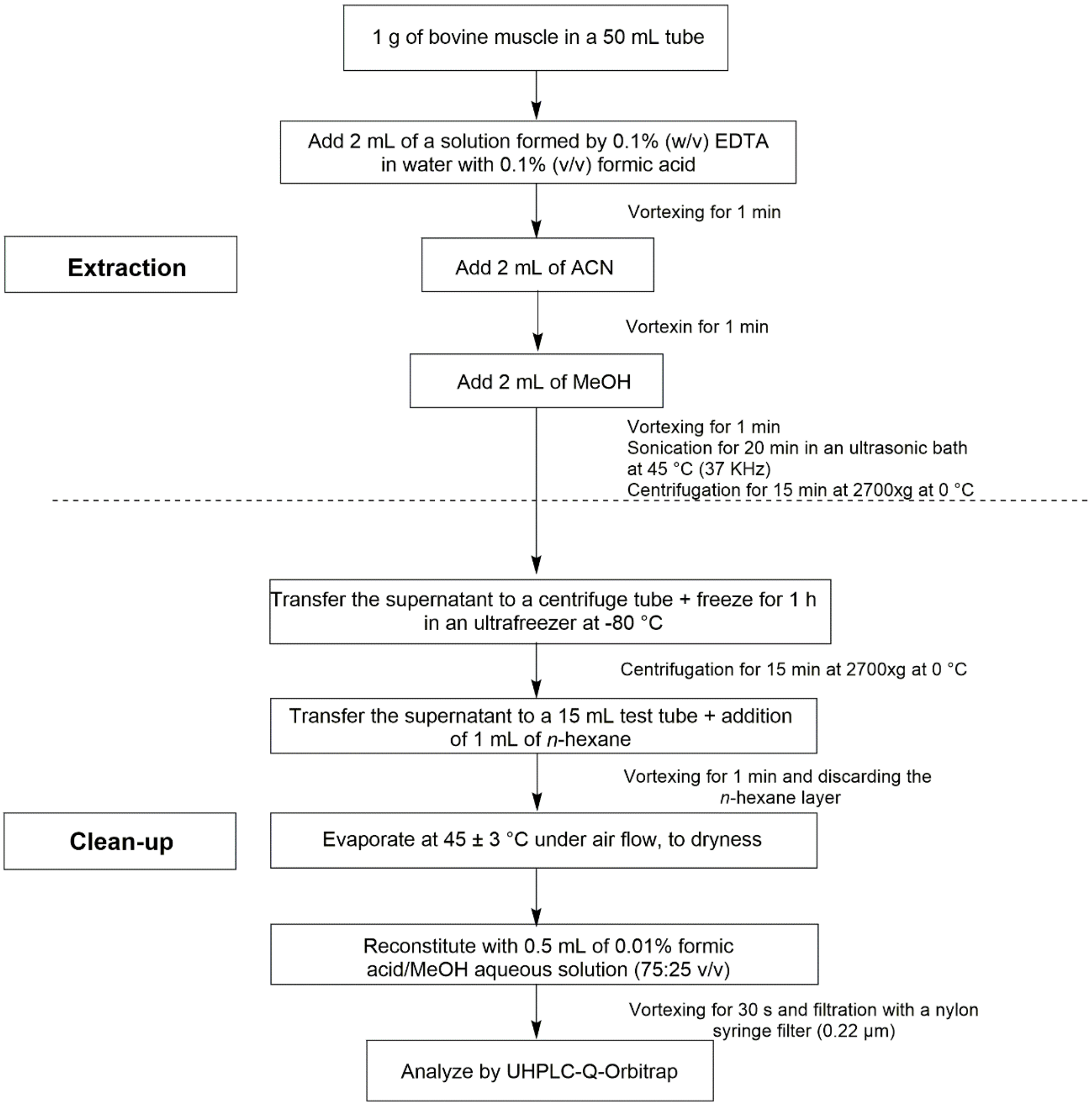

Prepared according to the modified method described by Dasenaki et al. [57].

Figure S2. General flowchart of the sample preparation procedure according to method B.

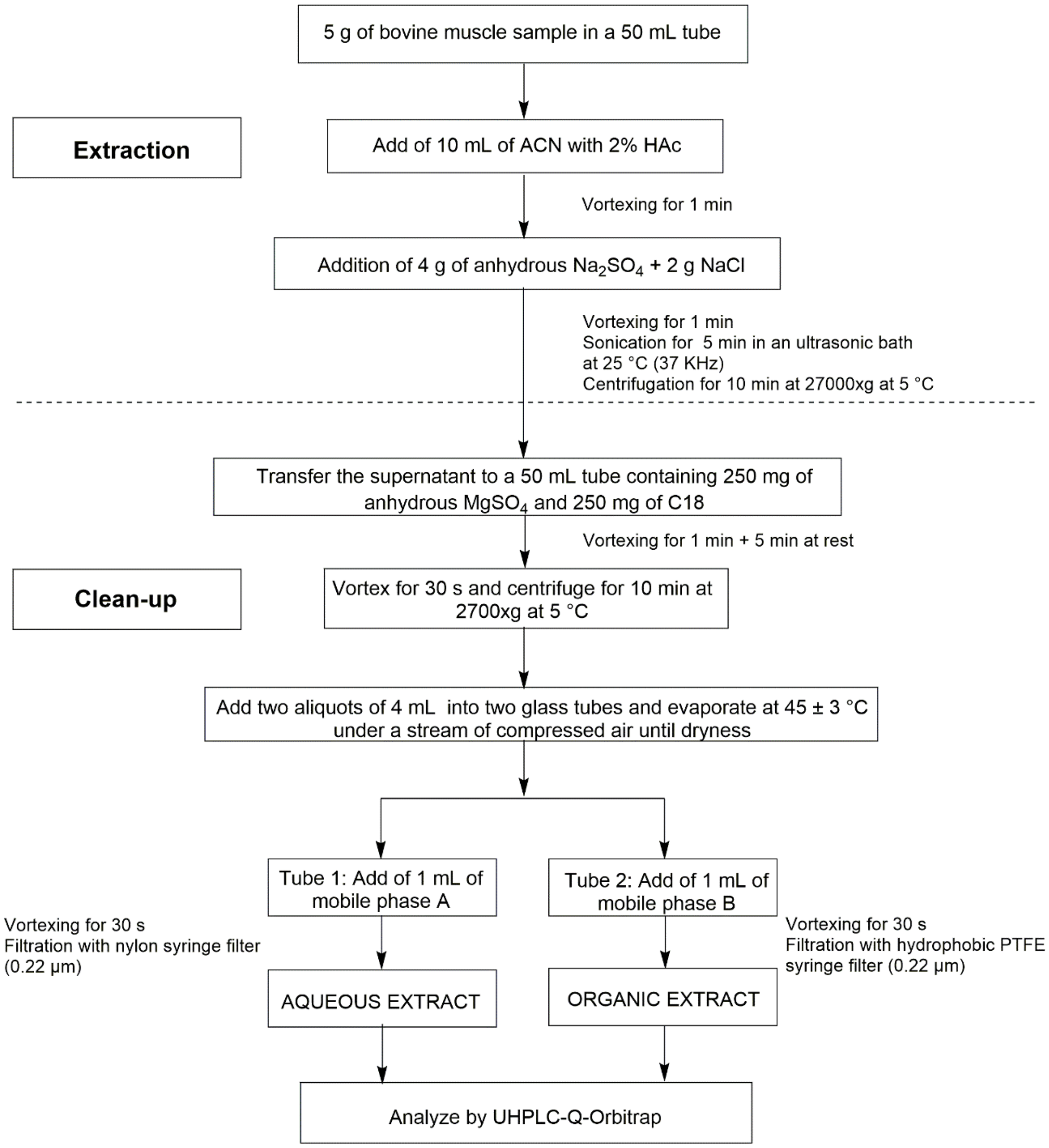

Prepared according to the modified method described by Silva et al. [22].

Figure S3. Analyte identification criteria [22,57].

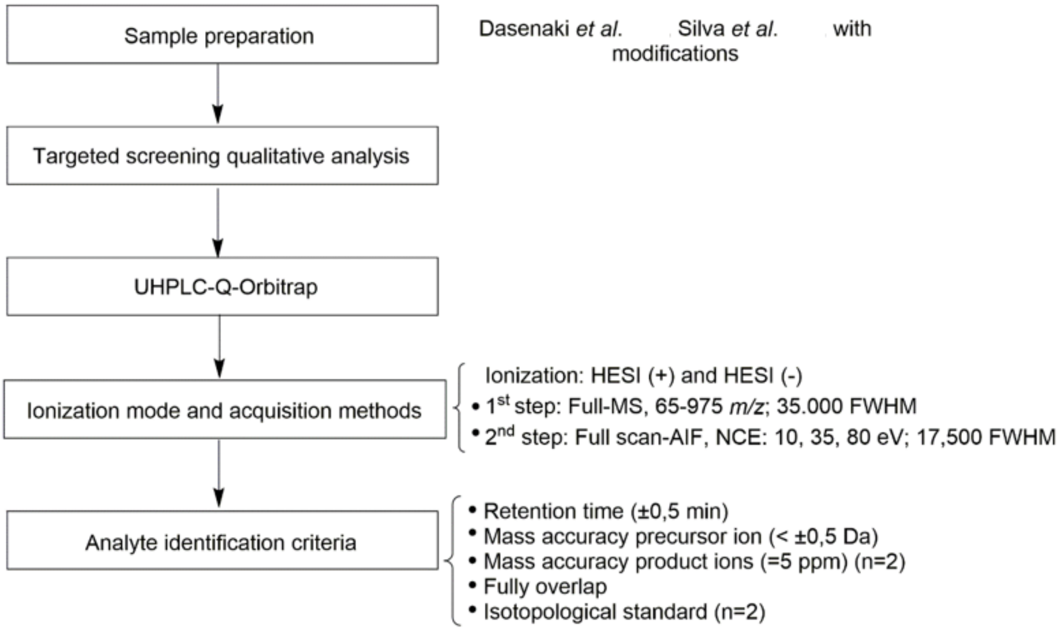

Figure S4. Examples of extracted mass traces of a spiked bovine muscle tissue at 2 MRL according to the established criteria for analyte identification.

Trimethoprim $[M+H]^+$

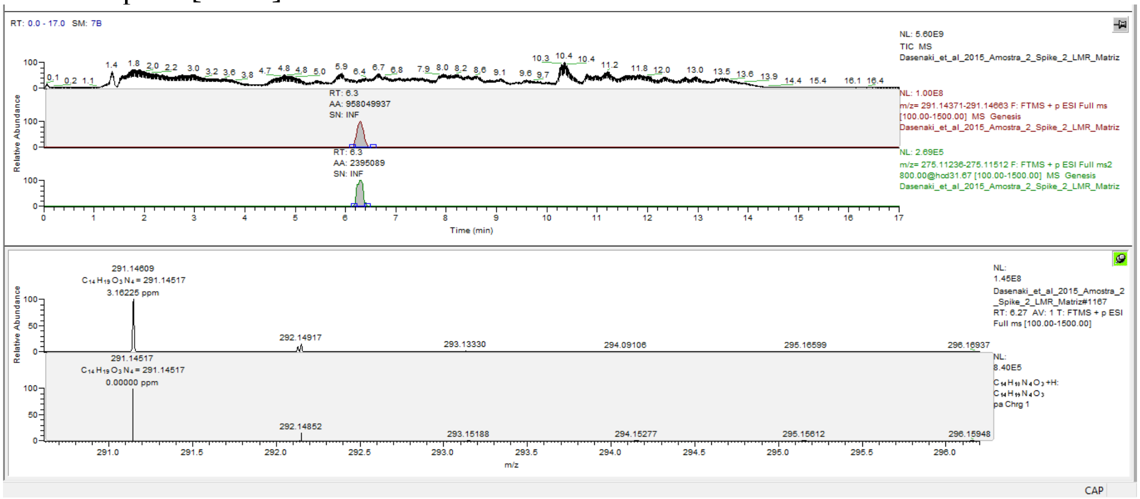

Virginiamycin $M_1[M+Na]^+$

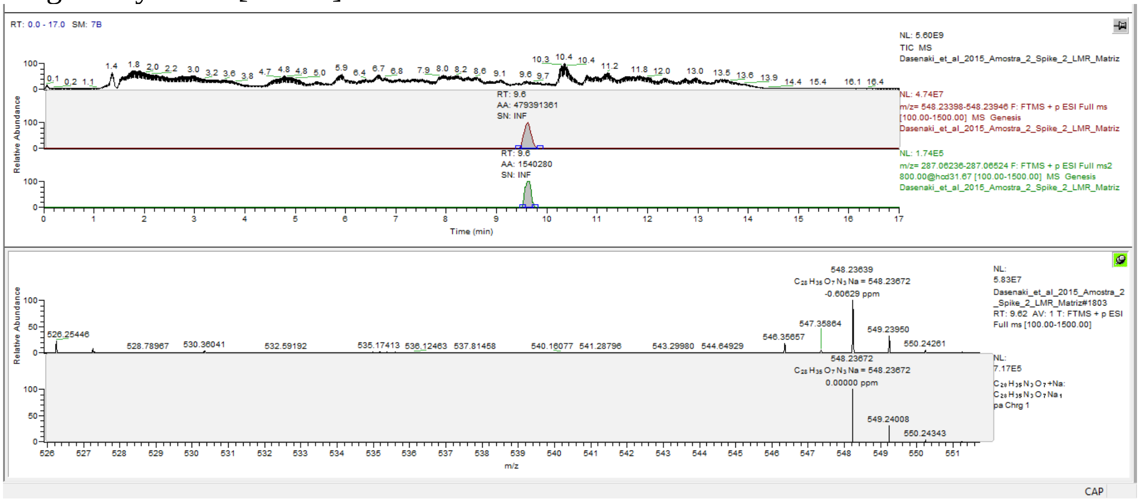

Dichloroisoeverninic acid $[M-H]^-$

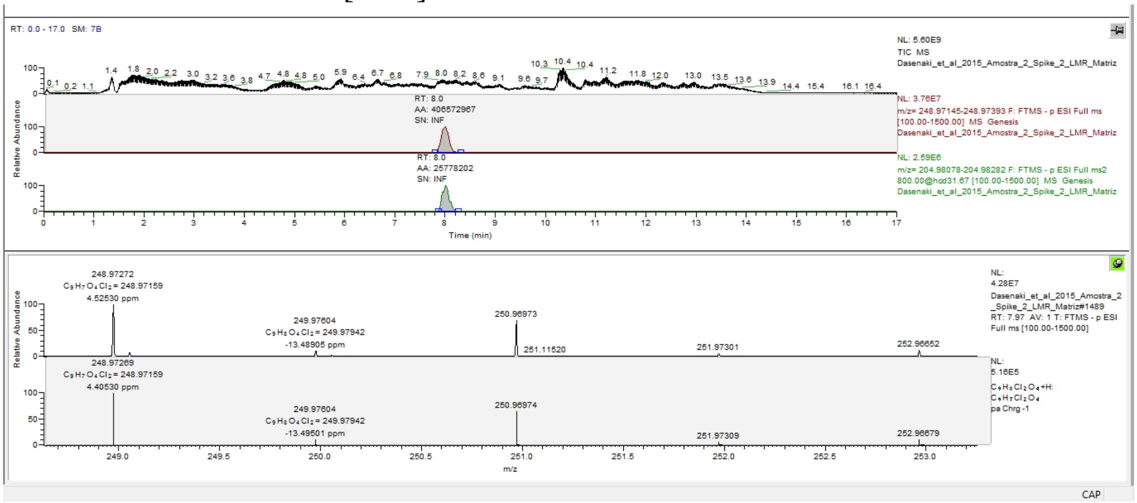

Supplement: Supplementary file 1 [file molecules-28-04150-s001.zip › molecules-2308580-supplementary.pdf]
